# DNA duplication in *Burkholderia thailandensis* induces biofilm formation by activating a two-component regulatory system

**DOI:** 10.1371/journal.pgen.1011528

**Published:** 2025-05-20

**Authors:** Lillian C. Lowrey, Katlyn B. Mote, Peggy A. Cotter

**Affiliations:** Department of Microbiology and Immunology, The University of North Carolina at Chapel Hill, School of Medicine, Chapel Hill, North Carolina, United States of America; Michigan State University, UNITED STATES OF AMERICA

## Abstract

*Burkholderia thailandensis* strain E264 (*Bt*E264) and close relatives stochastically duplicate a 208.6 kb region of chromosome I via RecA-dependent recombination between two nearly identical insertion sequence elements. Because homologous recombination occurs at a constant, low level, populations of *Bt*E264 are always heterogeneous, but cells containing two or more copies of the region (Dup+) have an advantage, and hence predominate, during biofilm growth, while those with a single copy (Dup–) are favored during planktonic growth. Moreover, only Dup+ bacteria form ‘efficient’ biofilms within 24 hours in liquid medium. We determined that duplicate copies of a subregion containing genes encoding an archaic chaperone-usher pathway pilus (*csuFABCDE*) and a two-component regulatory system (*bfmSR*) are necessary and sufficient for generating efficient biofilms and for conferring a selective advantage during biofilm growth. BfmSR functionality is required, as deletion of either *bfmS* or *bfmR*, or a mutation predicted to abrogate phosphorylation of BfmR, abrogates biofilm formation. However, duplicate copies of the *csuFABCDE* genes are not required. Instead, we found that BfmSR controls expression of *csuFABCDE* and *bfmSR* by activating a promoter upstream of *csuF* during biofilm growth or when the 208.6 kb region, or just *bfmSR*, are duplicated. Single cell analyses showed that duplication of the 208.6 kb region is sufficient to activate BfmSR in 75% of bacteria during planktonic (BfmSR ‘OFF’) growth conditions. Together, our data indicate that the combination of deterministic two-component signal transduction and stochastic, duplication-mediated activation of that TCS form a bet-hedging strategy that allows *Bt*E264 to survive when conditions shift rapidly from those favoring planktonic growth to those requiring biofilm formation, such as may be encountered in the soils of Southeast Asia and Northern Australia. Our data highlight the positive impact that transposable elements can have on the evolution of bacterial populations.

## Author summary

Transposable elements naturally accumulate within genomes in all kingdoms of life. When present in the same orientation, a pair of homologous elements can act as substrates for DNA recombination reactions that can duplicate and delete intervening sequences – giving rise to genetically heterogenous populations. We showed here that *Burkholderia thailandensis* strain E264 uses this mechanism to amplify genes encoding a two-component regulatory system and an archaic chaperone usher pathway pilus, priming the cells for rapid biofilm formation. The formation of a small subpopulation of biofilm-ready bacteria serves as a bet-hedging strategy, ensuring overall population survival should conditions change rapidly from those in which planktonic growth is optimal to those in which adherence and biofilm formation is required.

## Introduction

*Burkholderia thailandensis* is a saprophytic environmental bacterium that has predominantly been isolated from tropical regions of Northern Australia and Southeastern Asia [1,2]. In these aquatic and soil environments, it must constantly adapt to fluctuations in conditions and compete with other microbes.

As is true for organisms in all kingdoms of life, *B. thailandensis* has accumulated insertion sequences and transposons – parasitic genetic elements capable of moving between DNA sequences – throughout its genome [3]. In *B. thailandensis* strain E264 (*Bt*E264) and close relatives (e.g., strains 2002721643, 2002721723, and BPM [4,5]), two nearly identical IS*2*-like elements called ISα and ISβ bound a 208.6 kb region of DNA in chromosome I (hereafter referred to as ‘the 208.6 kb region’) [6]. We recently discovered that these IS elements can act as substrates for RecA-dependent homologous recombination during replication, resulting in tandem duplication of the 208.6 kb region [7]. Reciprocally, a single homologous recombination event between any of the sequences that are duplicated in strains with tandem repeats of the 208.6 kb region results in excision of the intervening sequences and resolution of the region back to a single copy [7]. Because these recombination reactions occur at a low but constant level, *Bt*E264 populations always contain some cells with (Dup+) and some cells without (Dup–) duplicate copies of the 208.6 kb region, the proportion of which varies depending on whether duplication of the region confers a growth advantage or disadvantage under the specific growth condition [7].

Amplification of DNA sequences (increasing gene dosage) can alter cell phenotypes [8]. A well-studied example is increased antibiotic resistance, which can occur by amplification of genes encoding antibiotic targets, efflux pumps, or antibiotic-modifying enzymes [9]. Copy number of the 208.6 kb region influences phenotypes in *Bt*E264, impacting colony morphology, pigmentation, and most notably, the rate of biofilm formation [7]. When M63 minimal medium is inoculated with *Bt*E264 that is predominantly Dup–, a visible biofilm forms on the walls of the test tube (corresponding to the air-liquid interface during culture) after four days. By contrast, when M63 is inoculated with *Bt*E264 that is predominantly Dup +, a visible biofilm forms within 24 hours. We refer to this phenomenon as ‘efficient’ biofilm formation [7]. Moreover, regardless of whether the cultures are initiated with predominantly Dup– or Dup+ bacteria, Dup+ bacteria quickly predominate in the biofilm that forms and Dup– bacteria predominate in the liquid medium [7]. These data indicate a strong selective advantage for Dup+ bacteria during biofilm growth and a strong selective advantage for Dup– bacteria during planktonic growth. We previously posited that the generation of Dup+ and Dup– subpopulations is a bet-hedging strategy that allows *Bt*E264 to survive in unpredictable environments that quickly switch between those favoring biofilm or planktonic lifestyles [7].

The 208.6 kb region contains 158 predicted genes. We hypothesized that increasing the dosage of one or more of these genes promotes rapid biofilm formation. Our goals were to identify which gene(s) within the 208.6 kb region, when duplicated, confer this efficient biofilm phenotype, and to investigate the underlying mechanism.

## Results

### Duplicate copies of DNA containing genes predicted to encode an archaic chaperone-usher pilus system, a small hypothetical protein, and a two-component regulatory system are sufficient to promote efficient biofilm formation

**Fig 1:**
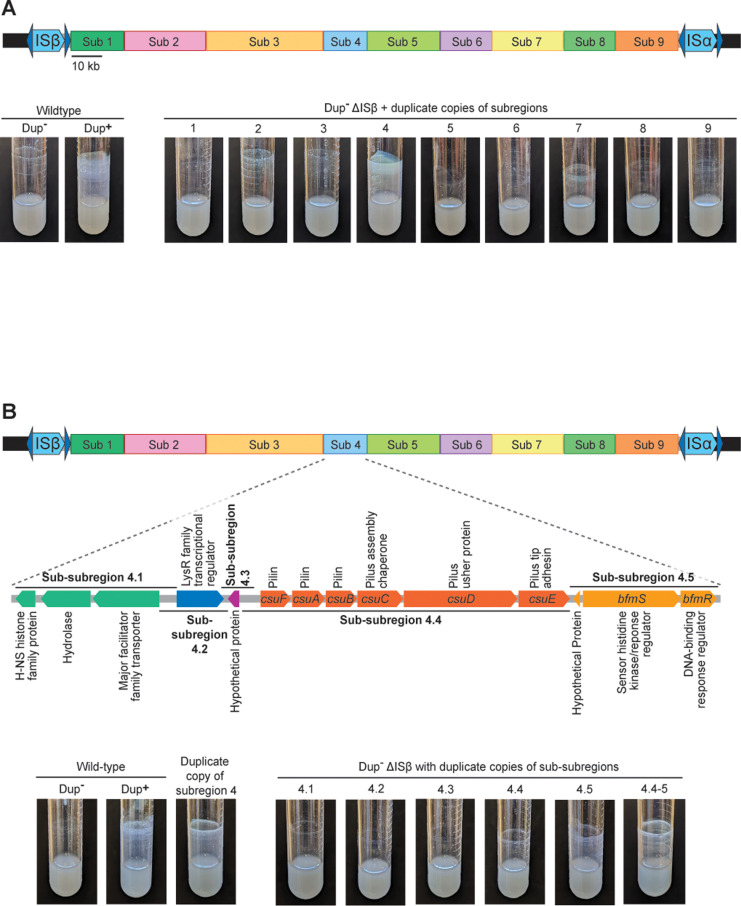
Duplicate copies of a subregion of DNA containing *csuFABCDE, iou, and bfmSR,* is sufficient to promote efficient biofilm formation. (A) Top: Schematic of the duplicating region divided into nine subregions. Subregion boundaries were designed to prevent interruption of coding sequences or separation of coding sequences from their putative promoters. Bottom: Images of overnight cultures comparing the biofilm-forming ability of wild-type *B*tE264 to strains with duplicate copies of subregions 1 - 9. (B) Top: Schematic of the fourteen predicted genes located within subregion 4 and the boundaries of sub-subregions 4.1 – 4.5. Bottom: Images of overnight cultures comparing the biofilm-forming ability of wild-type *Bt*E264 and a strain with a duplicate copy of subregion 4 to strains with duplicate copies of sub-subregions 4.1 – 4.5 and 4.4-5. For all schematics, genes and regions are drawn to scale except ISα and ISβ, which are 1.2 kb and would not be visible if not enlarged.

To identify the gene(s) responsible for efficient biofilm formation, we first divided the 208.6 kb region into nine subregions (Fig 1A). The bounds of each region were chosen based on predicted gene function and designed to keep predicted open reading frames (ORFs) and operons intact. Each subregion was cloned into a plasmid using a modified plasmid rescue technique (S1 & S2 Figs). Briefly, we integrated a pair of I-*Sce*I restriction endonuclease site-containing suicide plasmids into the chromosome at the boundaries of each subregion, then isolated genomic DNA from each strain, digested the DNA with I-*Sce*I, ligated the isolated DNA, and transformed *E. coli*, selecting for kanamycin resistance (Km^R^). The composition and integrity of the resulting plasmids were confirmed by DNA sequence analysis. We then introduced each subregion-containing suicide plasmid into separate *Bt*E264 strains lacking ISβ (ΔISβ) (S3 Fig), which were therefore unable to duplicate the entire 208.6 kb region, resulting in strains in which only a single subregion was duplicated. We tested each strain for its ability to form biofilms after 24 hours growth in M63 medium. Only the strain with a duplicate copy of subregion 4 formed efficient biofilms (Fig 1A), indicating that the gene(s) sufficient for promoting efficient biofilm formation when present in multiple copies is/are within subregion 4.

Subregion 4 contains fourteen genes (Fig 1B). Thirteen are annotated in the reference genome and are predicted to encode: an H-NS histone family protein, a hydrolase, a major facilitator family transporter, a LysR family transcriptional regulator, a protein of unknown function, an archaic chaperone-usher pathway (CUP) pilus system, and a two-component regulatory system (TCS). Closer investigation revealed an additional ORF between the pilus- and TCS-encoding operons that is oriented in the opposite direction and has the potential to encode a small protein of unknown function. We divided subregion 4 into five sub-subregions (Fig 1B), constructed suicide plasmids containing a single sub-subregion, and introduced these plasmids into the ΔISβ strain. Strains with a duplicate copy of sub-subregion 4.4 or 4.5 formed a minimal biofilm at 24 hours, but neither phenocopied the biofilm formed by strains with a duplication of the full 208.6 kb region or a duplication of subregion 4. However, integration of a plasmid containing both sub-subregions 4.4 and 4.5 together (sub-subregion 4.4-5), resulted in a strain that formed efficient biofilms (Fig 1B). The genes in sub-subregion 4.4-5 that are predicted to encode an archaic CUP pilus system (BTH_I2681-BTH_I2676) are homologs of genes required for biofilm formation in *Pseudomonas aeruginosa* (where they are called *cup* genes) and *Acinetobacter baumanii* (where they are called *csu* genes) [10,11]. For consistency, we named these genes *csuFABCDE*. The genes encoding a two-component regulatory system (BTH_I2675-BTH_I2674) also have homologs that have been implicated in biofilm formation in *Burkholderia pseudomallei* [12,13], and therefore we named them *bfmSR*. We named the previously unannotated small ORF *iou* for intergenic orf of unknown function. Our data indicate that duplicate copies of the subregion of DNA containing these genes is sufficient for generating the efficient biofilm phenotype.

### *csuFABCDE* and *bfmSR*, but not *iou*, are required for efficient biofilm formation

To determine if *csuFABCDE*, *iou*, and/or *bfmSR* are required for efficient biofilm formation, we wished to compare *csuFABCDE*, *iou*, and *bfmSR* mutants with wild-type bacteria – all containing duplicate copies of the 206.8 kb region – for their ability to form efficient biofilms. To do so, we used one of the ‘fragmented gene’ reporter systems that we developed and described previously [7]. In these systems, we replaced the IS*2*-like elements bounding the 208.6 kb region with a pair of incomplete, overlapping reporter gene fragments. The fragment replacing ISα is missing the 5′ end of the reporter gene and the fragment replacing ISβ is missing the 3′ end of the reporter gene. Homologous recombination within the overlapping central part of the gene fragments results in duplication of the region and a functional copy of the reporter gene (conferring antibiotic resistance, fluorescence, or the production of β-glucuronidase) present at the junction between the two duplicated regions [7]. These reporters allow us to select or screen for bacteria containing a duplication of the intervening sequences.

**Fig 2:**
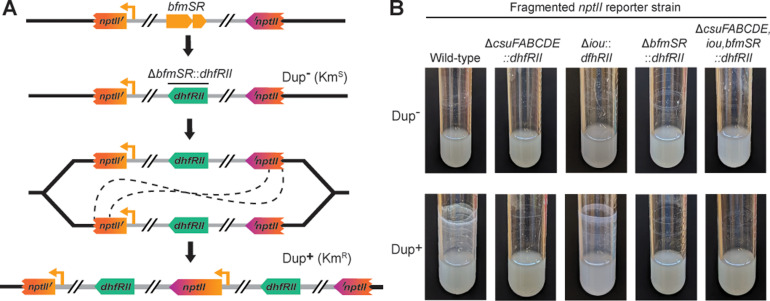
*csuFABCDE* and *bfmSR* are required for efficient biofilm formation. (A) Schematic of the fragmented *nptII* gene system used to duplicate the 208.6 kb region in strains lacking sequences of interest, such as *bfmSR*, to test requirements for efficient biofilm formation. Genes of interest were replaced with *dhfRII* in a Dup– (Km^S^) fragmented *nptII* reporter stain. Using kanamycin selection, cells that had spontaneously duplicated the region could be isolated. (B) Images of 24-hour cultures comparing the biofilm-forming ability of fragmented *nptII* strains with deletions of *csuFABCDE*, *iou*, and/or *bfmSR* with and without duplicate copies of the 208.6 kb region.

Here, we used the fragmented *nptII* (encoding kanamycin resistance (Km^R^)) reporter strain (Fig 2A) so that we could select Dup+ (Km^R^) bacteria. To mutate *csuFABCDE*, *iou*, and/or *bfmSR*, we replaced them with *dhfRII* (encoding trimethoprim resistance (Tmp^R^)) in the fragmented *nptII* reporter strain, using bacteria that were Km^S^ and hence contained only a single copy of the region. We then selected Km^R^ derivatives (in which the region had duplicated) and compared Dup– (Km^S^) and Dup+ (Km^R^) bacteria for their ability to form efficient biofilms. Dup+ wild-type and *iou* mutant bacteria formed similar biofilms after 24 hours, while the *csuFABCDE* and *bfmSR* mutants failed to form biofilms (Fig 2B), indicating that *csuFABCDE* and *bfmSR*, but not *iou*, are required for efficient biofilm formation.

### *csuFABCDE* and *bfmSR* are sufficient and required for duplication to provide a selective advantage during biofilm growth

**Fig 3:**
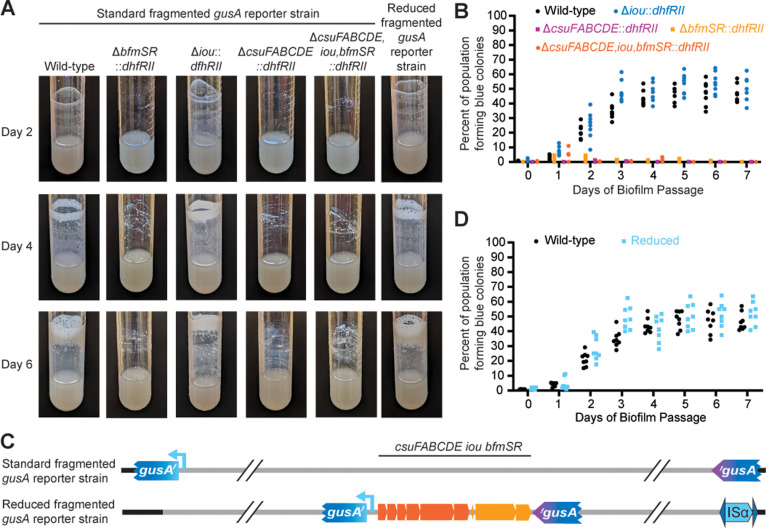
*csuFABCDE* and *bfmSR* are sufficient and required for duplication to provide a selective advantage during biofilm growth. (A) Images of culture tube biofilms following two, four, and six days of serial biofilm passage of the fragmented *gusA* reporter strains that are otherwise wild-type or have *csuFABCDE*, *iou*, and/or *bfmSR* deleted through replacement with *dhfRII.* Graph of the percentage of blue colonies grown from cells scraped from the edge of the culture tube daily during a seven-day serial biofilm passage experiment comparing (B) standard fragmented *gusA* reporter strains lacking *csuFABCDE, iou*, and/or *bfmSR*, and (D) the standard and reduced fragmented *gusA* reporter strains. (C) Schematic of the fragmented *gusA* reporter strains wherein homologous recombination duplicates the entire 208.6 kb region (Standard) or just the 9.8 kb subregion containing *csuFABCDE,iou,bfmSR* (Reduced).

We showed previously that Dup+ bacteria have a selective advantage during biofilm growth using a fragmented *gusA* (encoding β-glucuronidase) reporter system [7]. With this system, Dup+ (*gusA*^+^) bacteria form blue colonies when plated on medium containing X-gluc, allowing us to screen, rather than select, for Dup+ cells, so that we can determine the proportion of Dup+ and Dup– bacteria in the population. We tested the hypothesis that *csuFABCDE* and *bfmSR* are responsible for the selective advantage conferred by duplication of the 208.6 kb region during biofilm growth using this fragmented *gusA* reporter system. We replaced *csuFABCDE, iou*, and/or *bfmSR* with *dhfRII* in Dup– fragmented *gusA* reporter bacteria, then grew these strains to develop biofilms over multiple days, replacing the liquid medium (and any cells it contained) every 24 hours with fresh, sterile medium. The wild-type and Δ*iou* strains formed visible biofilms within two days that increased in robustness as the experiment progressed, forming very thick biofilms by day 6 (Fig 3). By contrast, the Δ*csuFABCDE*::*dhfRII*, Δ*bfmSR*::*dhfRII*, and Δ*csuFABCDE,iou,bfmSR*::*dhfRII* strains were unable to form biofilms, even after 6 days. (Although some aggregation of bacteria and cell debris could be seen on the test tubes at days 4 and 6 post-inoculation, the cultures were clearly not forming biofilms like the wild-type and ∆*iou* mutant strains.) We showed previously that mutants that are unable to duplicate the region, such as strains with deletion mutations in ISα or ISβ, can form biofilms after 6 days of incubation that are indistinguishable from those formed by wild-type bacteria [7]. The data in Fig 3, therefore, show that *csuFABCDE* and *bfmSR* are not only required for efficient biofilm formation, they are also required for longer-term ‘inefficient’ biofilm formation, i.e., both sets of genes are absolutely required for biofilm formation, at least under these experimental conditions.

When sampling from the biofilms (wild-type and ∆*iou::dhfRII* strains)*,* we observed that within two days, nearly 25% of the population formed blue colonies on X-Gluc-containing medium, indicating that a quarter of the biofilm was composed of Dup+ cells (Fig 3B). At three days and longer, the percentage of blue colonies, and thus Dup+ cells, was nearly 50%. These data are consistent with those we reported for wild-type *Bt*E264 previously [7]. These data indicate that *iou* is not required for the advantage that duplication of the region provides during biofilm growth (Fig 3B). For the Δ*csuFABCDE*::*dhfRII*, Δ*bfmSR*::*dhfRII*, and Δ*csuFABCDE,iou,bfmSR*::*dhfRII* strains, nearly all the bacteria that we could obtain from the walls of the test tubes formed white colonies on X-Gluc-containing medium, indicating that they were Dup–. *csuFABCDE* and *bfmSR,* therefore, are required for the selective advantage conferred by duplication of the region during biofilm growth.

To determine if duplication of the *csuFABCDE,iou,bfmSR* gene cluster is sufficient to confer the selective advantage, i.e., none of the remaining 149 genes within the 208.6 kb region are required, we modified our fragmented *gusA* reporter system. We inserted the *gusA* gene fragments immediately flanking the *csuFABCDE,iou,bfmSR* genes in a ∆ISβ strain, so that only the *csuFABCDE,iou,bfmSR* genes could be duplicated by homologous recombination (via the *gusA* gene fragments) and not the entire 208.6 kb region (Fig 3C). When serially passaged to select for biofilm formation, the biofilm formed by the ‘reduced fragmented *gusA’* strain mirrored that of the strain with *gusA* fragments at ISα and ISβ (Fig 3A). Similarly, when sampling from the biofilm, the proportion of blue colonies increased and plateaued at nearly 50% after three days (Fig 3D). These data indicate that duplication of the *csuFABCDE,iou,bfmSR* gene cluster is sufficient for providing the selective advantage during biofilm growth.

### BfmSR TCS activity is required for efficient biofilm formation

**Fig 4:**
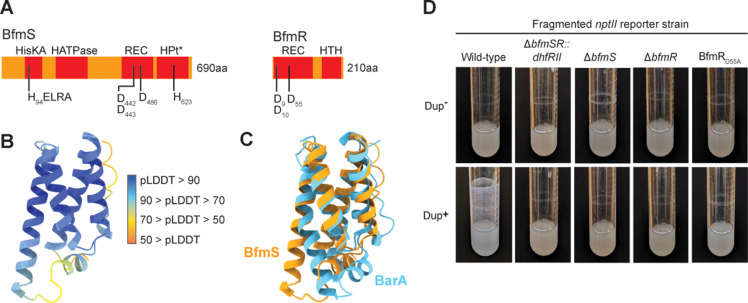
BfmSR TCS activity is required for efficient biofilm formation. (A) Schematic of the BfmS and BfmR proteins including predicted domains identified by SMART and AlphaFold2 (denoted with *). Amino acids involved in phosphoryl relay are denoted. (B) AlphaFold2 structure of BfmS_E556-S680_. The structure is colored according to per-residue predicted local distance difference test (pLDDT) scores that quantify structure confidence. (C) AlphaFold2 structure of BfmS_E556-S680_ overlaid with the *E. coli* BarA HPt domain (3IQT). (D) Images of overnight cultures comparing the biofilm forming ability of wild-type and *bfmSR*::*dhfRII*, Δ*bfmS*, Δ*bfmR*, and BfmR_D55A_ mutants in a fragmented *nptII* background strain with and without duplicate copies of the 208.6 kb region.

Analysis of the predicted amino acid sequence of BfmS with SMART domain prediction [14] and AlphaFold2 [15] reveals that BfmS is likely an unorthodox histidine kinase (i.e., it contains a histidine kinase domain, a receiver domain, and a histidine phosphotransfer domain) (Fig 4A). It is unusual in that it contains no obvious sensory input domain. Histidine Kinase A (dimerization/phosphoacceptor) (HisKA) (BfmS_A84 – G149_) and Histidine Kinase-like ATPase (HATPase) (BfmS_T196-V312_) domains were identified by SMART, and the predicted phosphorylation site is H94. A CheY-similar Receiver (REC) domain (BfmS_Y436-L551_) was also predicted by SMART, with conserved aspartic acid residues D442, D443, and D486: D486 being the site of phosphorylation. No Histidine Phosphotransfer (HPt) domain was predicted by SMART, but AlphaFold2 analysis of the C-terminal 150 amino acids of BfmS (BfmS_E556-S680_) revealed a high-confidence structure that resembles the Hpt of the *Escherichia coli* unorthodox histidine kinase BarA (Fig 4B and C).

SMART predicted REC and LuxR-type Helix-turn-helix DNA binding (HTH) domains in BfmR, indicating that BfmR belongs in the NarL/FixJ family. The BfmR REC domain likely coordinates the phosphoryl group at residues D9, D10, and D55, with D55 as the site of phosphorylation.

To determine if BfmSR TCS activity is required for Dup+ cells to produce efficient biofilms, we used allelic exchange in a fragmented *nptII* reporter strain to delete *bfmS* or *bfmR* or to change codon 55 in *bfmR* such that it encodes alanine instead of aspartic acid (BfmR_D55A_), which should prevent phosphorylation of BfmR. All three mutations, regardless of copy number of the region, abrogated the ability of the bacteria to form efficient biofilms (Fig 4D). These data indicate that BfmS and BfmR are required for efficient biofilm formation. Moreover, unless changing the Asp at position 55 to Ala (a substitution that is commonly used to prevent phosphorylation of response regulator proteins) somehow affects folding or stability of BfmR, the data indicate that BfmR activity (i.e., phosphorylation of BfmR) is required for efficient biofilm formation.

### Two copies of *bfmSR* plus the intergenic region 5′ to *csuFABCDE* are sufficient to promote efficient biofilm formation

**Fig 5:**
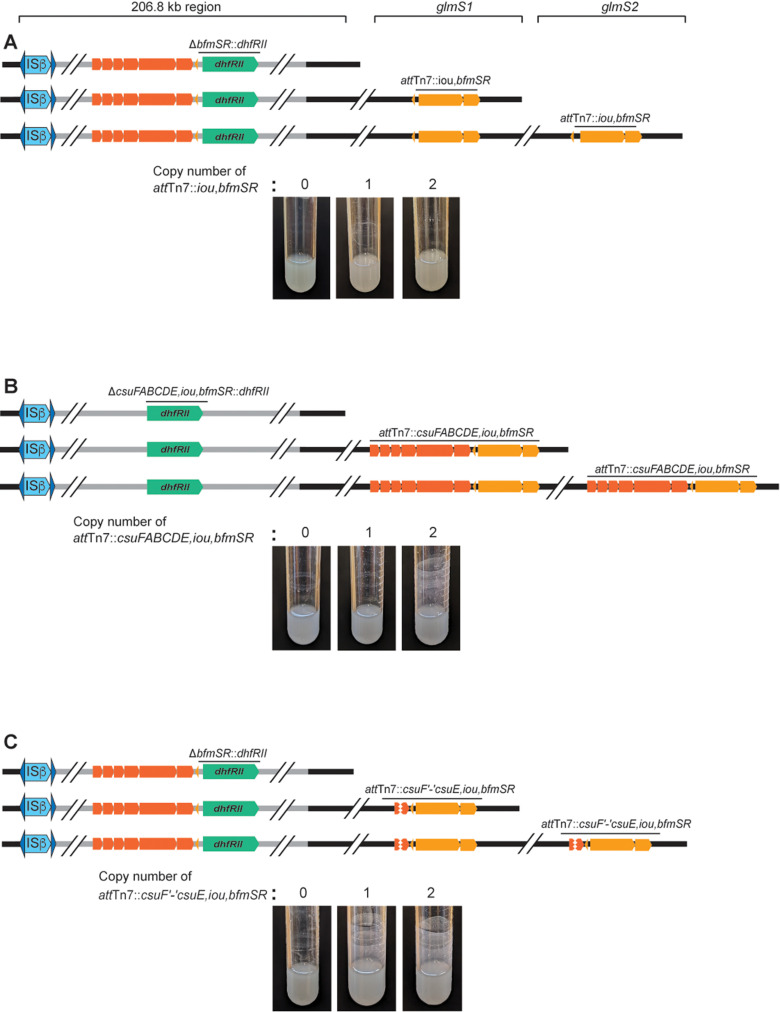
Two copies of *bfmSR* plus the intergenic region 5′ to *csuFABCDE* are sufficient to promote efficient biofilm formation. (A) Top: Schematic of strains with *bfmSR* replaced with *dhfRII* and with *bfmSR* inserted at either one, both, or neither *att*Tn*7* site. Bottom: Images of overnight cultures. (B) Top: Schematic of strains with *csuFABCDE,iou,bfmSR* replaced with *dhfRII* and with *csuFABCDE,iou,bfmSR* inserted at either one, both, or neither *att*Tn*7* site. Bottom: Images of overnight cultures. (C) Top: Schematic of strains with *bfmSR* replaced with *dhfRII* and with *csuF*′*-*′*csuE,iou,bfmSR* inserted at either one, both, or neither *att*Tn*7* site. Bottom: Images of overnight cultures.

Because wild-type *Bt*E264 and *bfmSR* mutants are indistinguishable when grown planktonically in M63 medium, and only *bfmSR*^WT^ (not *bfmSR* mutant) bacteria can form biofilms (either in 24 hours for Dup+ bacteria or four days for Dup– bacteria), we reasoned that planktonic growth in M63 medium is likely a condition in which the BfmSR TCS is inactive, and biofilm growth is a condition in which BfmSR is active. We hypothesized that *bfmSR* is positively autoregulated and that active BfmR (i.e., phosphorylated BfmR (BfmR ~ P)) also activates transcription of the *csuFABCDE* genes, which are also required for biofilm formation. Moreover, we hypothesized that two copies of *bfmSR* causes the level of BfmR ~ P in at least some bacteria growing planktonically in M63 to be above the threshold needed for positive autoregulation, resulting in activation of *csuFABCDE* and *bfmSR* transcription and, consequently, the ability to form biofilms. To test this hypothesis, we cloned *bfmSR* plus the intergenic region between *csuE* and *bfmS* (the putative promoter region for *bfmSR*) into plasmid pUC18-miniTn7-Km and delivered the resulting plasmid (plus the helper plasmid containing the Tn*7* transposase-encoding gene) into a ∆*bfmSR::dhfRII* ∆ISα strain by conjugation. Because *B. thailandensis* E264 contains two *glmS* genes, we could obtain strains containing *bfmSR* (and the 3′ adjacent *nptII* gene, which is not depicted in Fig 5) inserted at either one or both *att*Tn*7* sites (Fig 5A). In contrast to our prediction, the strain containing two copies of *bfmSR* (one at each *att*Tn*7* site) did not form efficient biofilms (Fig 5A). We next cloned the region containing the entire *csuFABCDE,iou,bfmSR* locus, including the intergenic region 5′ to *csuF,* into plasmid pUC18-miniTn7-Km and selected strains in which the *csuFABCDE,iou,bfmSR* (and *nptII*, not depicted in Fig 5*)*-containing cassette was present at one or both *att*Tn7 sites in a Δ*csuFABCDE,iou,bfmSR*::*dhfRII* ∆ISα strain (Fig 5B). The strain containing two copies of the *csuFABCDE,iou,bfmSR* locus formed an efficient biofilm (Fig 5B). It seemed unlikely to us that two copies of *csuFABCDE* are required for efficient biofilm formation. Instead, we hypothesized that the promoter required for activating transcription of *bfmSR* is located 5′ to *csuF*. To test this hypothesis, we constructed a plasmid containing the entire *csuFABCDE,iou,bfmSR* locus but with a large deletion extending from *csuF* to *csuE,* and delivered the plasmid to the ∆*bfmSR::dhfRII* ∆ISα strain by conjugation (Fig 5C). (Note that this strain contains the *csuFABCDE* genes at their native site in the chromosome.) Surprisingly, even the strain with the *csuF*′*-*′*csuE,iou,bfmSR-*containing cassette at only one *att*Tn*7* site formed an efficient biofilm (Fig 5C). These results indicate that two copies of the *csuFABCDE* genes are not required for efficient biofilm formation, and they suggest that a promoter required for activation of both *csuFABCDE* and *bfmSR* expression is located in the intergenic region 5′ to *csuF*, that is, the *csuFABCDE* and *bfmSR* genes are within an operon, the promoter for which is located 5′ to *csuF*. The data also suggest that sequences within the *csuFABCDE* genes somehow negatively influence *bfmSR* expression, possibly by simply increasing the distance, and hence opportunity for transcription termination, between the promoter and *bfmSR*.

### The intergenic region 5′ to *csuF*, but not the region between *csuE* and *bfmS*, contains a promoter that is activated during biofilm growth and when the 208.6 kb region is duplicated, in a *bfmSR*-dependent manner

**Fig 6:**
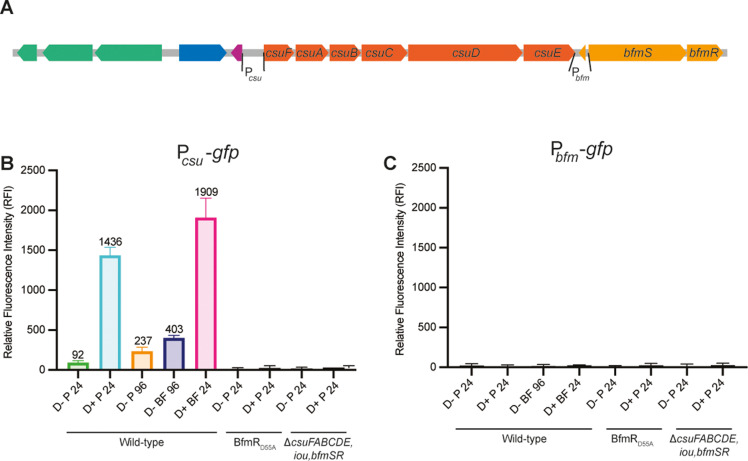
The intergenic region 5′ to *csuF*, but not the region between *csuE* and *bfmS*, contains a promoter that is activated during biofilm growth and when the 208.6 kb region is duplicated, in a *bfmSR*-dependent manner. (A) Schematic of subregion 4 with putative *csu* and *bfm* promoter regions used for construction of promoter-*gfp* fusions indicated. (B) Relative fluorescence intensity of the indicated strains containing the P_*csu*_-*gfp* fusion after 24- or 96-hours growth. P = planktonic, BF = biofilm. (C) Relative fluorescence intensity of the indicated strains containing the P_*bfm*_-*gfp* fusion after 24- or 96- hours growth. P = planktonic, BF = biofilm.

To determine directly if the regions 5′ to *csuF* and *bfmS* contain promoters that are activated when the 208.6 kb region is duplicated and/or during biofilm growth, we cloned each putative promoter region (P_*csu*_ and P_*bfm*_, Fig 6A) 5′ to the *gfp* gene that was codon-optimized for *B. thailandensis* in plasmid pKBM19 (creating pKBM21 and pKBM20, respectively), and delivered the P_*csu*_*-gfp* and P_*bfm*_*-gfp* cassettes to *att*Tn*7* (selecting those with only a single copy at the *glmS1*site) in wild-type and *bfmSR* mutant strains containing either a deletion of ISα (Dup– locked bacteria) or the fragmented *nptII* genes (to select for Dup+ bacteria). For the resulting GFP reporter strains, we grew cultures wherein spent medium was replaced with fresh M63 every 24 hours. Planktonic and biofilm bacteria were collected after 24- or 96-hours growth in M63 medium, washed, fixed, and transferred to a 96-well plate to measure fluorescence intensity.

Planktonically grown cells containing the P_*csu*_*-gfp* fusion produced a low level of fluorescence (92 RFI units) when the 208.6 kb region was present in a single copy after 24 hours growth in M63. By contrast, when the 208.6 kb region was duplicated, the level of fluorescence in bacteria growing planktonically was 1,436 RFI units, a 15.6-fold increase compared to Dup– bacteria (Fig 6B). When Dup+ bacteria containing the P_*csu*_*-gfp* fusion were recovered from the biofilm after 24 hours growth, the level of fluorescence was 1,909 RFI units (Fig 6B). Fluorescence of *bfmS* or *bfmR* mutants containing the P_*csu*_*-gfp* fusion was at background levels under all conditions (Fig 6B), confirming that activation of P_*csu*_ requires active BfmSR.

In Dup– bacteria containing the P_*csu*_*-gfp* fusion grown for 96 hours, the level of fluorescence was 237 RFI units in the planktonic bacteria and 403 RFI units in the bacteria recovered from the biofilm (Fig 6C).

Strains containing the P_*bfm*_*-gfp* fusion produced no fluorescence under any condition, confirming that the region 5′ to *bfmS* does not contain a promoter that is active under any of the growth conditions tested (Fig 6C).

These data show that there is a promoter 5′ to *csuF* and that its expression increases dramatically, in a BfmSR-dependent manner, when the 208.6 kb region is duplicated. These data, together with those shown in Fig 5, strongly suggest that *bfmSR* is positively autoregulated via P_*csu,*_ i.e., that P_*csu*_ is the promoter for the operon that includes *csuFABCDE* and *bfmSR*. P_*csu*_ is also activated during biofilm growth in Dup– bacteria, although not as strongly as in Dup+ bacteria.

### Duplication of *bfmSR* is sufficient to activate P_*csu*_ in a majority of bacteria growing in M63 medium

**Fig 7:**
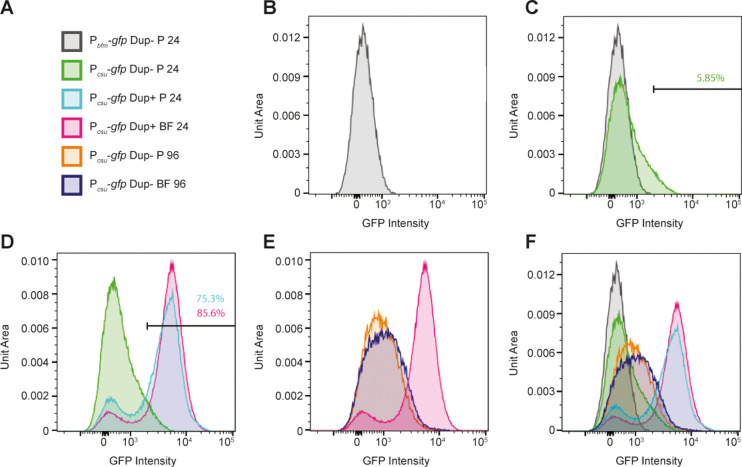
Duplication of *bfmSR* is sufficient for positive autoregulation in a majority of bacteria growing in M63 medium. (A) Legend for the graphs shown in B–F. P = planktonic, BF = biofilm. (B-F) Graphs showing fluorescence of populations of bacteria as indicated by the legend. The level of fluorescence is plotted along the X-axis on a log scale, and the number of bacteria, normalized to unit area under each curve so that different samples can be compared, is plotted along the Y-axis.

The plate reader (data shown in Fig 6) measures total fluorescence in each population of bacteria. To measure fluorescence, and hence P_*csu*_ and P_*bfm*_ expression, in single cells, we analyzed aliquots of the same cultures using flow cytometry. We stained the samples with SYTO 61 to identify cells and to exclude any debris and electronic background. Next, we used forward and side scatter to gate for single bacterial cells and measured the GFP fluorescence of particles contained within these parameters. The level of fluorescence for all bacteria containing the P_*bfm*_-*gfp* fusion was very low (Fig 7B), as expected. For most Dup– planktonic bacteria containing the P_*csu*_*-gfp* fusion, the level of fluorescence was also very low, similar to that of bacteria containing the P_*bfm*_-*gfp* fusion (Fig 7C, green curve). However, the level of fluorescence was higher in ~6% of these bacteria and was quite high in a very small number of bacteria (Fig 7C, right shoulder of green curve). These data are consistent with planktonic growth corresponding to a BfmSR-inactive condition, with a small proportion of bacteria stochastically producing enough BfmR ~ P to activate P_*csu*_, and hence cause positive autoregulation of *bfmSR*.

For Dup+ planktonic bacteria containing the P_*csu*_*-gfp* fusion, two distinct populations were apparent (Fig 7D, cyan curve): 24.7% of the population was essentially non-fluorescent and 75.3% of the population was highly fluorescent, indicating that two copies of *bfmSR* is sufficient for activation of P_*csu*_ – and hence also *bfmSR* – even under BfmSR-inactivating conditions in most bacteria. Dup+ bacteria containing the P_*csu*_*-gfp* fusion collected from the biofilm (Fig 7D, pink graph) had an even higher proportion of highly fluorescent bacteria (85.6%). Dup– bacteria containing the P_*csu*_*-gfp* fusion collected after 96-hours growth displayed a range of fluorescence from low to moderate, with a slight shift towards increased fluorescence in bacteria collected from the biofilm compared to bacteria recovered from the liquid (Fig 7E). Although very few of these bacteria were as fluorescent as Dup+ bacteria.

Together, these data suggest that planktonic growth in M63 medium is a condition in which the BfmSR TCS is inactive, but that a small amount of BfmR ~ P forms stochastically in a small proportion of bacteria under these conditions, and that this level of BfmR ~ P is sufficient to activate P_*csu*_, and hence cause positive autoregulation of *bfmSR*, which increases the amount of BfmR ~ P in those cells even further. The data suggest further that simply doubling the copy number of *bfmSR* causes the level of BfmR ~ P that forms stochastically to be above the threshold required for activation of P_*csu*_ in a majority (~75%) of bacteria.

## Discussion

The first goal of this study was to identify the gene(s) within the 208.6 kb region of *Bt*E264 that, when duplicated, causes efficient biofilm formation. Our data indicate that *bfmSR*, which encode an unorthodox two component regulatory system (TCS), are the responsible genes. We showed that both *bfmSR* and *csuFABCDE* (encoding an archaic chaperone usher pathway (CUP) pilus system) are required for biofilm formation, but only duplicate copies of *bfmSR*, not *csuFABCDE*, are required to cause efficient biofilm formation. Our data also indicate that the intergenic region 5′ to *bfmSR* (between *csuE* and *bfmS*) does not contain a promoter that is active under any of the conditions tested and that, instead, expression of *bfmSR* appears to be driven by a promoter located 5′ to *csuF*. We did not rule out the unlikely possibility that there is an additional promoter within the *csuFABCDE* genes. If so, its activity would mimic that of P_*csu*_, at least under the conditions we investigated, since P_*csu*_ activity correlates with biofilm formation, which requires BfmSR activity. The more likely scenario is that *csuFABCDE* and *bfmSR* are members of an operon that is transcribed from P_*csu*_, and that *bfmSR* is, therefore, positively autoregulated.

One of the most common mechanisms used by bacteria to adapt to ever-changing environmental conditions is the TCS. Composed generally of a histidine kinase that senses environmental cues and, in response, phosphorylates a response regulator that affects a change in behavior (usually by activating and/or repressing gene expression), TCSs allow all members of a population to adapt, maximizing their fitness to a specific environmental condition [16]. In Dup– bacteria, P_*csu*_ expression, which requires BfmSR activity, is very low in planktonic bacteria and substantially higher in biofilm bacteria. Together with the fact that although wild-type and ∆*bfmSR* bacteria grow similarly when growing planktonically, only *bfmSR*^WT^ bacteria form biofilms, these data strongly suggest that the BfmSR system is inactive during planktonic growth and active during biofilm growth.

Biofilm formation requires adherence of the bacteria to a biotic or abiotic surface, as well as to each other. This adherence is often mediated by hair-like surface appendages known as fimbriae or pili. In Gram-negative bacteria, adhesive pili are frequently assembled by the chaperone-usher pathway (CUP), of which there are three groups: classical, alternative, and archaic [17]. Archaic CUP pili are more widespread than classical and alternative CUP pili [17], but there is much less known about them. They have recently been shown to form ultrathin, superelastic, zigzag structures that may facilitate adherence under dynamic conditions [18,19], and they are required for biofilm formation in *Pseudomonas aeruginosa* and *Acintetobacter baumannii* [10,11]*.* In *B. pseudomallei*, *csuFABCDE* homologs were shown to be more highly expressed in biofilm overproducing strains compared to strains that produce less biofilm [13]. We showed previously that a plasmid insertion into *csuD* in *Bt*E264 abrogated biofilm formation, however that insertion likely had polar effects on the downstream *bfmSR* genes [20]. This work, therefore, is the first to show the requirement of the *csuFABCDE* genes in biofilm formation in *Burkholderia*, and we hypothesize that the role of the CUP pili is to allow the bacteria to adhere to the walls of the test tube as well as to each other.

Together, these data indicate that BfmSR controls a deterministic response, with all bacteria in the population behaving the same. When *Bt*E264 containing only a single copy of the 208.6 kb region is growing in liquid medium, BfmSR is inactive, the *csuFABCDE* genes are expressed at a minimal level (likely too low for any pili to be produced), and the bacteria grow planktonically. Under biofilm conditions, which occurs for unknown reasons after several days growth in M63 medium at the air-liquid interface of the culture, BfmSR becomes active, inducing expression of *csuFABCDE* and *bfmSR* (and likely other genes), resulting in an amplified response and the production of pili, that, we propose, mediate adherence of the bacteria to the abiotic surface and to each other.

Our second goal was to determine the mechanism by which duplicate copies of the identified gene(s) causes efficient biofilm formation. Because biofilm formation requires active BfmR, i.e., BfmR ~ P, (based on the fact that the BfmR_D55A_ mutant cannot activate P_*csu*_ or form biofilms), duplication of *bfmSR* must somehow cause at least some bacteria to activate BfmSR during the first 24 hours of growth in liquid medium, a BfmSR-inactivating condition. Although TCSs are often considered on/off switches, expression of the genes encoding the TCS cannot be zero under ‘off’ conditions or there would be no TCS to sense and respond to ‘on’ conditions. Moreover, consistent with any enzymatic reaction, the amount of phosphorylated response regulator protein in off conditions will not be zero; a small, but finite, level of response regulator will be phosphorylated stochastically. This low level of phosphorylated response regulator protein is typically below the threshold required to activate or repress gene expression, and therefore the vast majority of bacteria in the population will display the ‘TCS off’ phenotype. However, in systems that are positively autoregulated, bacteria in which the amount of phosphorylated response regulator is sufficient to activate transcription of the TCS-encoding genes will amplify the system, often leading to bacteria that are phenotypically ‘on’, even under TCS off conditions. The proportion of bacteria that are ‘on’, and therefore maladapted for the specific environment, is typically very low. For BfmSR, the data shown in Fig 7 indicate that the proportion of planktonically growing bacteria with high levels of P_*csu*_ expression in Dup– bacteria is ~ 6% (a higher number than we would have predicted), suggesting that BfmR is stochastically phosphorylated in ~6% of bacteria growing in BfmSR ‘off’ conditions. For Dup+ bacteria, the proportion of bacteria with high levels of P_*csu*_ expression was ~ 75%, indicating that simply doubling the copy number of BfmS and BfmR is sufficient to cause stochastic activation of BfmR in a majority of cells. These data suggest that the amount of BfmR ~ P required to activate P_*csu*_ is very low, perhaps just below the average amount of BfmR ~ P present in most Dup– cells growing planktonically under the growth conditions used in our experiments. Doubling the amount of BfmS and BfmR appears to increase the amount of BfmR ~ P in most cells to a level sufficient to activate P_*csu*_. Although many details of this hypothesis remain untested, our data suggest that the mechanism underlying duplication-dependent efficient biofilm formation is simply that the amount of BfmR ~ P required to activate P_*csu*_ is just below the amount present in most Dup– bacteria growing planktonically, and that duplication of *bfmSR* causes that amount to be over the threshold required to activate P_*csu*_ in a majority (~75%) of bacteria.

How does this phenomenon constitute a bet-hedging strategy? The near ubiquity of TCSs in bacteria and some eukaryotes [21] is evidence of the success of deterministic control of gene expression (or other behavioral changes) in bacterial and some fungal populations. However, when facing unrecognizable stimuli, or when conditions fluctuate too rapidly for signal transduction systems to mount an appropriate response, such deterministic responses may be inadequate. In these cases, bet-hedging strategies, wherein a subpopulation stochastically exhibits a phenotype that is maladapted for the current condition but essential in another, may allow population survival [22]. In *Bt*E264, stochastic RecA-dependent duplication of the 208.6 kb region results in a sub-population of bacteria with a BfmSR system that is active under BfmSR-inactivating conditions. Our previous work estimates the number of Dup+ bacteria in *Bt*E264 cultures growing in M63 liquid medium to be about 1 in 10,000 [7]. Although these bacteria are at a disadvantage during growth in liquid, their readiness to form biofilms provides a strong selective advantage should conditions requiring adherence to a solid surface appear suddenly – such as when the liquid phase of a culture, and the planktonically-growing bacteria therein, is removed from the culture tube. In nature, we speculate that Dup+ bacteria may have an advantage when soil drains rapidly and only bacteria attached to plant roots or other solid surfaces remain.

Many questions remain unanswered about this system, a prominent one being the signal that is sensed by BfmS. While the signal sensed by bacterial histidine kinases is known for only a few, the lack of obvious sensing domains makes predicting the signals to which BfmS responds even more difficult. However, *bfmSR* homologs have been identified in the *Burkholderia pseudomallei* [23,24], and there is evidence that *bfmSR* expression and BfmSR activity in this organism may increase under iron-limiting conditions [12]. It is possible that after sustained growth in M63 medium, and maybe more so for bacteria accumulating on the walls of the test tube, iron and/or other nutrients are depleted, shifting the conditions towards those in which BfmS is active. We will investigate this possibility in the future.

Comparisons between *B. thailandensis* and *B. pseudomallei* yielded additional insight. Although the amino acid sequences of BfmS in *Bt*E264 and *B. pseudomallei* K96243 are 90% identical and 92% similar, SMART predicts a transmembrane domain at the N-terminus of K96243 BfmS but not for *Bt*E264 BfmS. As most sensor kinases are cytoplasmic membrane proteins, it is likely that *Bt*E264 BfmS contains a trans-membrane domain that was not identified by SMART, but biochemical experiments will be required to determine if BfmS localization in this organism. It is also noteworthy that although the intergenic sequences between *csuFABCDE* and *bfmS* in *B. thailandensis* and *B. pseudomallei* are nearly identical, a single additional nucleotide in the *B. pseudomallei* sequence abrogates the prediction of an ORF in this region. This information, together with the fact that *iou* is not required for either efficient or inefficient biofilm formation or for the selective advantage conferred by duplicating the 208.6 kb region during biofilm growth in *Bt*E264, suggests that *iou* may not encode a functional protein in either organism.

Genetic linkage between genes encoding regulatory proteins and the genes they control is common, and it is also common for TCSs to (also) regulate unlinked genes. While the full BfmSR regulon is currently unknown, genes in addition to *csuFABCDE*, including those predicted to encode an exopolysaccharide, are required for biofilm formation [20]. In a previous report from our group, we compared the transcriptomes of wild-type *Bt*E264 with those of strains in which expression of the *bcpAOIB* genes (encoding a contact-dependent inhibition system) was driven by the constitutive S12 promoter or that produced a catalytically inactive BcpA protein [20]. We now know that these strains were predominantly Dup+ and Dup–, respectively. Consistent with our current data, transcript abundance for the *csuFABCDE* and *bfmSR* genes was dramatically increased in the strain with P_S12_ driving *bcpAIOB* compared to the BcpA mutant, as were many others, including those predicted to encode an exopolysaccharide. However, because the 208.6 kb region contains several genes that are predicted to encode transcription regulators, additional experiments will be required to determine if any of the differentially-expressed genes identified in the previous study are controlled by BfmSR.

In the current study, we focused on efficient biofilm formation, but other duplication-dependent phenotypes, such as Congo red binding and the production of a gold-brown pigment, exist [6,7]. We do not know, at this point, what genes are responsible for those phenotypes or whether they are regulated by BfmSR. Indeed, these other phenotypes could require duplication of a different subregion within the 208.6 kb region. If these other duplication-dependent phenotypes are beneficial, it could explain the selective advantage for strains with ISα and ISβ in their current locations. We showed in this work that duplication of only the *csuFABCDE,iou,bfmSR* genes is sufficient for efficient biofilms to form and for the selective advantage conferred by duplication during biofilm growth. Since duplication of a smaller region would provide less homology for recombination-mediated loss of the duplication, it would seem to be more advantageous for ISα and ISβ to be closer together. Are their current locations just chance, or is duplication of other genes within the 208.6 kb region advantageous under conditions that we have not yet explored? Continued investigation will be required to understand the depth and breadth of the stochastic, IS-mediated bet-hedging strategy in *Bt*E264.

Interplay between TCS signaling and phase variation is not uncommon. In many cases, phase variation removes one or more genes from the deterministic control of the TCS without altering the entire regulon. For example, phase variation-mediated mutation of genes encoding *Helicobacter pylori* adhesins and *Neisseria meningitidis* hemoglobin receptors, which are regulated by TCSs that control many genes required for infection, prevents production of these immunostimulatory proteins, thereby allowing the bacteria to evade the host immune system [25–29]. In other cases, phase variation toggles on or off production of the TCSs themselves, leading to two distinct subpopulations; phase variation-ON subpopulations that produce the TCS and are able to sense-and-respond to the recognized stimulus, and phase variation-OFF subpopulations that are effectively blind to the activating stimulus [30–35]. DNA duplication-mediated activation of BfmSR represents another form of interplay between TCS signaling and phase variation, further highlighting the positive impact that transposable elements can have on the evolution of bacterial populations.

## Materials and methods

### Plasmids, strains, and Bacterial culture conditions

Plasmids and strains used in this study are listed in Tables 1 and 2, respectively. *Bt*E264 is an environmental isolate [1]. Plasmids were maintained in *E. coli* DH5α. For insertion at the *att*Tn*7* sites or Flp-mediated FRT recombination, plasmids were introduced into *Bt*E264 by conjugation with *E. coli* donor strain RHO3 [38]. *Bt*E264 and *E. coli* strains were grown overnight (24 hours) with aeration at 37°C (unless indicated) in low-salt Luria-Bertani (LSLB, 0.5% NaCl). Antibiotics and supplements were added to cultures at the following concentrations: 50 μg/mL X-Gluc (5-bromo-4-chloro-3-indoxyl-beta-D-glucuronide), 200 μg/mL 2,6-diaminopimelic acid (DAP), 0.2% (wt/vol) rhamnose, 500 μg/mL (for *Bt*E264) or 50 μg/mL (for *E. coli*) kanamycin (Km), 100 μg/mL (for *Bt*E264) or 50 μg/mL (for *E. coli*) trimethoprim (Tmp), 50 μg/mL (for *Bt*E264) or 10 μg/mL (for *E. coli*) tetracycline (Tc), 100 μg/mL ampicillin (Ap), 200 μg/mL zeocin (Zeo), or 30 μg/mL (for *Bt*E264) chloramphenicol (Cm) as appropriate. Because trimethoprim exposure alters gene expression in *Burkholderia thailandensis*, trimethoprim was only used to select for stable mutations that do not require continuous selection, and experiments were conducted without trimethoprim [39]. When indicated, *Bt*E264 was cultured in M63 minimal medium (110 mM KH_2_PO_4_, 200 mM K_2_HPO_4_, 75 mM (NH_4_)_2_SO_4_, 16 nM FeSO_4_) supplemented with 1 mM MgSO_4_, 0.2% glucose, 0.4% glycerol, and 0.01% casamino acids.

**Table 1:** Plasmids used in this study.

Plasmid	Information	Reference
pJET1.2	Common cloning vector. Ap^R^	Thermo Fisher
pUC18-mini-Tn7	Plasmid for delivering DNA fragments to *att*Tn*7* sites. Ap^R^, Km^R^	[36]
pTNS3	Helper plasmid carrying TN-n7 transposase for Tn7 site insertion of pUC18-mini-Tn7 cargo	[36]
pSM112	Suicide plasmid for *Burkholderia* with chloramphenicol resistance. Cm^R^	[37]
pLL79	pUC18-mini-Tn7 containing 500 bp corresponding to the junction between ISβ and subregion 1, with an I-*Sce*I restriction site at the 3’ end. Ap^R^, Km^R^	This study
pLL89	pSM112 containing 500 bp corresponding to the junction between subregions 1 and 2, with an I-*Sce*I restriction site at the 3’ end. Cm^R^	This study
pLL80	pUC18-mini-Tn7 containing 500 bp corresponding to the junction between subregions 1 and 2, with an I-*Sce*I restriction site at the 3’ end. Ap^R^, Zeo^R^	This study
pLL90	pSM112 containing 500 bp corresponding to the junction between subregions 2 and 3, with an I-*Sce*I restriction site at the 3’ end. Cm^R^	This study
pLL81	pUC18-mini-Tn7 containing 500 bp corresponding to the junction between subregions 2 and 3, with an I-*Sce*I restriction site at the 3’ end. Ap^R^, Km^R^	This study
pLL91	pSM112 containing 500 bp corresponding to the junction between subregions 3 and 4, with an I-*Sce*I restriction site at the 3’ end. Cm^R^	This study
pLL82	pUC18-mini-Tn7containing 500 bp corresponding to the junction between subregions 3 and 4, with an I-*Sce*I restriction site at the 3’ end. Ap^R^, Zeo^R^	This study
pLL92	pSM112 containing 500 bp corresponding to the junction between subregions 4 and 5, with an I-*Sce*I restriction site at the 3’ end. Cm^R^	This study
pLL83	pUC18-mini-Tn7 containing 500 corresponding to the junction between subregions 4 and 5, with an I-*Sce*I restriction site at the 3’ end. Ap^R^, Km^R^	This study
pLL93	pSM112 containing 500 bp corresponding to the junction between subregions 5 and 6, with an I-*Sce*I restriction site at the 3’ end. Cm^R^	This study
pLL84	pUC18-mini-Tn7containing 500 bp corresponding to the junction between subregions 5 and 6, with an I-*Sce*I restriction site at the 3’ end. Ap^R^, Zeo^R^	This study
pLL94	pSM112 containing 500 bp corresponding to the junction between subregions 6 and 7, with an I-*Sce*I restriction site at the 3’ end. Cm^R^	This study
pLL85	pUC18-mini-Tn7 containing 500 bp corresponding to the junction between subregions 6 and 7, with an I-*Sce*I restriction site at the 3’ end. Ap^R^, Km^R^	This study
pLL95	pSM112 containing 500 bp corresponding to the junction between subregions 7 and 8, with an I-*Sce*I restriction site at the 3’ end. Cm^R^	This study
pLL86	pUC18-mini-Tn7 containing 500 bp corresponding to the junction between subregions 7 and 8, with an I-*Sce*I restriction site at the 3’ end. Ap^R^, Zeo^R^	This study
pLL96	pSM112 containing 500 bp corresponding to the junction between subregions 8 and 9, with an I-*Sce*I restriction site at the 3’ end. Cm^R^	This study
pLL87	pUC18-mini-Tn7 containing 500 bp corresponding to the junction between subregions 8 and 9, with an I-*Sce*I restriction site at the 3’ end. Ap^R^, Km^R^	This study
pLL97	pSM112 containing 500 bp corresponding to the junction between subregion 9 and 3’ to subregion 9 and ISα, with an I-*Sce*I restriction site at the 3’ end. Cm^R^	This study
pLL98	pUC18-mini-Tn7 containing subregion 1. Ap^R^, Km^R^	This study
pLL99	pUC18-mini-Tn7 containing subregion 2. Ap^R^, Km^R^	This study
pLL100	pUC18-mini-Tn7 containing subregion 3. Ap^R^, Km^R^	This study
pLL101	pUC18-mini-Tn7 containing subregion 4. Ap^R^, Km^R^	This study
pLL102	pUC18-mini-Tn7 containing subregion 5. Ap^R^, Km^R^	This study
pLL103	pUC18-mini-Tn7 containing subregion 6. Ap^R^, Km^R^	This study
pLL104	pUC18-mini-Tn7 containing subregion 7. Ap^R^, Km^R^	This study
pLL105	pUC18-mini-Tn7 containing subregion 8. Ap^R^, Km^R^	This study
pLL106	pUC18-mini-Tn7 containing subregion 9. Ap^R^, Km^R^	This study
pLL111	pUC18-mini-Tn7 containing sub-subregion 4.1. Ap^R^, Km^R^	This study
pLL110	pUC18-mini-Tn7 containing sub-subregion 4.2. Ap^R^, Km^R^	This study
pLL109	pUC18-mini-Tn7 containing sub-subregion 4.3. Ap^R^, Km^R^	This study
pLL108	pUC18-mini-Tn7 containing sub-subregion 4.4. Ap^R^, Km^R^	This study
pLL107	pUC18-mini-Tn7 containing sub-subregion 4.5. Ap^R^, Km^R^	This study
pLL120	pUC18-mini-Tn7 containing sub-subregions 4.4 and 4.5. Ap^R^, Km^R^	This study
pLL113	pJET1.2 containing *dhfRII* flanked by 500 bp 5’ and 3’ to *csuFABCDE.* Ap^R^, Tmp^R^	This study
pLL172	pUC18-mini-Tn7 containing *dhfRII* flanked by 500 bp 5’ and 3’ to *iou*. Ap^R^, Tmp^R^	This study
pLL112	pJET1.2 containing *dhfRII* flanked by 500 bp 5’ and 3’ to *bfmSR*. Ap^R^, Tmp^R^	This study
pLL119	pJET1.2 containing *dhfRII* flanked by 500’ bp 5’ and 3’ to *csuFABCDE,iou,bfmSR*. Ap^R^, Tmp^R^	This study
pLL162	pJET1.2 containing *ble* and *gusA*_1-1454_ (*gusA*’) flanked by 500 bp corresponding to sequences immediately 5’ to *csuF*. Ap^R^, Zeo^R^	This study
pLL161	pJET1.2 containing *tet* and *gusA*118–1691 (‘*gusA*) flanked by 500 bp corresponding to sequences immediately 3’ to *bfmR*. Ap^R^, Tc^R^	This study
pEXKm5	*sacB*-based allelic exchange plasmid. Km^R^	[38]
pLL144	pEXKm5 containing sequences to delete the entire *bfmS* gene. Km^R^	This study
pLL145	pEXKm5 containing sequences to delete the entire *bfmR* gene. Km^R^	This study
pLL143	pEXKm5 containing a 487 bp DNA fragment with codon 55 (CAG) replaced with the codon for alanine (GCT). Km^R^	This study
pLL177	pUC18-mini-Tn7 containing *csuF*’*-*’*csuE,iou,bfmSR*. Ap^R^, Km^R^	This study
pKBM19	pUC-18-mini-Tn7 derivative with *nptII* replaced with *cat* and a codon optimized *gfp* gene driven by P_S12_. Ap^R^, Cm^R^	This study
pKBM21	pKBM19 derivative with P_S12_ replaced with 570 bp 5’ to *csuF* (P_*csu*_)*.* Ap^R^, Cm^R^	This study
pKBM20	pKBM19 derivative with P_S12_ replaced with 527 bp 5’ to *bfmSR* (P_*bfm*_). Ap^R^, Cm^R^	This study

**Table 2:** Strains used in this study.

Strain	Information	Reference
E264	Wild-type *B. thailandensis* environmental isolate	[1]
ΔISβ::*nptII*	ISβ *orfAB* and inverted repeat sequences replaced by *nptII*	[4]
ΔISβ	ΔISβ::*nptII* with *nptII* removed via Flp-RFT recombination	This study
Strain for isolating subregion 1	ΔISβ with pLL79 and pLL89 integrated into the chromosome	This study
Strain for isolating subregion 2	ΔISβ with pLL80 and pLL90 integrated into the chromosome	This study
Strain for isolating subregion 3	ΔISβ with pLL81 and pLL91 integrated into the chromosome	This study
Strain for isolating subregion 4	ΔISβ with pLL82 and pLL92 integrated into the chromosome	This study
Strain for isolating subregion 5	ΔISβ with pLL83 and pLL93 integrated into the chromosome	This study
Strain for isolating subregion 6	ΔISβ with pLL84 and pLL94 integrated into the chromosome	This study
Strain for isolating subregion 7	ΔISβ with pLL85 and pLL95 integrated into the chromosome	This study
Strain for isolating subregion 8	ΔISβ with pLL86 and pLL96 integrated into the chromosome	This study
Strain for isolating subregion 9	ΔISβ with pLL87 and pLL97 integrated into the chromosome	This study
Strain with duplicate copies of subregion 1	ΔISβ with pLL98 integrated into the chromosome at the native site	This study
Strain with duplicate copies of subregion 2	ΔISβ with pLL99 integrated into the chromosome at the native site	This study
Strain with duplicate copies of subregion 3	ΔISβ with pLL100 integrated into the chromosome at the native site	This study
Strain with duplicate copies of subregion 4	ΔISβ with pLL101 integrated into the chromosome at the native site	This study
Strain with duplicate copies of subregion 5	ΔISβ with pLL102 integrated into the chromosome at the native site	This study
Strain with duplicate copies of subregion 6	ΔISβ with pLL103 integrated into the chromosome at the native site	This study
Strain with duplicate copies of subregion 7	ΔISβ with pLL104 integrated into the chromosome at the native site	This study
Strain with duplicate copies of subregion 8	ΔISβ with pLL105 integrated into the chromosome at the native site	This study
Strain with duplicate copies of subregion 9	ΔISβ with pLL106 integrated into the chromosome at the native site	This study
Strain with duplicate copies of sub-subregion 4.1	ΔISβ with pLL111 integrated into the chromosome at the native site	This study
Strain with duplicate copies of sub-subregion 4.2	ΔISβ with pLL110 integrated into the chromosome at the native site	This study
Strain with duplicate copies of sub-subregion 4.3	ΔISβ with pLL109 integrated into the chromosome at the native site	This study
Strain with duplicate copies of sub-subregion 4.4	ΔISβ with pLL108 integrated into the chromosome at the native site	This study
Strain with duplicate copies of sub-subregion 4.5	ΔISβ with pLL107 integrated into the chromosome at the native site	This study
Strain with duplicate copies of sub-subregion 4.4 and sub-subregion 4.5	ΔISβ with pLL120 integrated into the chromosome at the native site	This study
Fragmented *nptII* reporter strain	Described previously	[4]
Fragmented *nptII* reporter strain Δ*csuFABCDE*::*dhfRII*	The fragmented *nptII* reporter strain with *csuFABCDE* replaced with *dhfRII* using pLL113	This study
Fragmented *nptII* reporter strain Δ*iou*::*dhfRII*	The fragmented *nptII* reporter strain with *iou* replaced with *dhfRII* using pLL172	This study
Fragmented *nptII* reporter strain Δ*bfmSR*::*dhfRII*	The fragmented *nptII* reporter strain with *bfmSR* replaced with *dhfRII* using pLL112	This study
Fragmented *nptII* reporter strain Δ*csuFABCDE*::*dhfRII*	The fragmented *nptII* reporter strain with *csuFABCDE* replaced with *dhfRII* using pLL113	This study
Fragmented *nptII* reporter strain Δ*csuFABCDE,iou,bfmSR*::*dhfRII*	The fragmented *nptII* reporter strain with *csuFABCDE,iou,bfmSR* replaced with *dhfRII* using pLL119	This study
Fragmented *gusA* reporter strain	Described previously	[4]
Fragmented *gusA* reporter strain Δ*csuFABCDE*::*dhfRII*	The fragmented *gusA* reporter strain with *csuFABCDE* replaced with *dhfRII* using pLL113	This study
Fragmented *gusA* reporter strain Δ*iou*::*dhfRII*	The fragmented *gusA* reporter strain with *iou* replaced with *dhfRII* using pLL172	This study
Fragmented *gusA* reporter strain Δ*bfmSR*::*dhfRII*	The fragmented *gusA* reporter strain with *bfmSR* replaced with *dhfRII* using pLL112	This study
Fragmented *gusA* reporter strain Δ*csuFABCDE,iou,bfmSR*::*dhfRII*	The fragmented *gusA* reporter strain with *csuFABCDE,iou,bfmSR* replaced with *dhfRII* using pLL119	This study
Reduced fragmented *gusA* reporter strain	ΔISβ with pLL162 and pLL161 integrated into the chromosome to flank *csuFABCDE* and *bfmSR*	This study
Fragmented *nptII* reporter strain Δ*bfmS*	The fragmented *nptII* reporter strain with *bfmS* deleted through allelic exchange with pLL144	This study
Fragmented *nptII* reporter strain Δ*bfmR*	The fragmented *nptII* reporter strain with *bfmR* deleted through allelic exchange with pLL145	This study
Fragmented *nptII* reporter strain BfmR_D55A_	The fragmented *nptII* reporter strain with a D55A mutation introduced in *bfmR* through allelic exchange with pLL143	This study
ΔISα::*nptII*	ISα *orfAB* and inverted repeat sequences replaced by *nptII*	[4]
ΔISα	ΔISα::*nptII* with *nptII* removed via Flp-RFT recombination	This study
ΔISα Δ*bfmSR*::*dhfRII*	ΔISα with *bfmSR* replaced with *dhfRII* using pLL112	This study
ΔISα Δ*bfmSR*::*dhfRII att*Tn7::*iou,bfmSR* x1	ΔISα Δ*bfmSR*::*dhfRII* with pLL107 inserted at *glmS-1*	This study
ΔISα Δ*bfmSR*::*dhfRII att*Tn7::*iou,bfmSR* x2	ΔISα Δ*bfmSR*::*dhfRII* with pLL107 inserted at *glmS-1*and *glmS-2*	This study
ΔISα Δ*csuFABCDE,iou,bfmSR*::*dhfRII*	ΔISα with *csuFABCDE,iou,bfmSR* replaced with *dhfRII* using pLL119	This study
ΔISα Δ*csuFABCDE,iou,bfmSR*::*dhfRII att*Tn7::*csuFABCDE,iou,bfmSR* x1	ΔISα Δ*csuFABCDE,iou,bfmSR*::*dhfRII* with pLL120 inserted at *glmS-1*	This study
ΔISα Δ*csuFABCDE,iou,bfmSR*::*dhfRII att*Tn7::*csuFABCDE,iou,bfmSR* x2	ΔISα Δ*csuFABCDE,iou,bfmSR*::*dhfRII* with pLL120 inserted at *glmS-1* and *glmS-2*	This study
ΔISα Δ*bfmSR*::*dhfRII att*Tn7::*csuF*’*-*’*csuE,iou,bfmSR* x1	ΔISα Δ*bfmSR*::*dhfRII* with pLL177 inserted at *glmS-1*	This study
ΔISα Δ*bfmSR*::*dhfRII att*Tn7::*csuF*’*-*’*csuE,iou,bfmSR* x2	ΔISα Δ*bfmSR*::*dhfRII* with pLL177 inserted at *glmS-1*and *glmS-2*	This study
ΔISα P_*csu*_-*gfp*	ΔISα with pKBM21 inserted at *glmS-1*	This study
Fragmented *nptII* reporter strain P_*csu*_-*gfp*	Fragmented *nptII* reporter strain with pKBM21 inserted at *glmS-1*	This study
ΔISα BfmR_D55A_	ΔISα with a D55A mutation introduced in *bfmR* through allelic exchange with pLL143	This study
ΔISα BfmR_D55A_ P_*csu*_-*gfp*	ΔISα BfmR_D55A_ with pKBM21 inserted at *glmS-1*	This study
Fragmented *nptII* reporter strain BfmR_D55A_ P_*apl*_-*gfp*	Fragmented *nptII* reporter strain BfmR_D55A_ with pKBM21 inserted at *glmS-1*	This study
ΔISα Δ*csuFABCDE,iou,bfmSR*::*dhfRII* P_*apl*_-*gfp*	ΔISα Δ*csuFABCDE,iou,bfmSR*::*dhfRII* with pKBM21 inserted at *glmS-1*	This study
Fragmented *nptII* reporter strain Δ*csuFABCDE,iou,bfmSR*::*dhfRII* P_*apl*_-*gfp*	Fragmented *nptII* reporter strain Δ*csuFABCDE,iou,bfmSR*::*dhfRII* with pKBM21 inserted at *glmS-1*	This study
ΔISα P_*bfm*_-*gfp*	ΔISα with pKBM20 inserted at *glmS-1*	This study
Fragmented *nptII* reporter strain P_*bfm*_-*gfp*	Fragmented *nptII* reporter strain with pKBM20 inserted at *glmS-1*	This study
ΔISα BfmR_D55A_ P_*bfm*_-*gfp*	ΔISα BfmR_D55A_ with pKBM20 inserted at *glmS-1*	This study
Fragmented *nptII* reporter strain BfmR_D55A_ P_*bfm*_-*gfp*	Fragmented *nptII* reporter strain BfmR_D55A_ with pKBM20 inserted at *glmS-1*	This study
ΔISα Δ*csuFABCDE,iou,bfmSR*::*dhfRII* P_*bub*_-*gfp*	ΔISα Δ*csuFABCDE,iou,bfmSR*::*dhfRII* with pKBM20 inserted at *glmS-1*	This study
Fragmented *nptII* reporter strain Δ*csuFABCDE,iou,bfmSR*::*dhfRII* P_*bub*_-*gfp*	Fragmented *nptII* reporter strain Δ*csuFABCDE,iou,bfmSR*::*dhfRII* with pKBM20 inserted at *glmS-1*	This study

### Mutant construction techniques

#### Natural transformation.

Linearized plasmids containing an antibiotic resistance-encoding gene flanked by ~500 bp sequences with homology to genomic regions of interest were introduced to *Bt*E264 following previously described protocols [40]. Transformants were isolated on LSLB-supplemented with the appropriate antibiotic and verified through PCR analysis.

#### Allelic exchange.

Markerless mutations were constructed through allelic exchange with *sacB* counterselection. Plasmids with a pEXKm5 backbone were constructed to contain mutant DNA sequences with homology to the chromosome and subsequently introduced into *E. coli* RHO3. RHO3 were then mated with a *Bt*E264 strain of interest and transformants were isolated on LSLB-supplemented with the appropriate antibiotic. Allelic exchange was conducted using previously established protocols [38].

#### Plasmid integration.

Plasmids containing sequences of interest with homology to the genome were introduced into RHO3 and were subsequently mated into *Bt*E264, in which, the plasmid backbone could not replicate. Single homologous recombination between the chromosome and the introduced plasmid would integrate plasmid DNA into the chromosome. Transformants were isolated on LSLB-supplemented with the appropriate antibiotic.

#### Flp-FRT recombination.

Flp-mediated FRT recombination was used to excise antibiotic cassettes flanked by FRT sequences. Recombination was conducted according to previously described protocols [41].

#### attTn7 site insertion.

Introduction of DNA sequences to one or both *att*Tn7 sites was conducted according to previously described protocols [36] through mating between *Bt*E264, *E. coli* RHO3 carrying the Tn7 transposase-containing plasmid pTNS3, and *E. coli* RHO3 carrying a plasmid with DNA sequences for insertion. Mutants were confirmed with PCR analysis.

### PCR analysis

Colony PCR was conducted using either GoTaq or OneTaq DNA polymerases in reactions with 4% DMSO. PCR generation of DNA sequences for cloning was accomplished using the Q5 High-fidelity DNA polymerase with Q5 High GC enhancer.

### Plasmid rescue

A pair of plasmids bearing 500 bp sequences homologous to the boundaries of each subregion were separately introduced into the chromosome. Cells with integrated plasmids were isolated with zeocin or chloramphenicol and verified through colony PCR. Genomic DNA was isolated from each of the nine resulting strains using the Wizard Genomic DNA Purification kit.

Genomic DNA was digested generating fragments of the subregion of interest. Reactions were conducted overnight at 37˚ C in a 100 μL reaction with 2 μg of gDNA and 100 units of I-SceI. Reactions were then heat inactivated at 65˚ C for 20 min. Samples were then ligated to generate large plasmids carrying a subregion of interest. The ligation reaction was carried out at 16˚ C overnight with 86 μL of the digested gDNA, 4 μL of T4 DNA ligase and 10 μL of 10x T4 buffer.

50 μL of the ligation product was then transformed into NEB 10-Beta chemical competent cells and colonies were isolated with ampicillin.

### Efficient biofilm growth

Overnight cultures were started from a single colony and grown for 18 hours in LSLB. The OD_600_ of each sample was measured, confirming that mutant strains had no growth rate defect, and diluted to an OD_600_ of 0.2 in 2 mL of M63 within 14 mL polystyrene test tubes (Falcon, Product Number 352057). Cultures were then grown on a rotator at 30˚ up from horizontal, 41 RPM, and 37˚ C for 24 hours before imaging. Experiments were conducted in triplicate at minimum and images presented are representative of how each strain appeared for each replicate.

### Biofilm selection

Biofilm selection through serial passaging was conducted as described previously [7]. Populations were initially composed of 2% Dup+ bacteria.

To determine the proportion of Dup+ cells within the biofilm population at each day, the biofilm was sampled by scraping a sterile loop along the walls of the test tube at the air-liquid interface. We then streaked for single colonies on LSLB plates supplemented with 50 μg/mL X-Gluc. Plates were incubated at 37°C overnight to form visible colonies, and at room temperature for 3–4 days to intensity the development of the blue colony coloration. To calculate the proportion of Dup+ cells, the number of blue colonies was divided by the total number of, both blue and white, colonies.

### Plate reader assay

Reporter strains were grown for 18 hours in 2 mL LSLB at 37˚ C. Each culture was then diluted to 0.1 OD_600_ in 2 mL M63 in polystyrene tubes and grown at 37˚ C. All planktonic cells and Dup+ cells contained within a biofilm were collected after 24 hours of growth. Strains locked as Dup– were passaged every 24 hours with 2 mL M63 for 96 hours, at which point planktonic cells and cells contained within a biofilm were collected. At the time of collection, cells contained within a biofilm were washed off the sides of the tubes with 1 mL PBS. All cells were then washed in PBS twice and fixed with 2% paraformaldehyde.

A clear 96-well plate was used to measure the OD_600_, and a black 96-well plate was used to measure the absolute fluorescence intensity (AFI) of each sample at ex. 485 nm, em. 535 nm. Planktonic cells were added to each plate at a 1:10 dilution in PBS to a final volume of 200 μL, and 200 μL of cells recovered from the biofilm were added to each plate. The OD_600_ and AFI of PBS were subtracted from the recorded values of each sample to account for the autofluorescence of our media. In order to compare fluorescence intensities between samples, we calculated the relative fluorescence intensity (RFI) of each by dividing the sample’s normalized AFI by the sample’s normalized OD_600_. The RFI is a measurement of fluorescence intensity standardized to bacterial growth (measured through OD_600_) and is reflective of each sample’s detected fluorescence signal relative to background.

### Flow cytometry

Fixed cells used in the plate reader assay were subsequently analyzed using flow cytometry. Each sample was stained with 0.05 mM SYTO 61 red fluorescent nucleic acid stain to distinguish cells from electronic noise and debris within the sample. Events positive for SYTO 61 were then used to determine the gating parameters for forward and side scatter to identify single cells. Analysis was performed with FlowJo software.

## Supporting information

S1 Fig Schematic of modified plasmid rescue strategy used to clone each subregion. Each plasmid is a suicide plasmid for Burkholderia species and contains a ~ 500 bp fragment of DNA corresponding to a junction between subregions, as indicated by the colored boxes, and an I-Scel restriction endonuclease site (yellow box outlined in black), as well as a gene encoding either kanamycin resistance (purple box) or chloramphenicol resistance (dark pink box). Plasmids containing subregions that were generated are shown across the top and bottom. For example, integration of plasmids pLL79 and pLL89 resulted in a strain from which DNA was extracted, digested with I-Scel, and ligated, resulting in the formation of plasmid pLL98.
(TIF)

S2 Fig Schematic of the modified plasmid rescue scheme. The plasmid rescue scheme to clone subregion 4 is shown. First, a Kmr cointegrant containing pLL82, which integrates at the junction between subregion 3 and subregion 4 was obtained. Then, a Cmr derivative of that strain containing pLL92 integrated at the junction between subregion 4 and subregion 5 was obtained. Genomic DNA was obtained from the strain containing both cointegrated plasmids, digested with I-Scel and the fragmented DNA was ligated and used to transform E. coli DHSa. E. coli colonies were screened for those containing pLL101. All strains and plasmids were confirmed to be as expected by PCR and/or DNA sequence analysis.
(TIF)

S3 Fig Schematic of constructing strains containing a duplication of only a single subregion. A schematic of constructing a strain containing a duplication of subregion 4 is shown. Plasmid pLL101 was delivered to BtE264 AISß, which cannot duplicate the 208.6 kb region, by conjugation and Kmr cointegrants were obtained. pLL101 integrates within subregion 4 (represented by the x), the only sequences in the plasmid that are homologous with the chromsome (and integration at the correct site was confirmed by PCR). Integration of pLL101 into subregion 4 results in duplication of subregion 4, as shown in the lower schematic.
(TIF)

S1 Raw Data Fig 3 raw data.
(XLSX)

S2 Raw Data Fig 6 raw data.
(XLSX)

S3 Raw Data Fig 7 raw data.
(TIF)

S1 File Fig 1A uncropped images. (ZIP)

S2 File Fig 1B uncropped images. (ZIP)

S3 File Fig 2B uncropped images. (ZIP)

S4 File Fig 3A uncropped images. (ZIP)

S5 File Fig 4D uncropped images. (ZIP)

S6 File Fig 5A uncropped images. (ZIP)

S7 File Fig 5B uncropped images. (ZIP)

S8 File Fig 5C uncropped images. (ZIP)
