# Supplementary figures and images for: DNA duplication in Burkholderia thailandensis induces biofilm formation by activating a two-component regulatory system
Source: PLoS Genet. 2025 May 20;21(5):e1011528. doi: 10.1371/journal.pgen.1011528 (PMC12124856; doi:10.1371/journal.pgen.1011528)

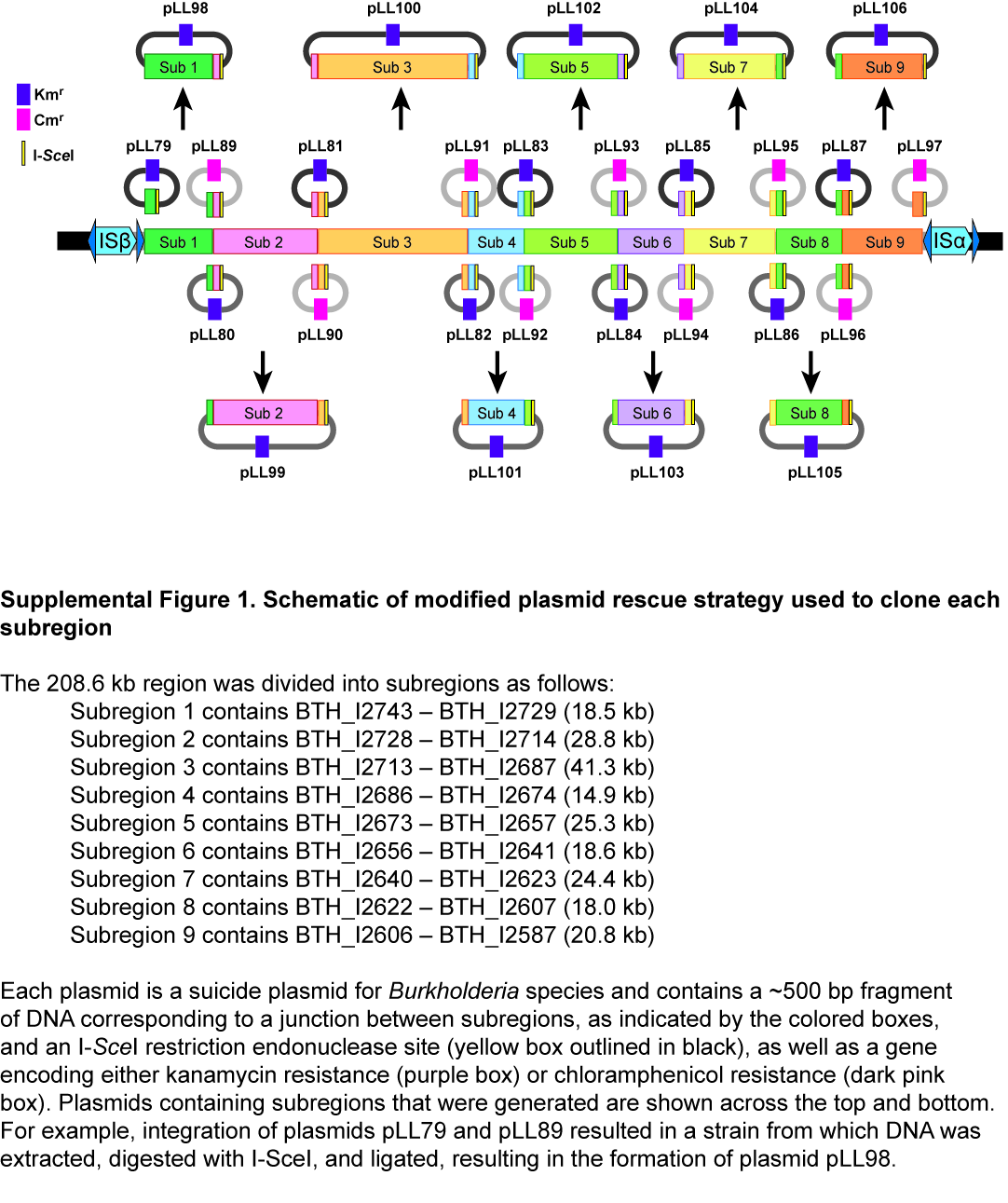

Supplement: S1 Fig — Each plasmid is a suicide plasmid for Burkholderia species and contains a ~ 500 bp fragment of DNA corresponding to a junction between subregions, as indicated by the colored boxes, and an I-Scel restriction endonuclease site (yellow box outlined in black), as well as a gene encoding either kanamycin resistance (purple box) or chloramphenicol resistance (dark pink box). Plasmids containing subregions that were generated are shown across the top and bottom. For example, integration of plasmids pLL79 and pLL89 resulted in a strain from which DNA was extracted, digested with I-Scel, and ligated, resulting in the formation of plasmid pLL98. (TIF) [file pgen.1011528.s001.tif]

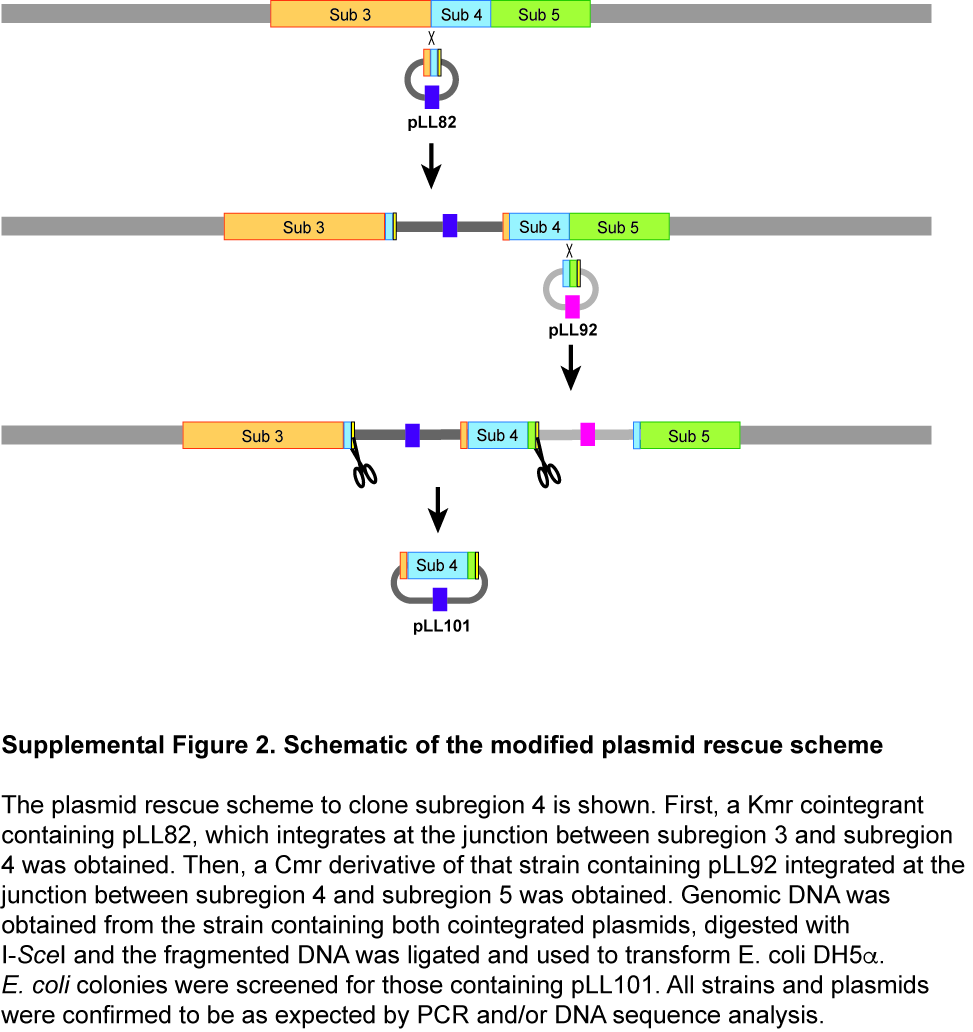

Supplement: S2 Fig — The plasmid rescue scheme to clone subregion 4 is shown. First, a Kmr cointegrant containing pLL82, which integrates at the junction between subregion 3 and subregion 4 was obtained. Then, a Cmr derivative of that strain containing pLL92 integrated at the junction between subregion 4 and subregion 5 was obtained. Genomic DNA was obtained from the strain containing both cointegrated plasmids, digested with I-Scel and the fragmented DNA was ligated and used to transform E. coli DHSa. E. coli colonies were screened for those containing pLL101. All strains and plasmids were confirmed to be as expected by PCR and/or DNA sequence analysis. (TIF) [file pgen.1011528.s002.tif]

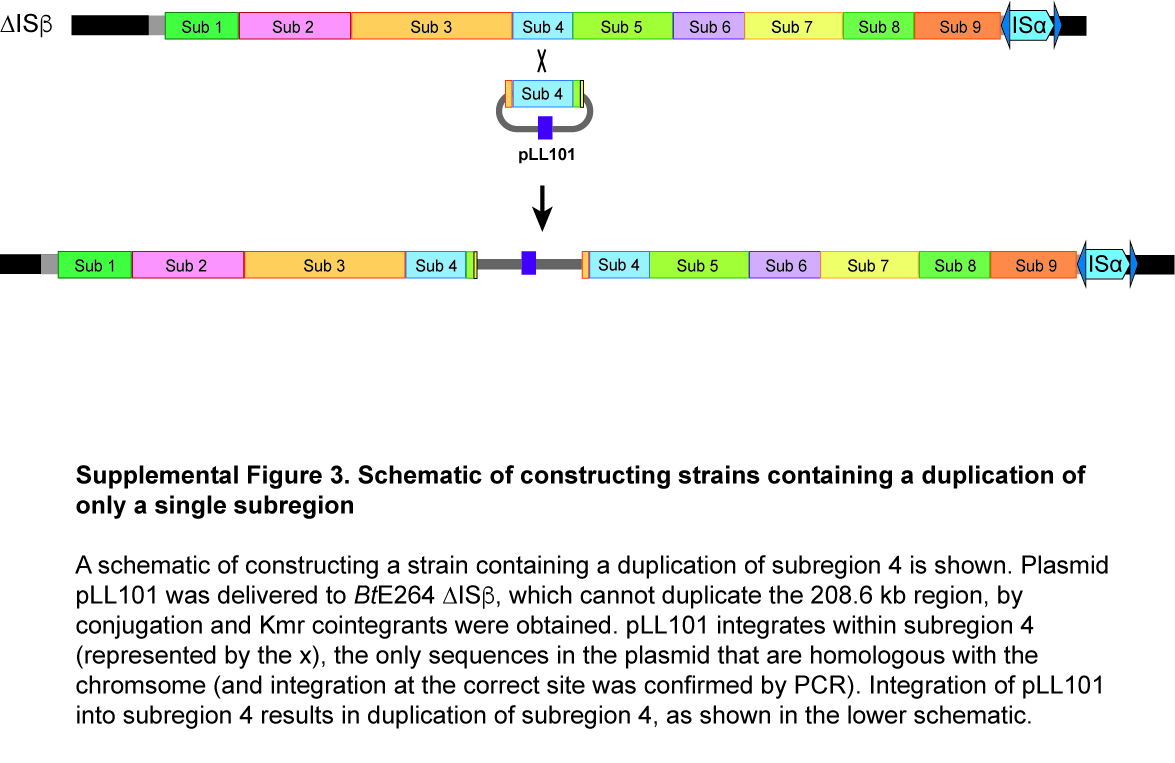

Supplement: S3 Fig — A schematic of constructing a strain containing a duplication of subregion 4 is shown. Plasmid pLL101 was delivered to BtE264 AISß, which cannot duplicate the 208.6 kb region, by conjugation and Kmr cointegrants were obtained. pLL101 integrates within subregion 4 (represented by the x), the only sequences in the plasmid that are homologous with the chromsome (and integration at the correct site was confirmed by PCR). Integration of pLL101 into subregion 4 results in duplication of subregion 4, as shown in the lower schematic. (TIF) [file pgen.1011528.s003.tif]

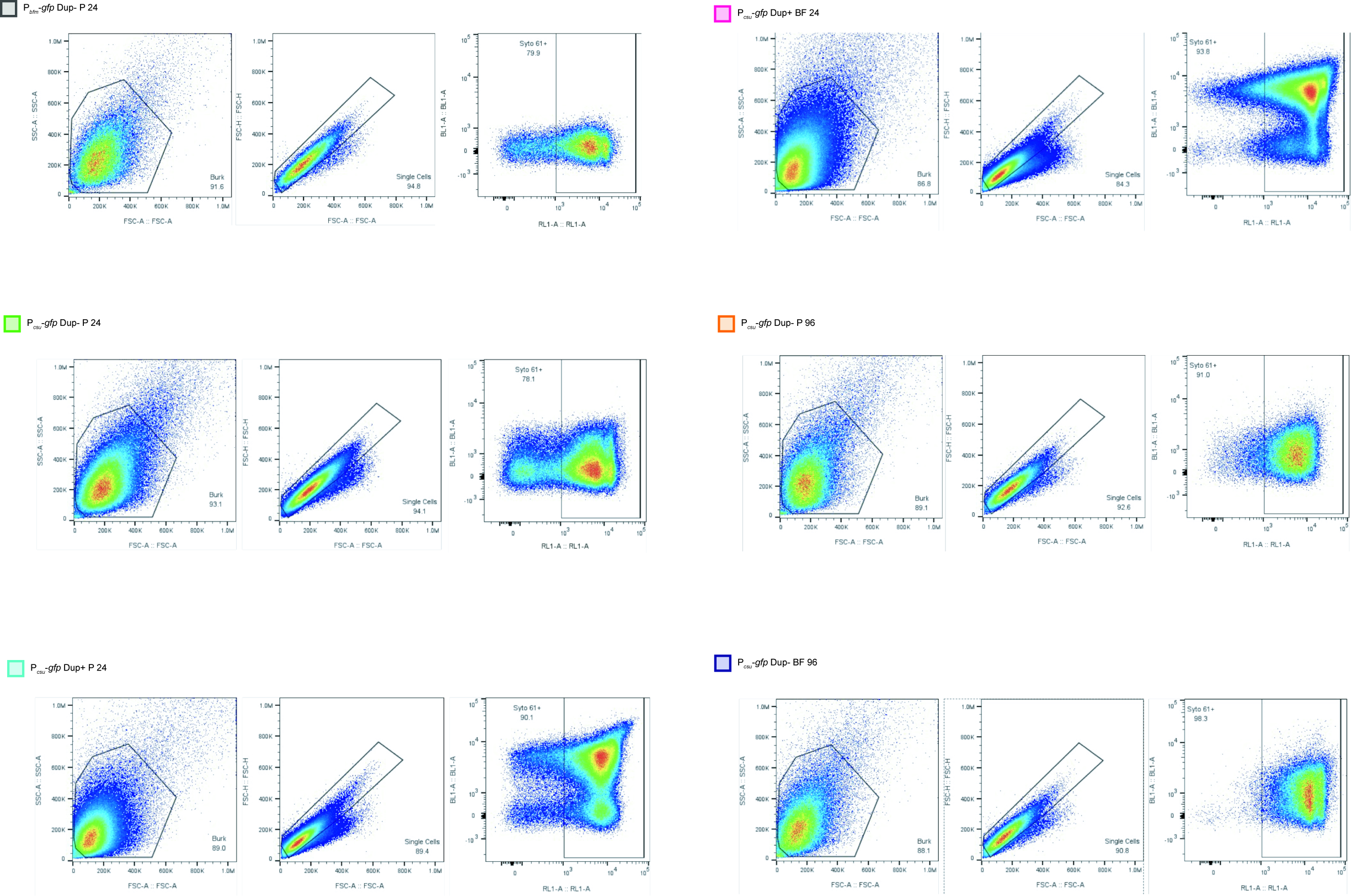

Supplement: S3 Raw Data — Fig 7 raw data. (TIF) [file pgen.1011528.s006.tif]

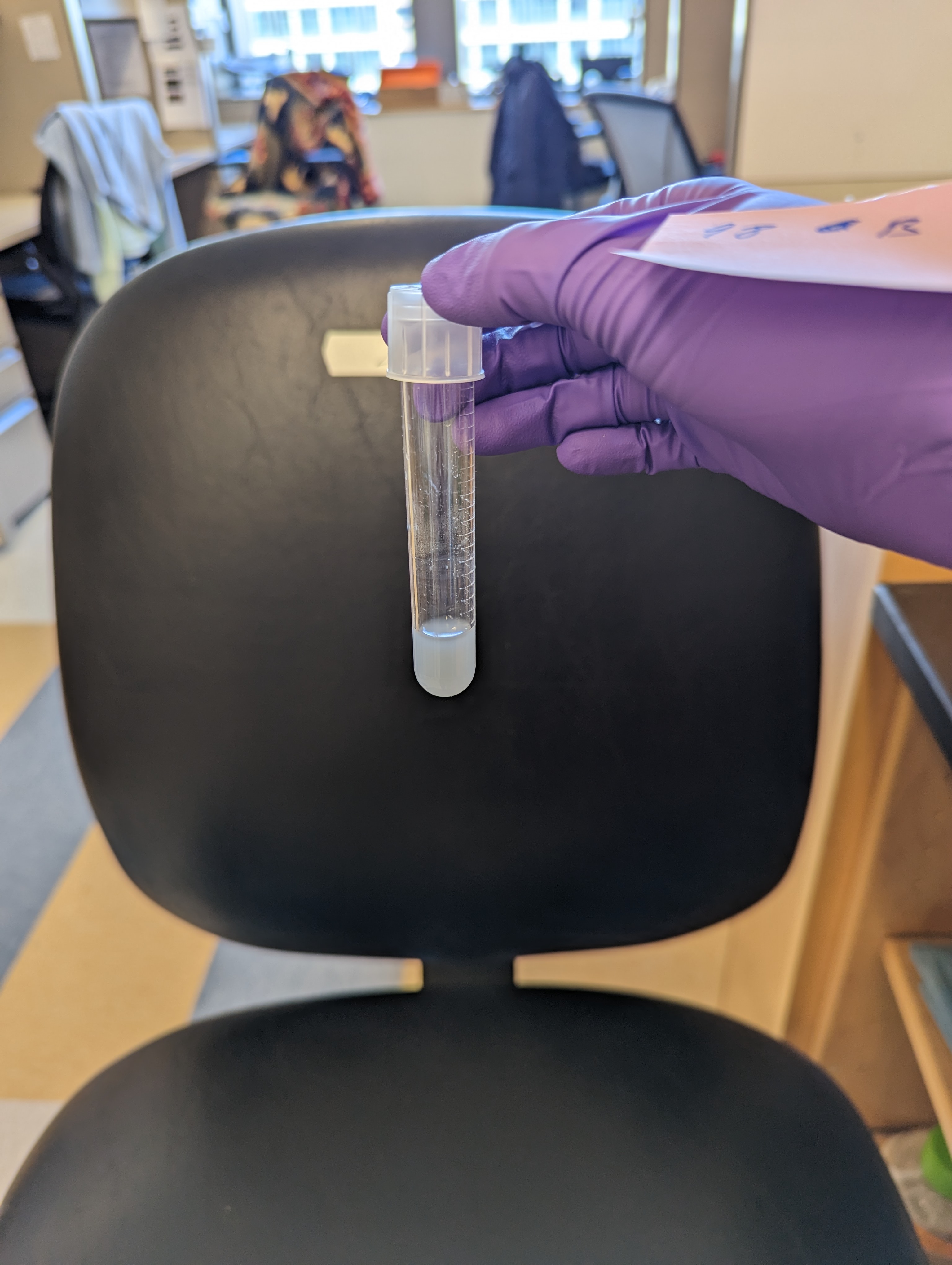

Supplement: S1 File — (ZIP) [file pgen.1011528.s007.zip › Fig 1A/1A duplicate copy of subregion 1.jpg]

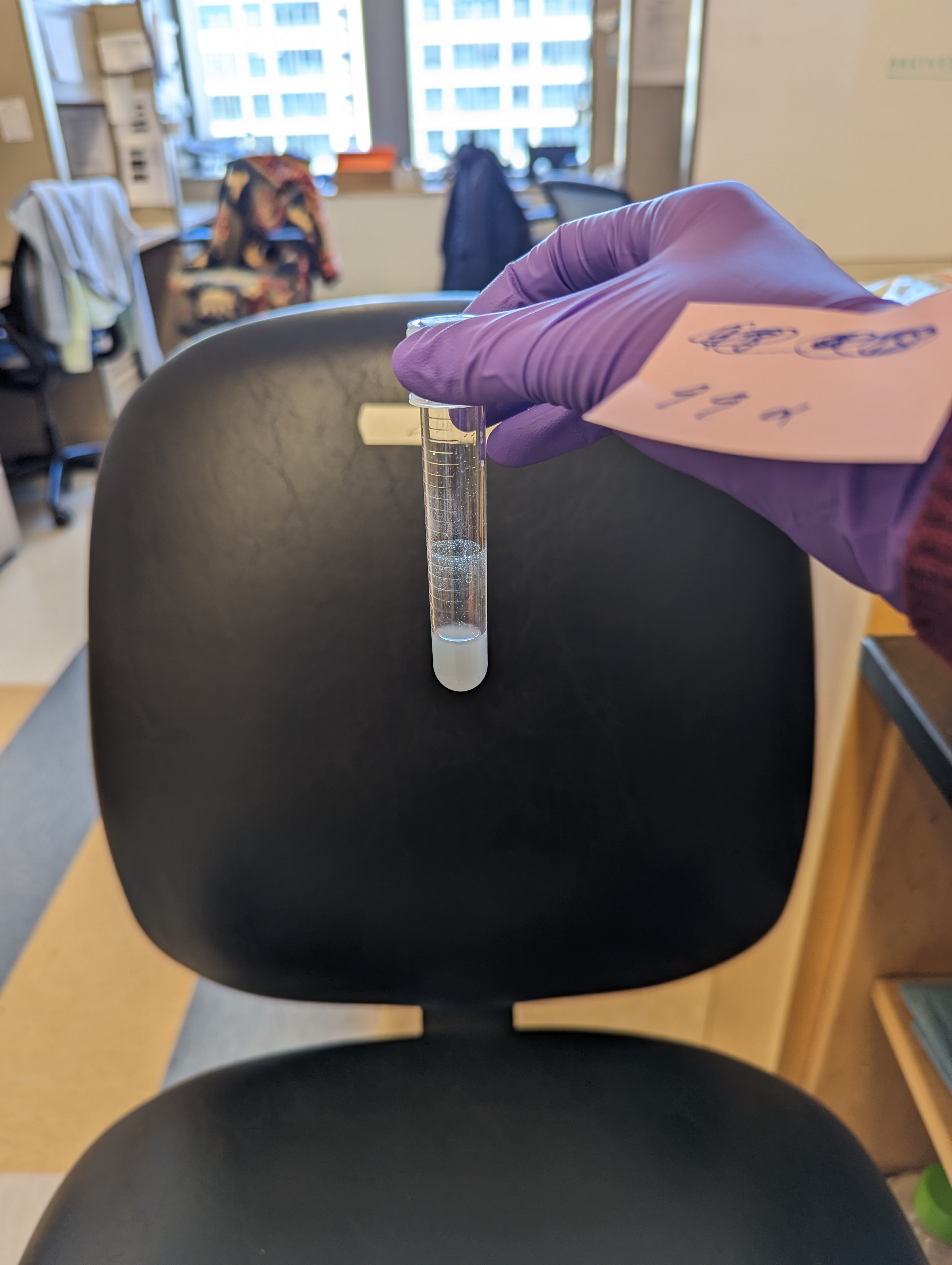

Supplement: S1 File — (ZIP) [file pgen.1011528.s007.zip › Fig 1A/1A duplicate copy of subregion 2.jpg]

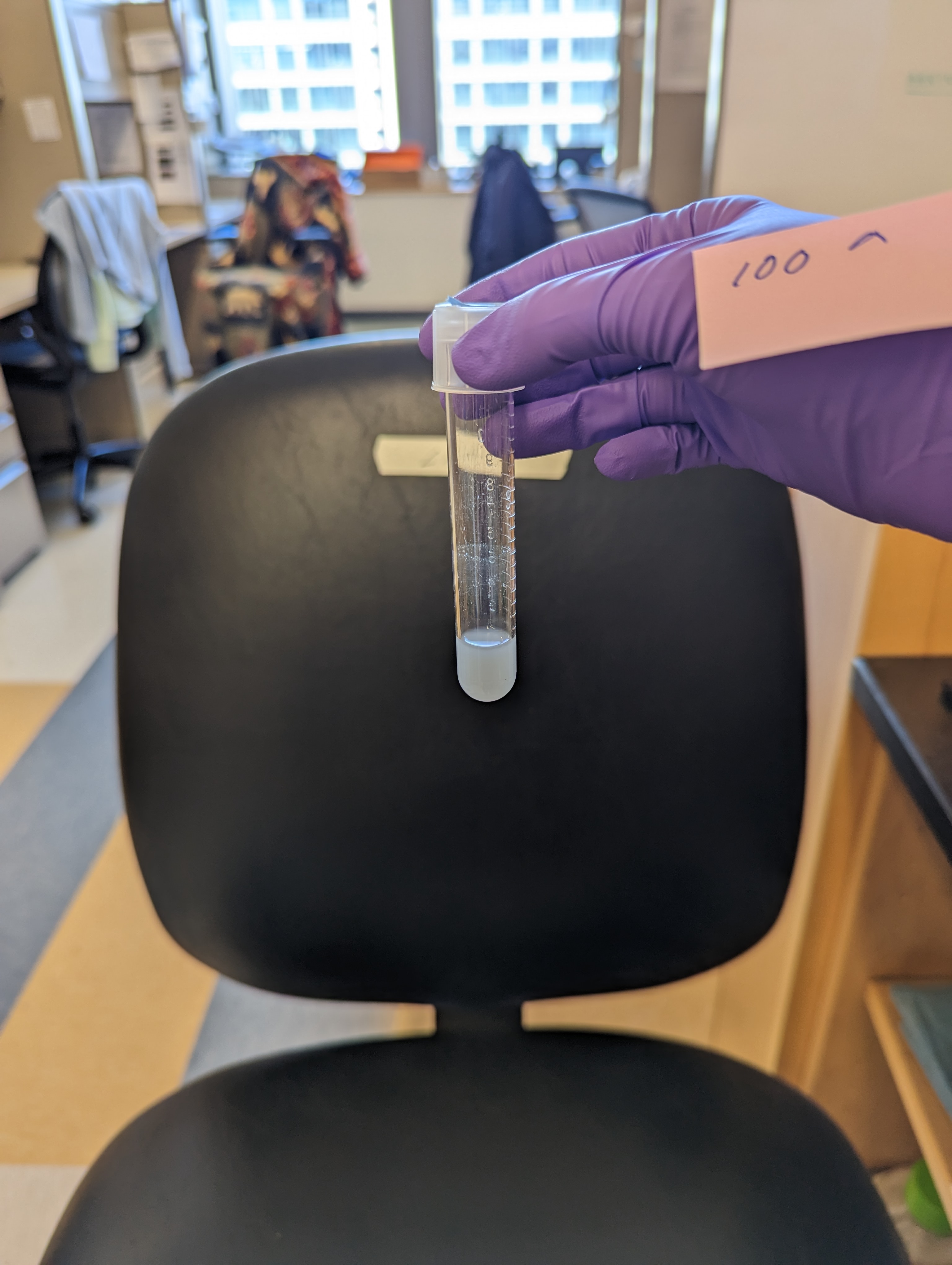

Supplement: S1 File — (ZIP) [file pgen.1011528.s007.zip › Fig 1A/1A duplicate copy of subregion 3.jpg]

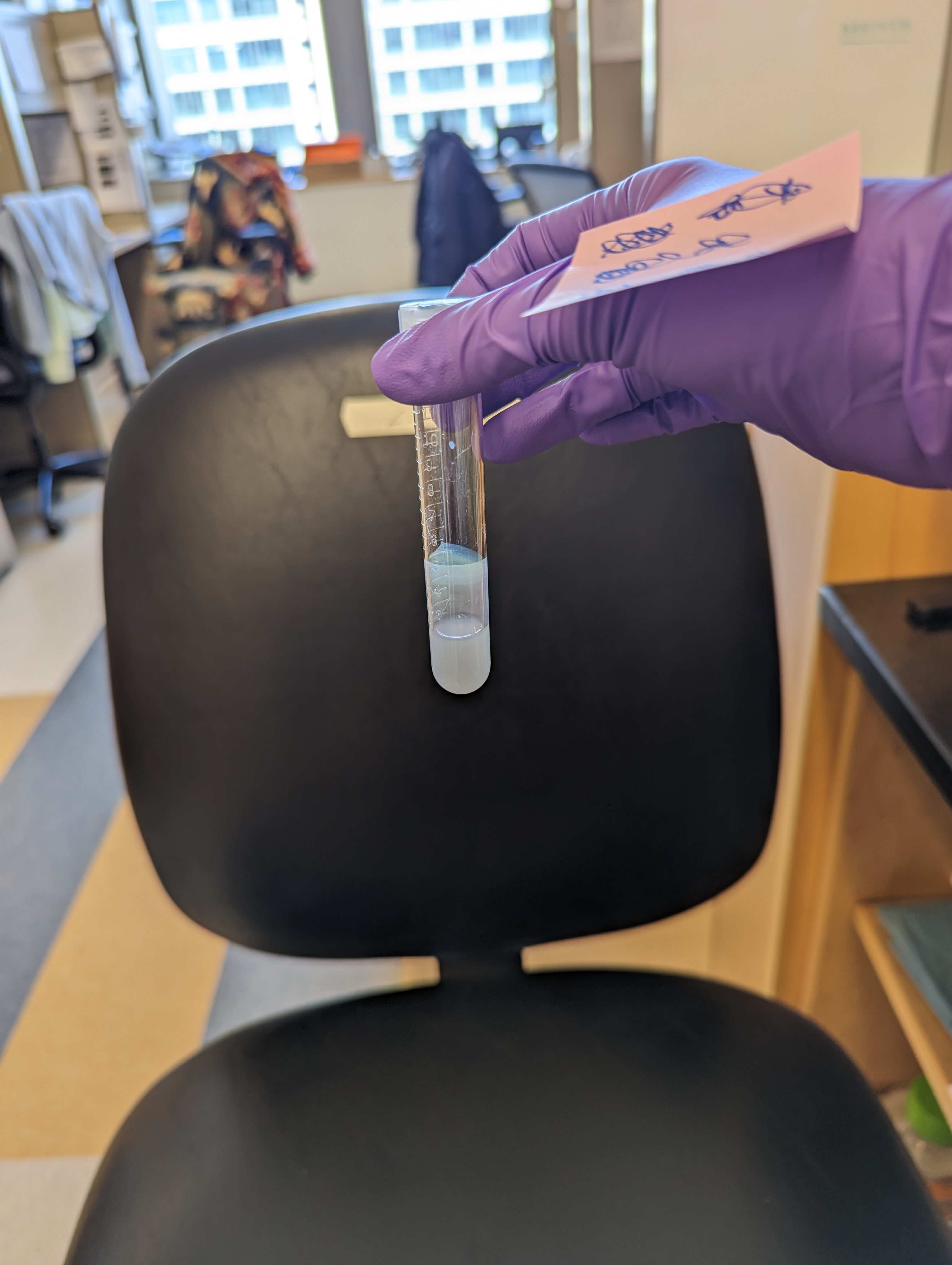

Supplement: S1 File — (ZIP) [file pgen.1011528.s007.zip › Fig 1A/1A duplicate copy of subregion 4.jpg]

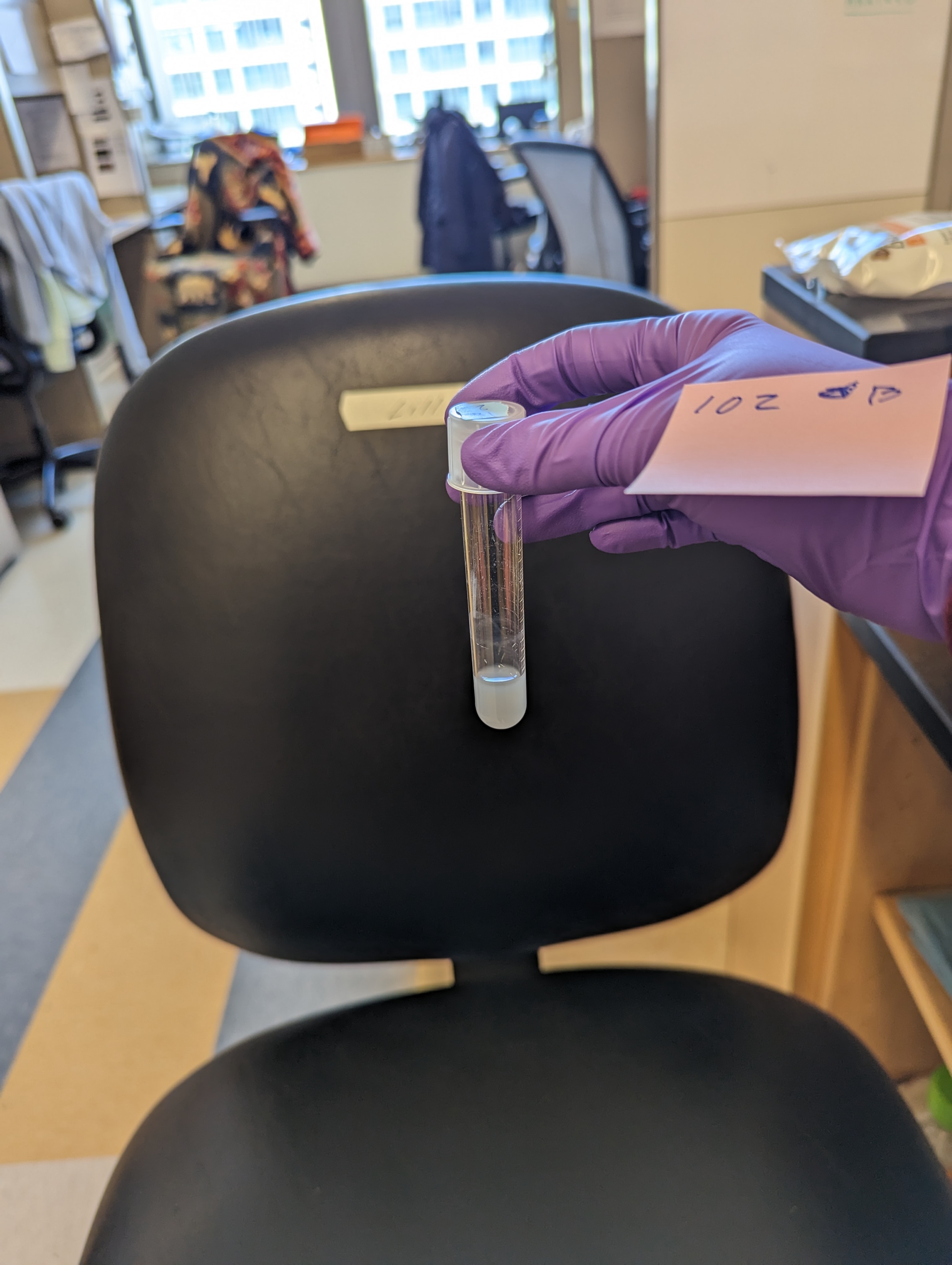

Supplement: S1 File — (ZIP) [file pgen.1011528.s007.zip › Fig 1A/1A duplicate copy of subregion 5.jpg]

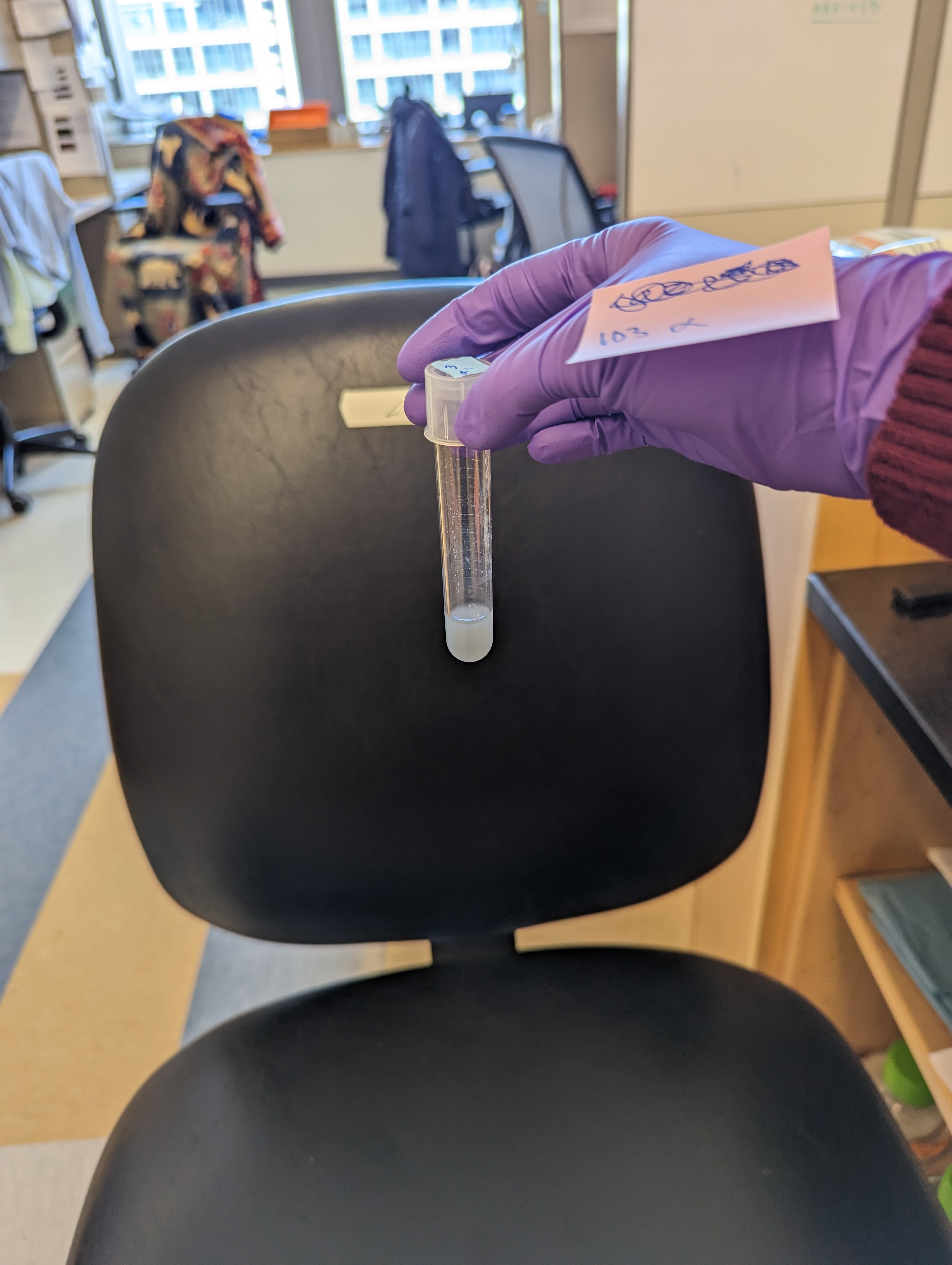

Supplement: S1 File — (ZIP) [file pgen.1011528.s007.zip › Fig 1A/1A duplicate copy of subregion 6.jpg]

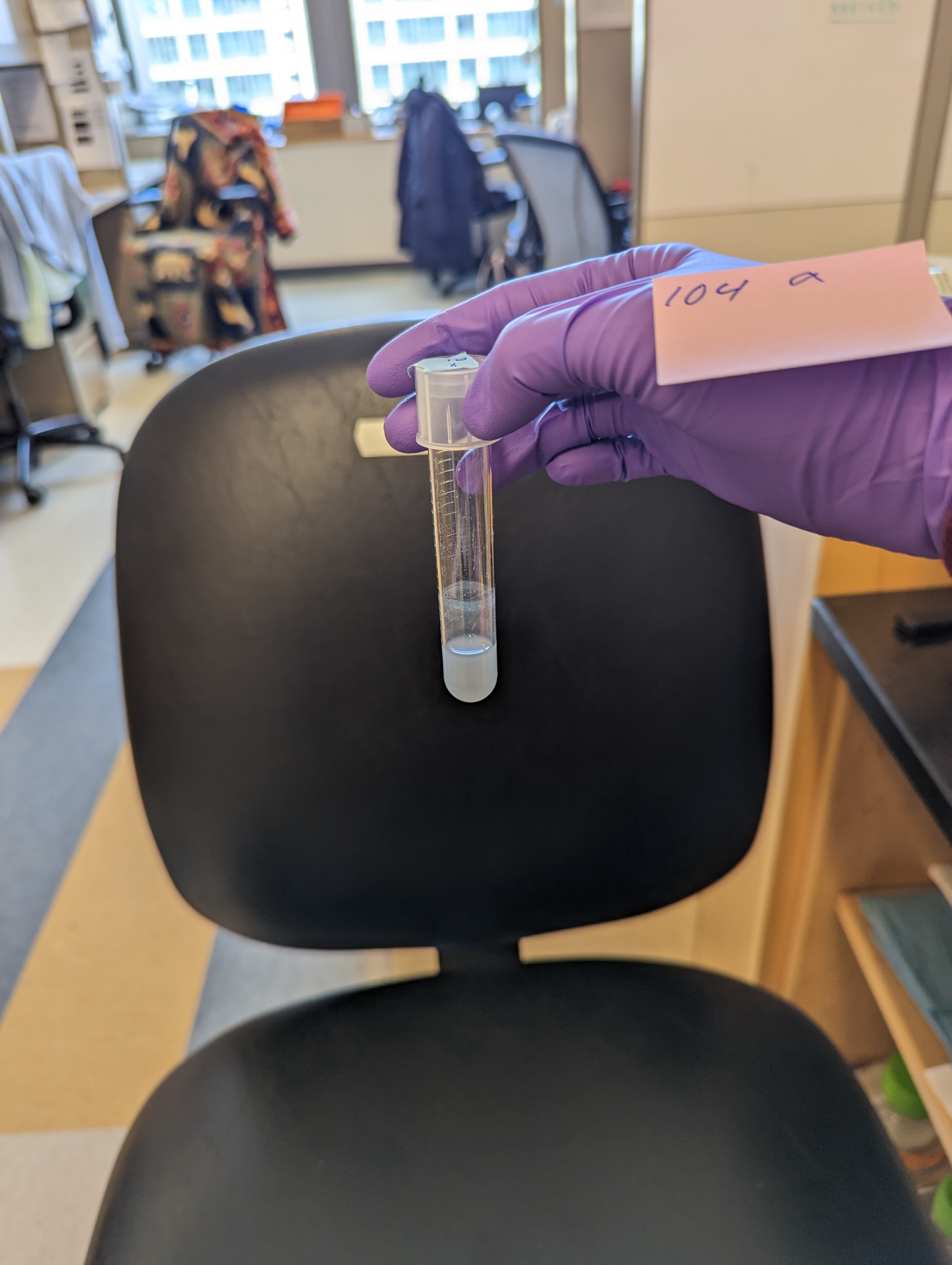

Supplement: S1 File — (ZIP) [file pgen.1011528.s007.zip › Fig 1A/1A duplicate copy of subregion 7.jpg]

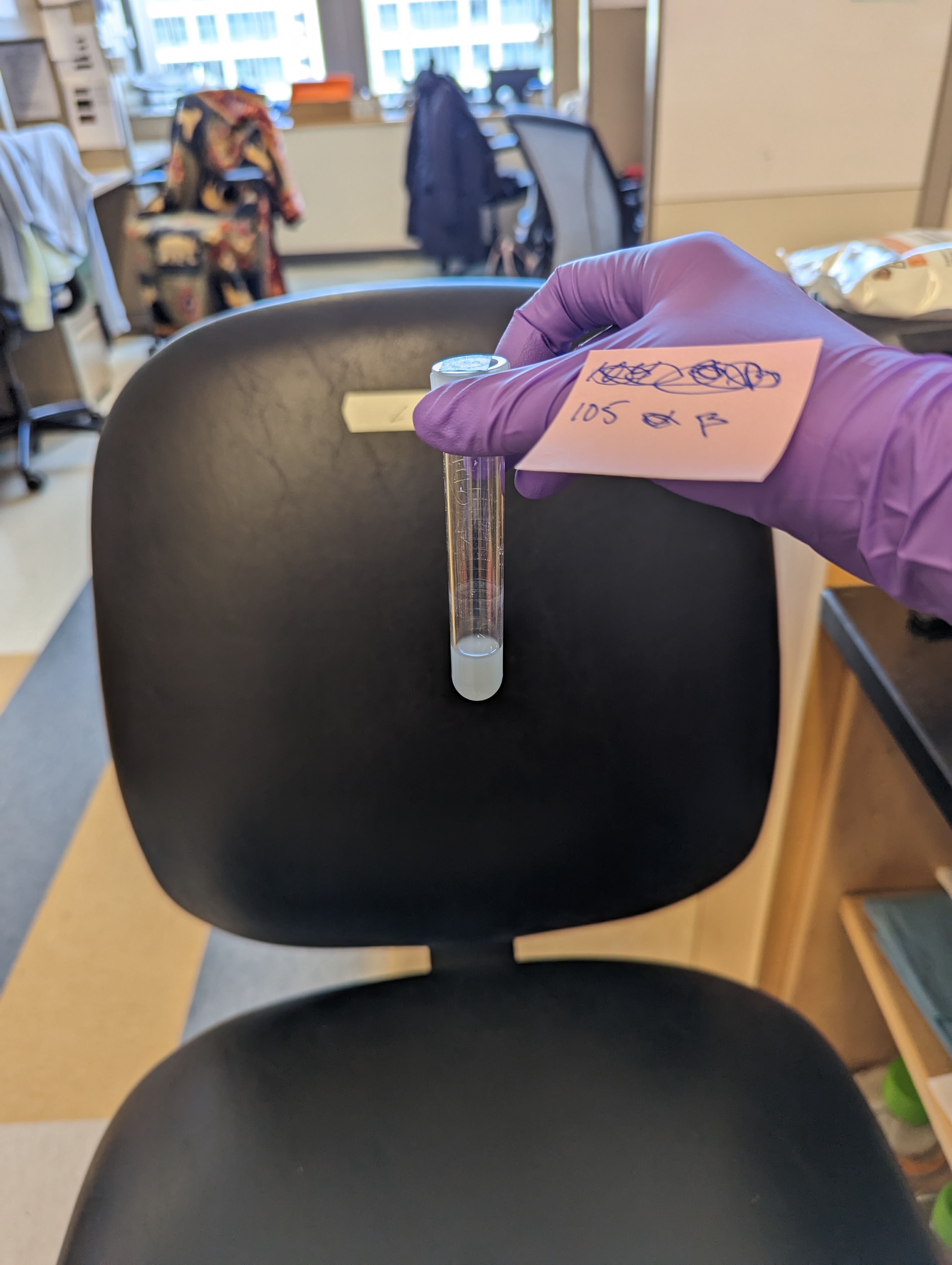

Supplement: S1 File — (ZIP) [file pgen.1011528.s007.zip › Fig 1A/1A duplicate copy of subregion 8.jpg]

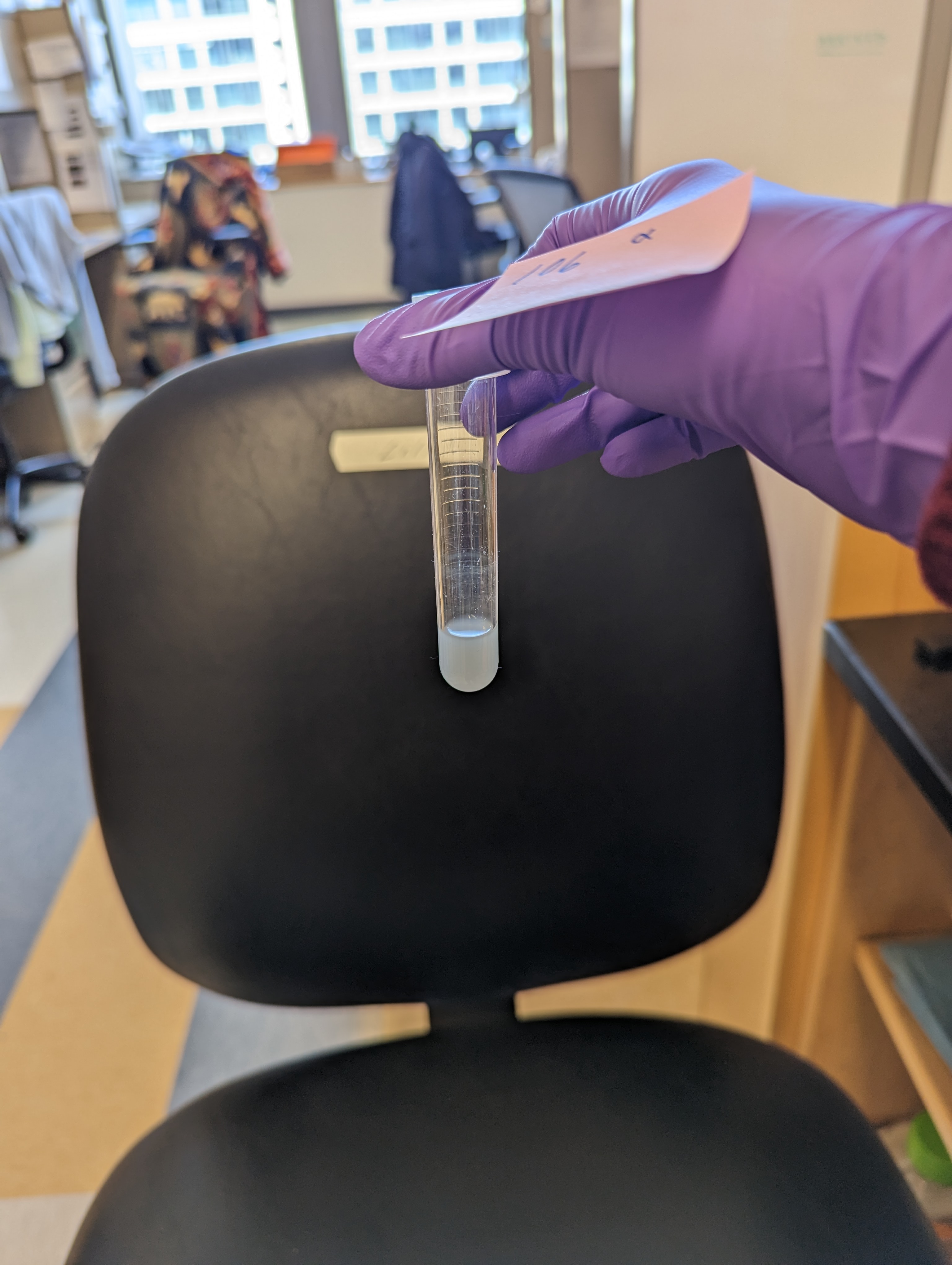

Supplement: S1 File — (ZIP) [file pgen.1011528.s007.zip › Fig 1A/1A duplicate copy of subregion 9.jpg]

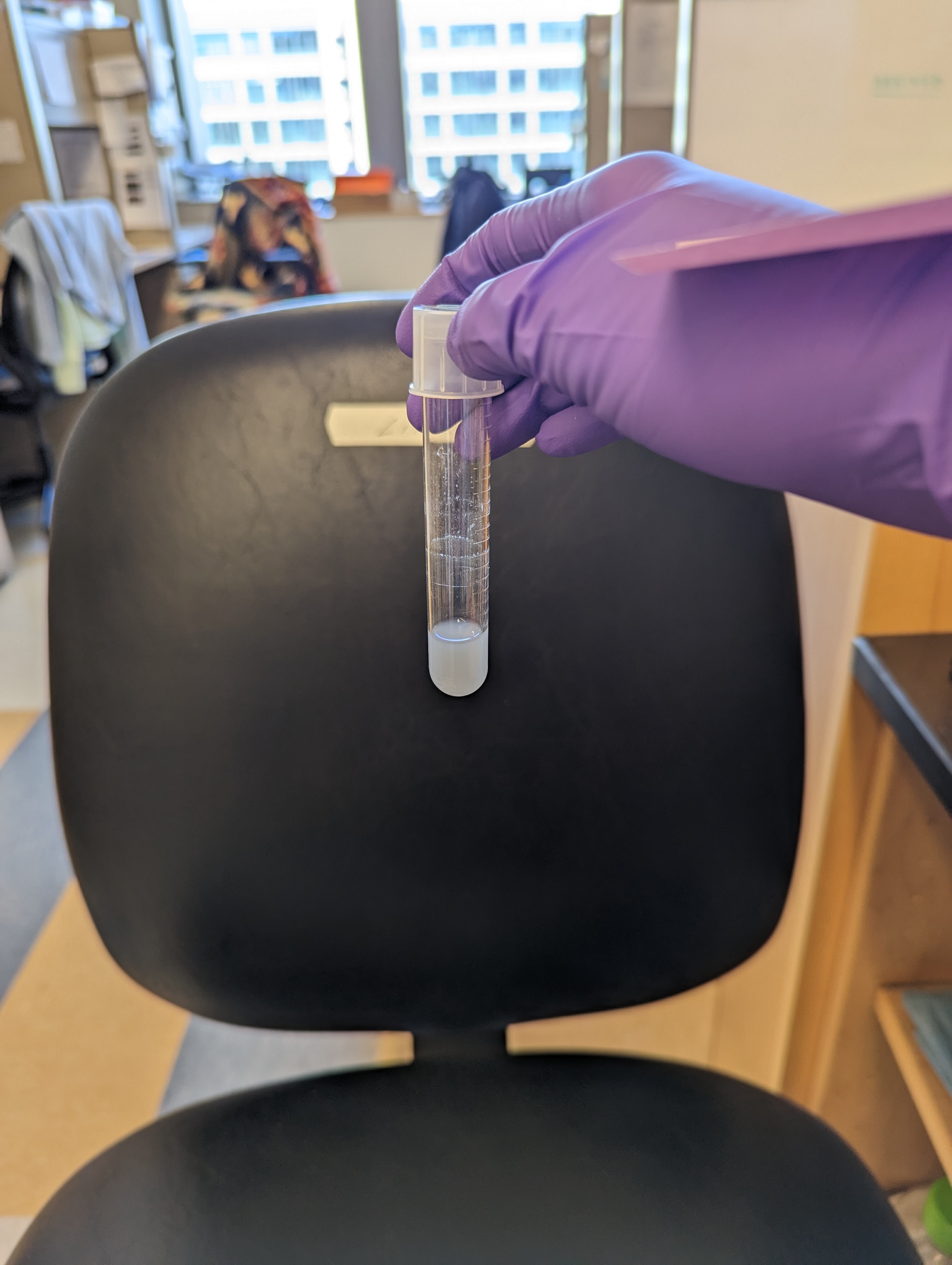

Supplement: S1 File — (ZIP) [file pgen.1011528.s007.zip › Fig 1A/1A Wildtype Dup-.jpg]

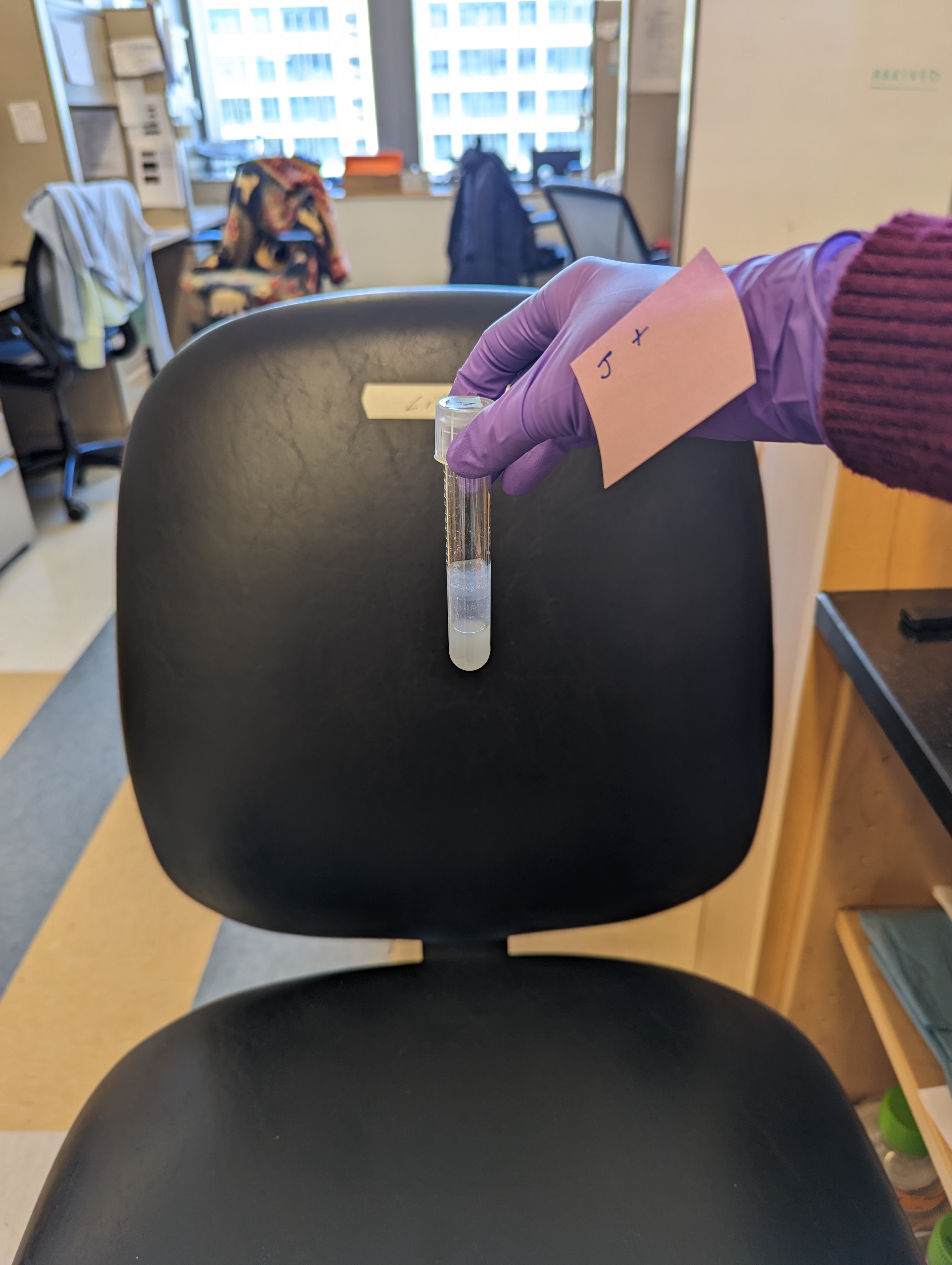

Supplement: S1 File — (ZIP) [file pgen.1011528.s007.zip › Fig 1A/1A Wildtype Dup+.jpg]

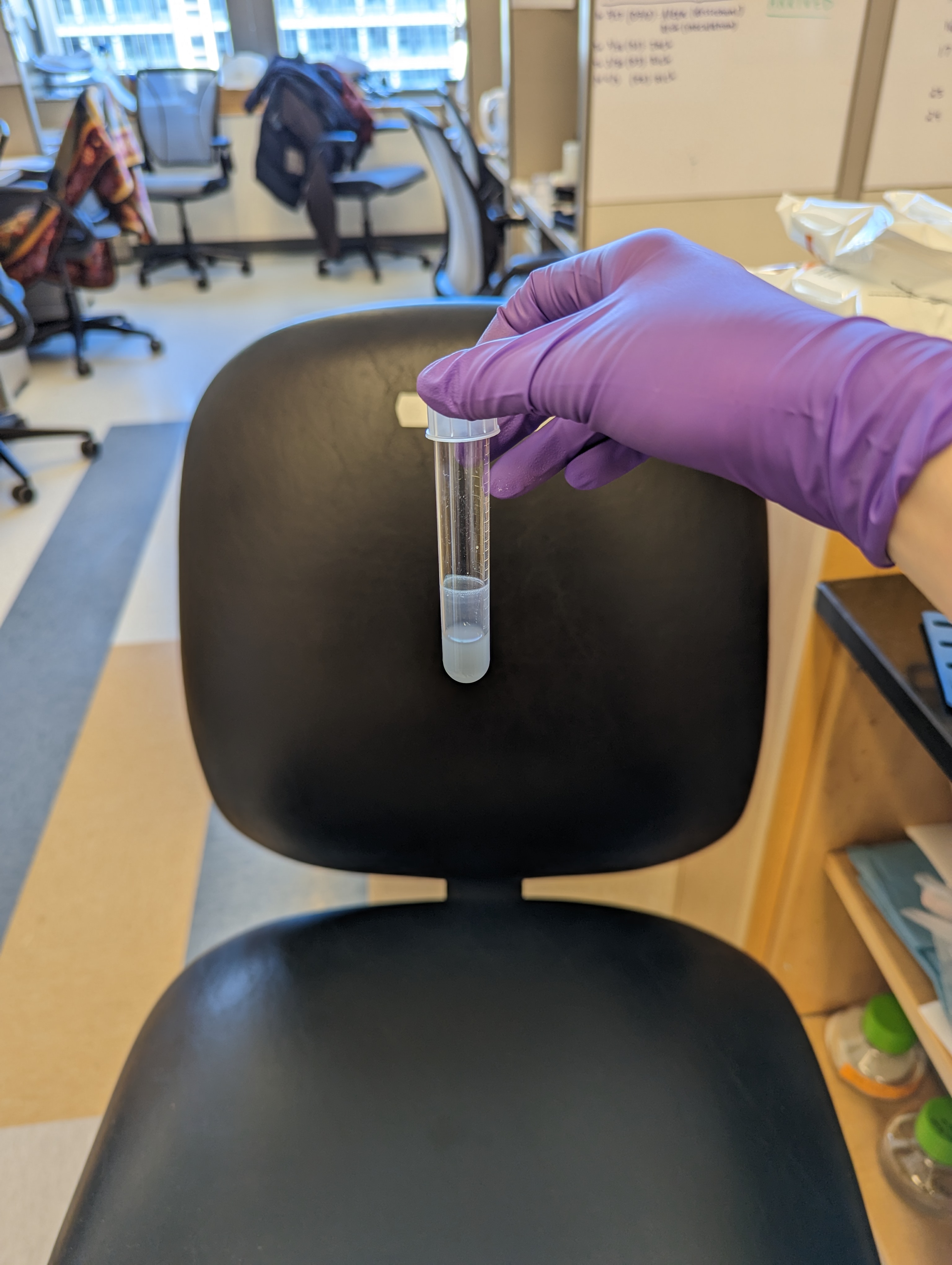

Supplement: S2 File — (ZIP) [file pgen.1011528.s008.zip › Fig 1B/1B duplicate copy of subregion 4.jpg]

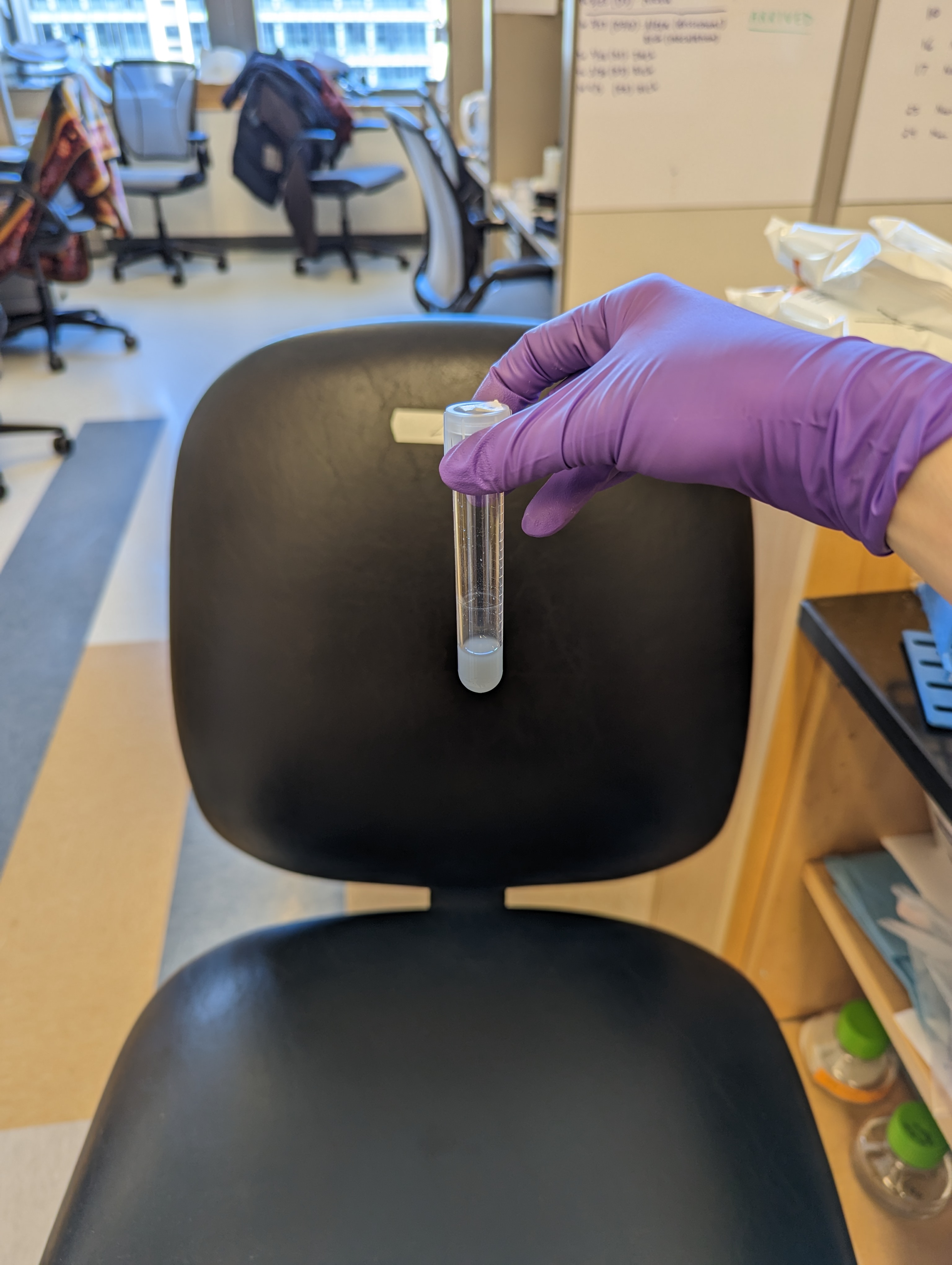

Supplement: S2 File — (ZIP) [file pgen.1011528.s008.zip › Fig 1B/1B duplicate copy of sub-subregion 4.1.jpg]

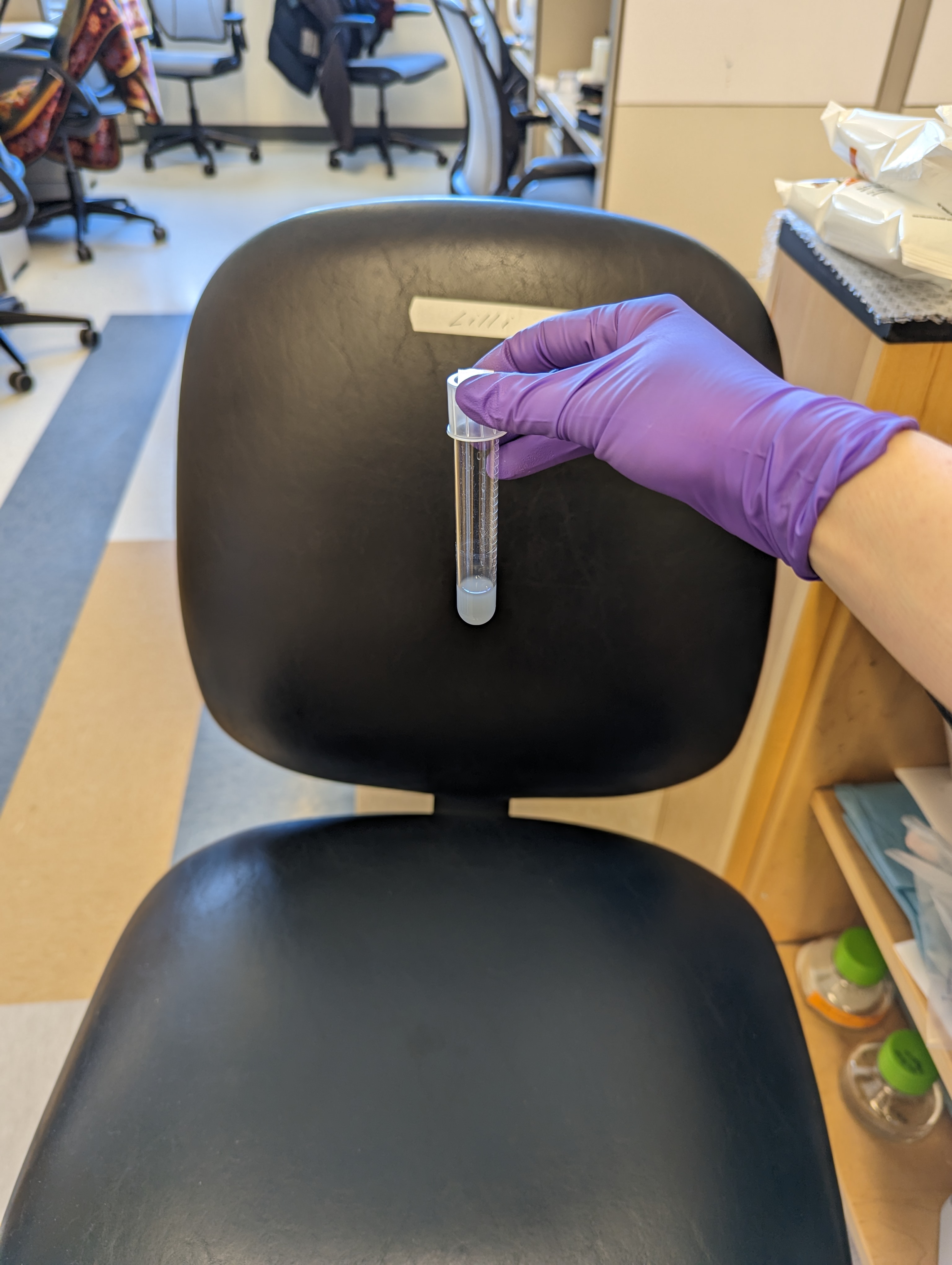

Supplement: S2 File — (ZIP) [file pgen.1011528.s008.zip › Fig 1B/1B duplicate copy of sub-subregion 4.2.jpg]

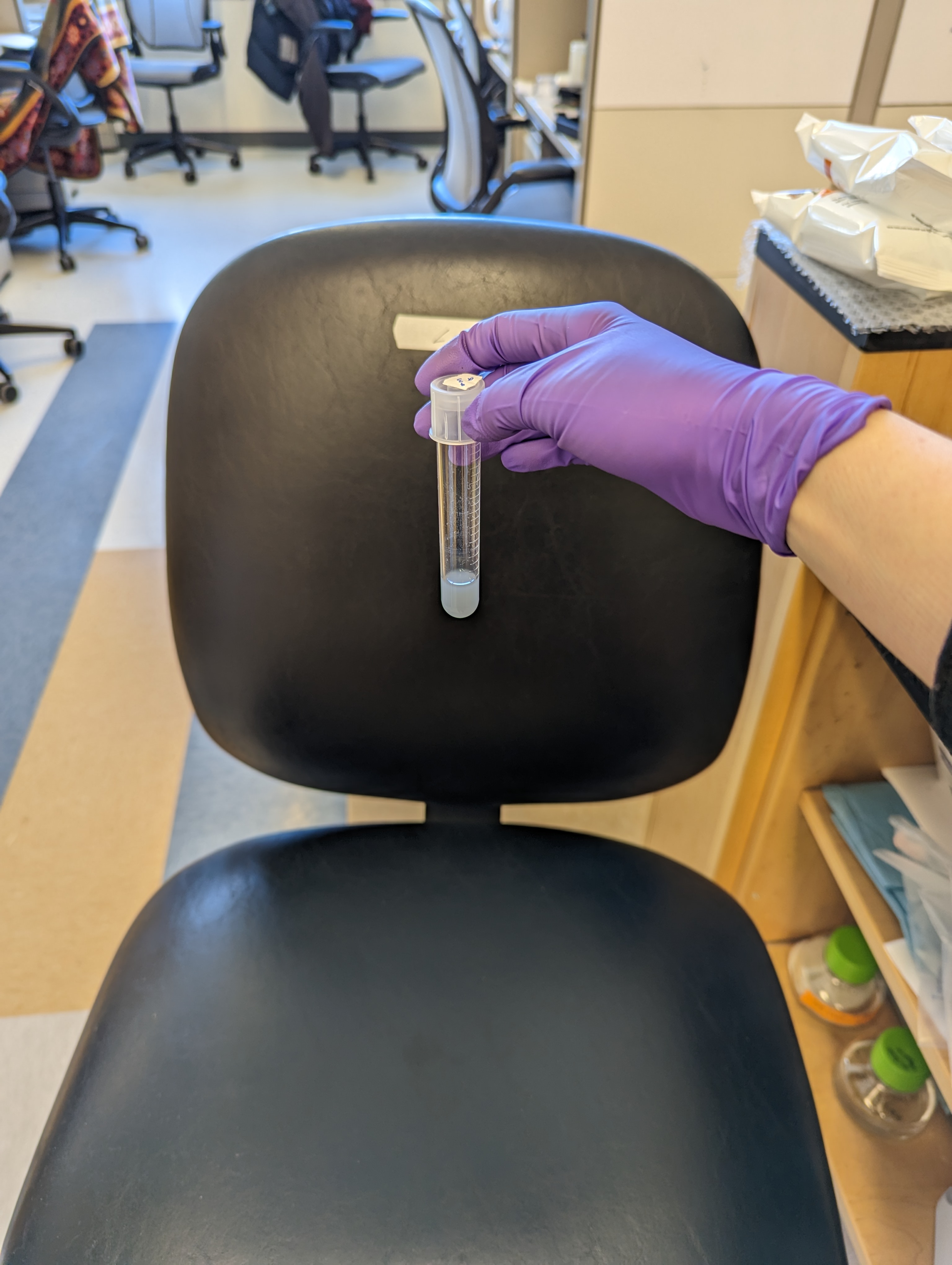

Supplement: S2 File — (ZIP) [file pgen.1011528.s008.zip › Fig 1B/1B duplicate copy of sub-subregion 4.3.jpg]

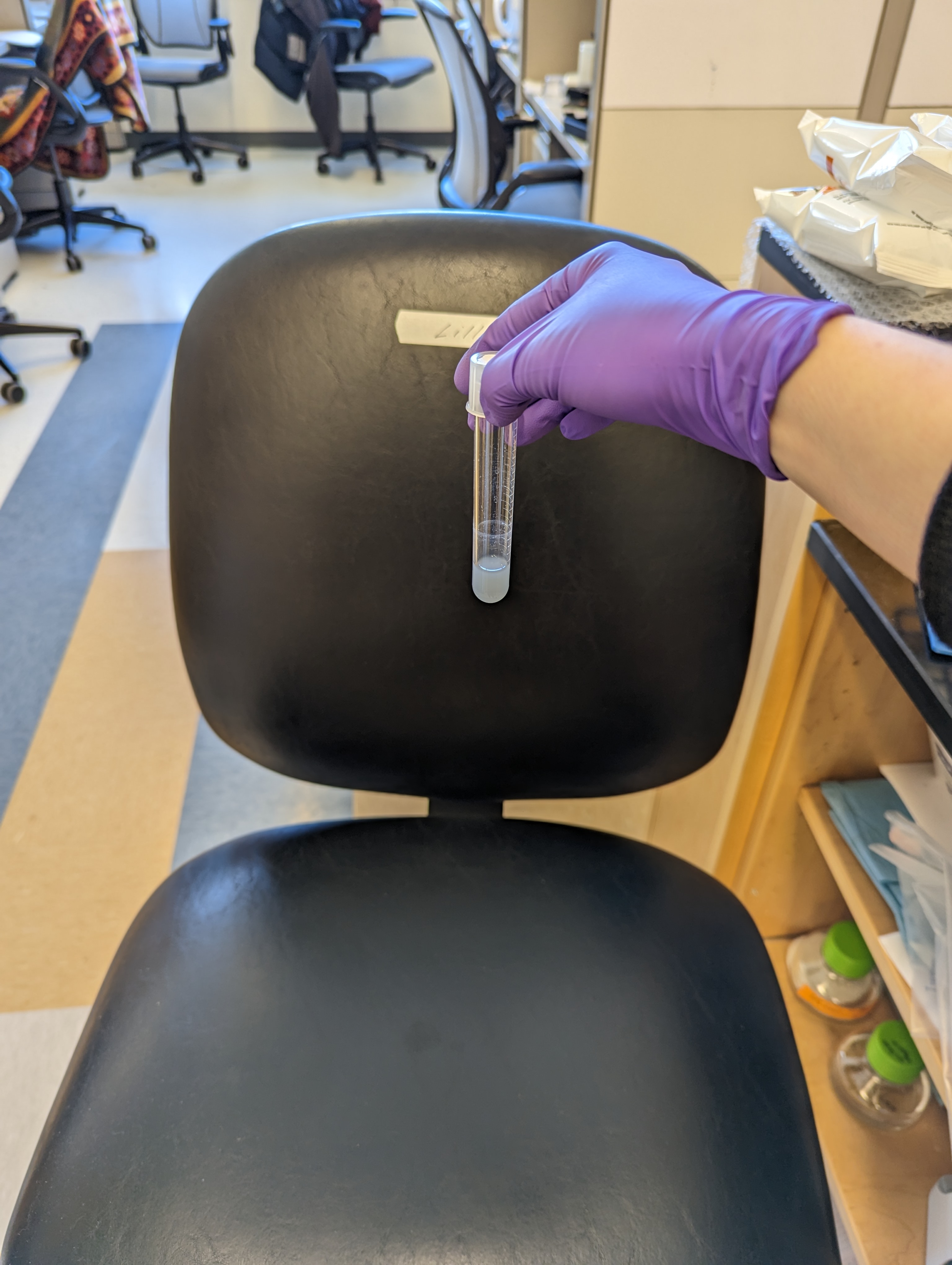

Supplement: S2 File — (ZIP) [file pgen.1011528.s008.zip › Fig 1B/1B duplicate copy of sub-subregion 4.4.jpg]

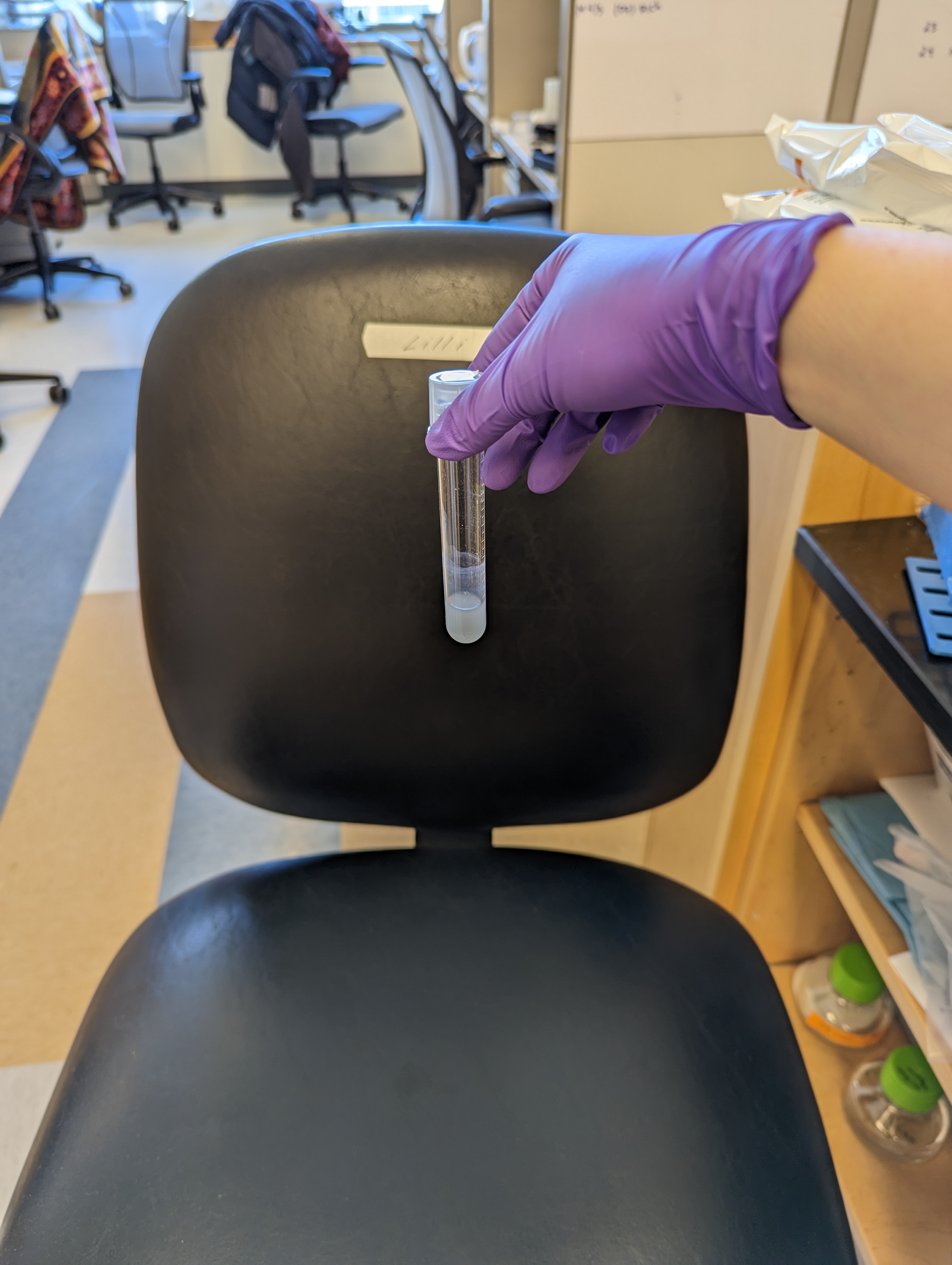

Supplement: S2 File — (ZIP) [file pgen.1011528.s008.zip › Fig 1B/1B duplicate copy of sub-subregion 4.5.jpg]

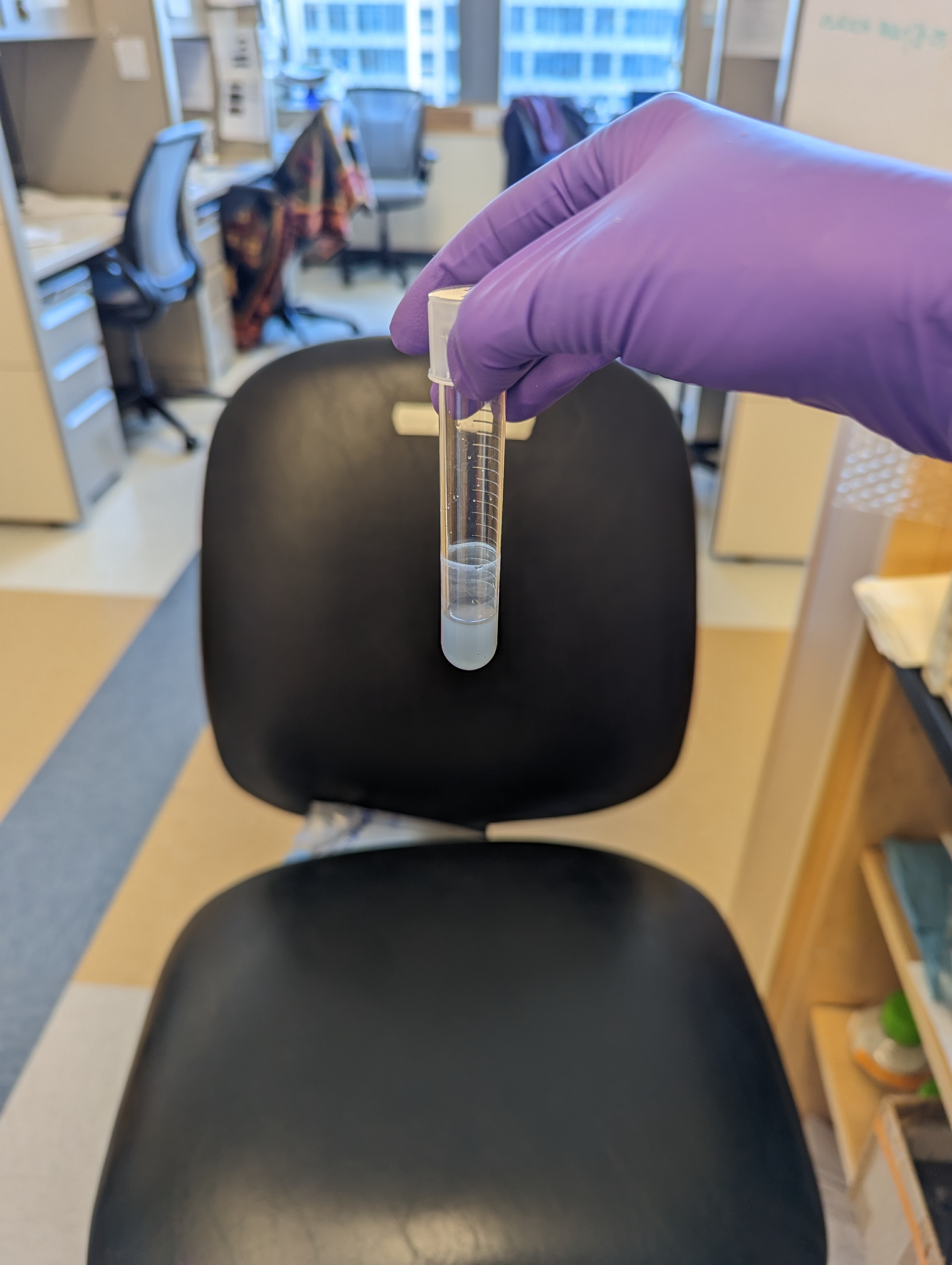

Supplement: S2 File — (ZIP) [file pgen.1011528.s008.zip › Fig 1B/1B duplicate copy of sub-subregions 4.4-5.jpg]

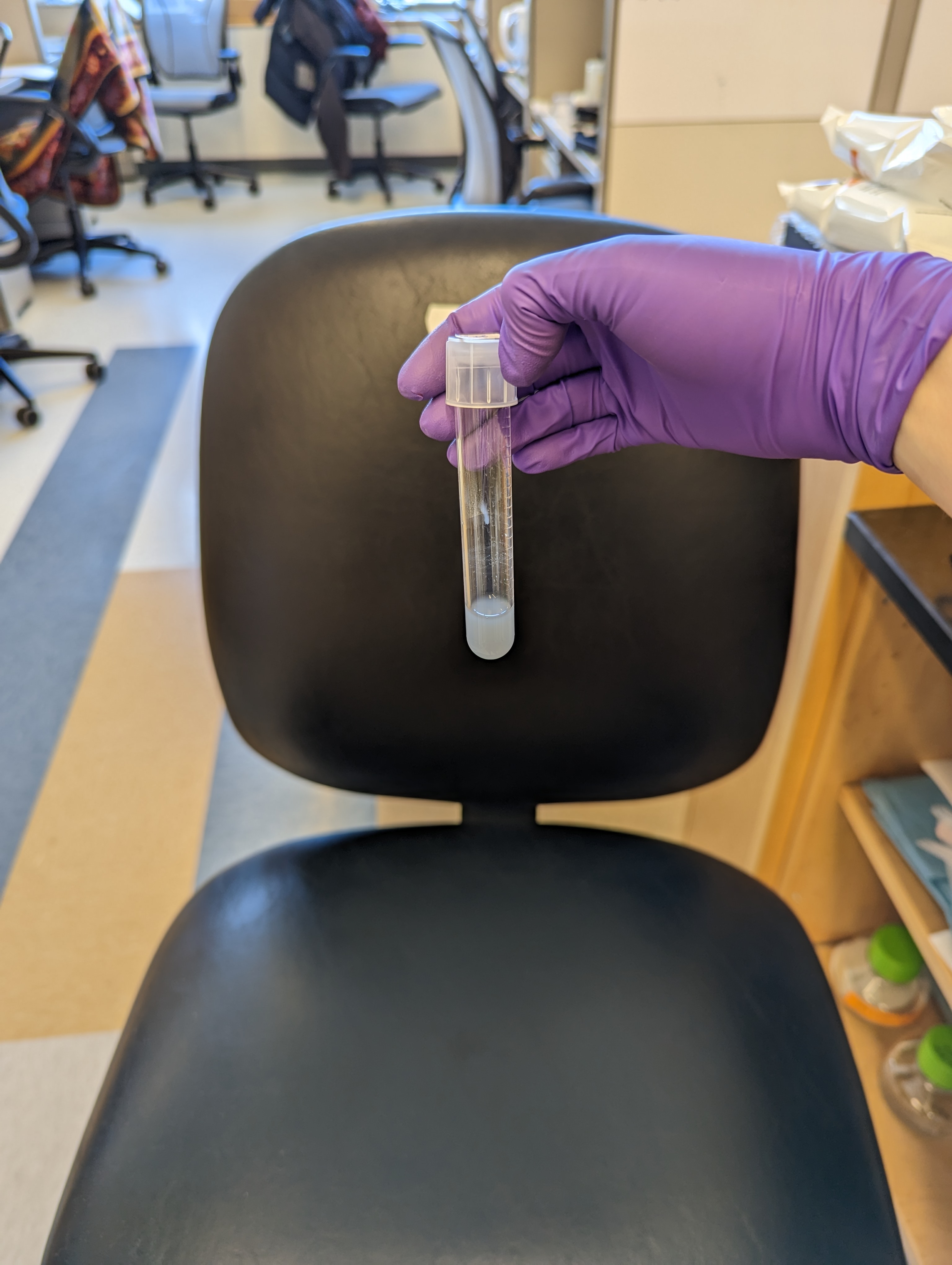

Supplement: S2 File — (ZIP) [file pgen.1011528.s008.zip › Fig 1B/1B Wildtype dup-.jpg]

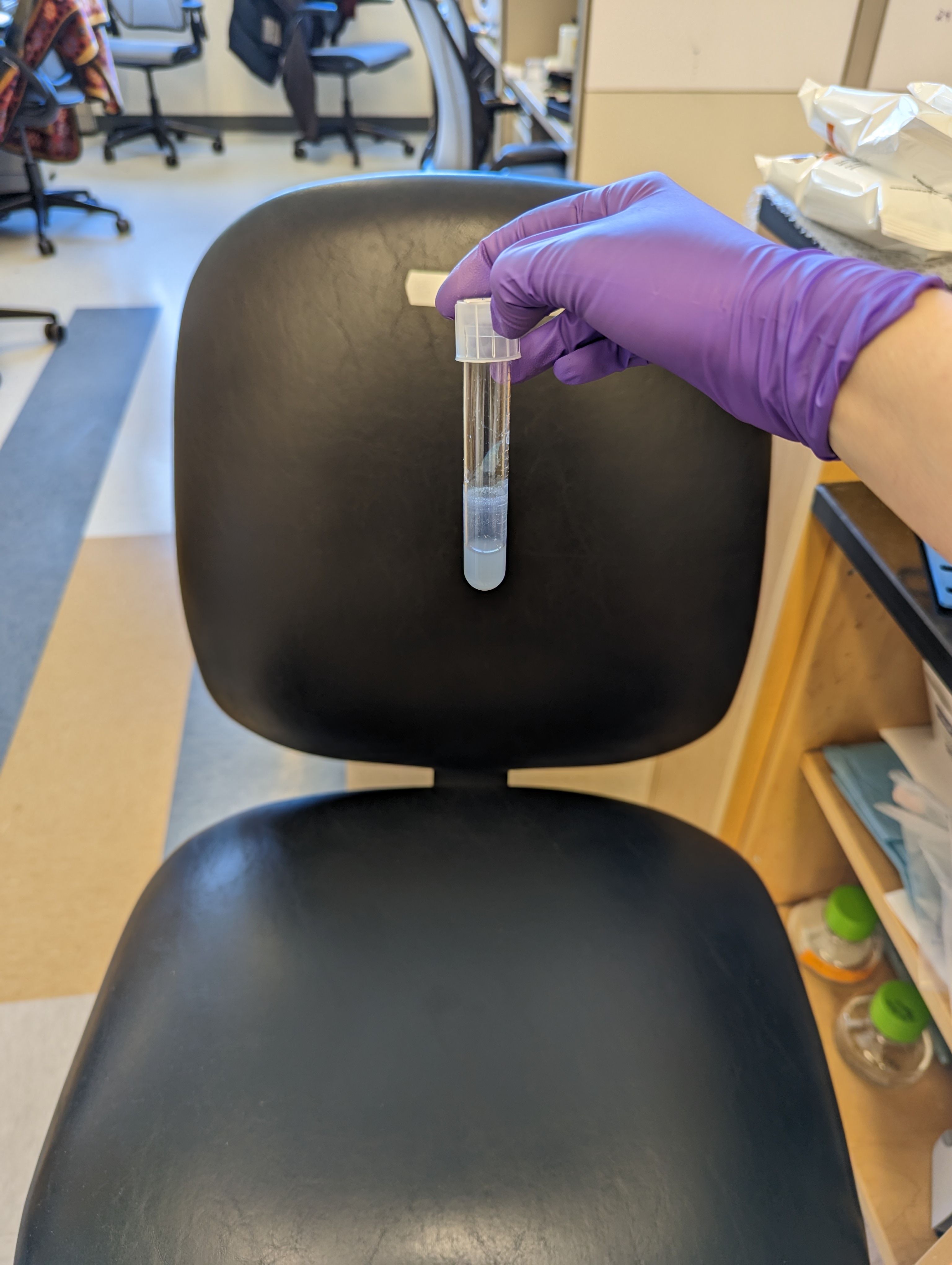

Supplement: S2 File — (ZIP) [file pgen.1011528.s008.zip › Fig 1B/1B Wildtype dup+.jpg]

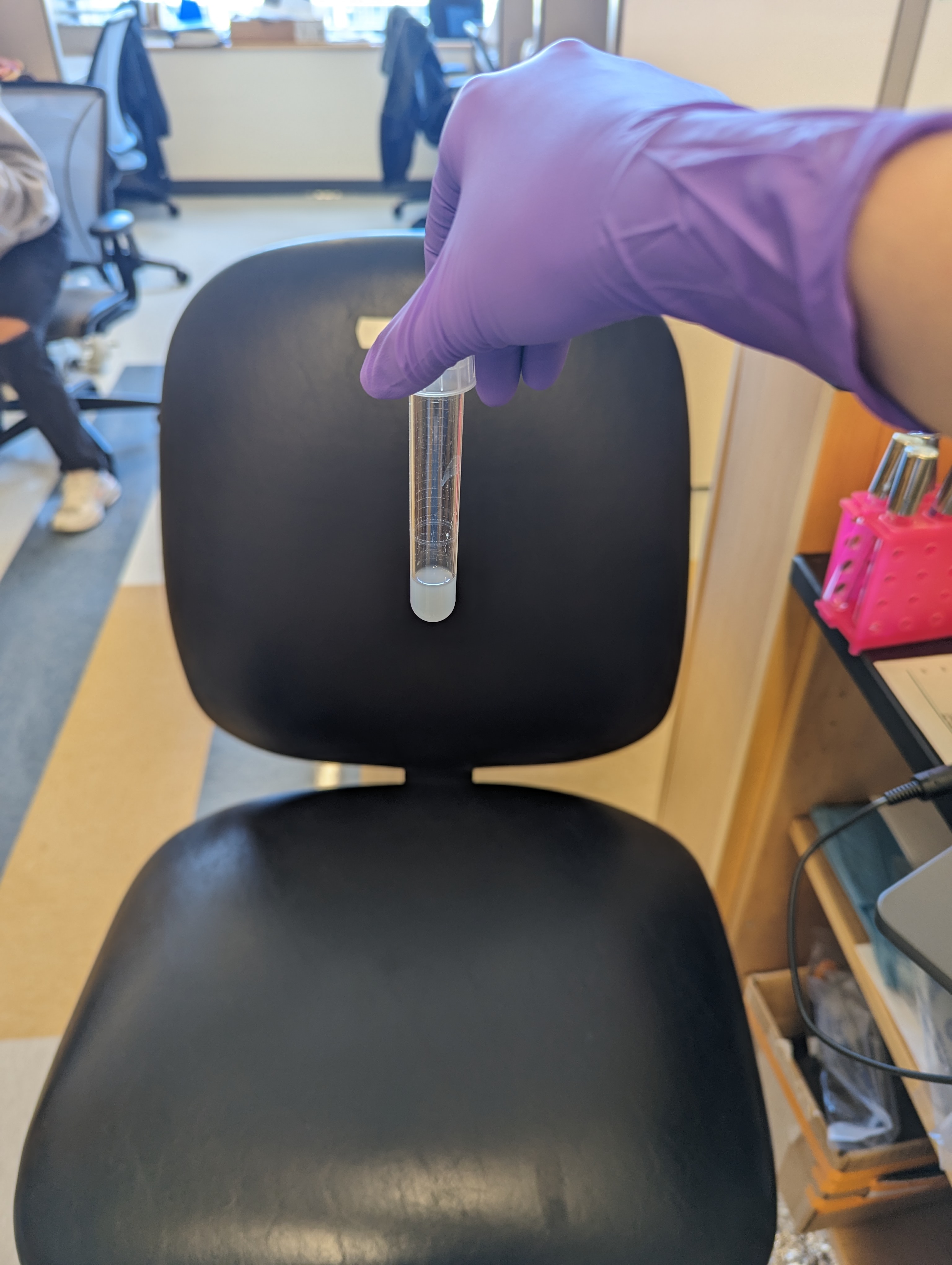

Supplement: S3 File — (ZIP) [file pgen.1011528.s009.zip › Fig 2B/2B frag kan dup- delta bfmSR.jpg]

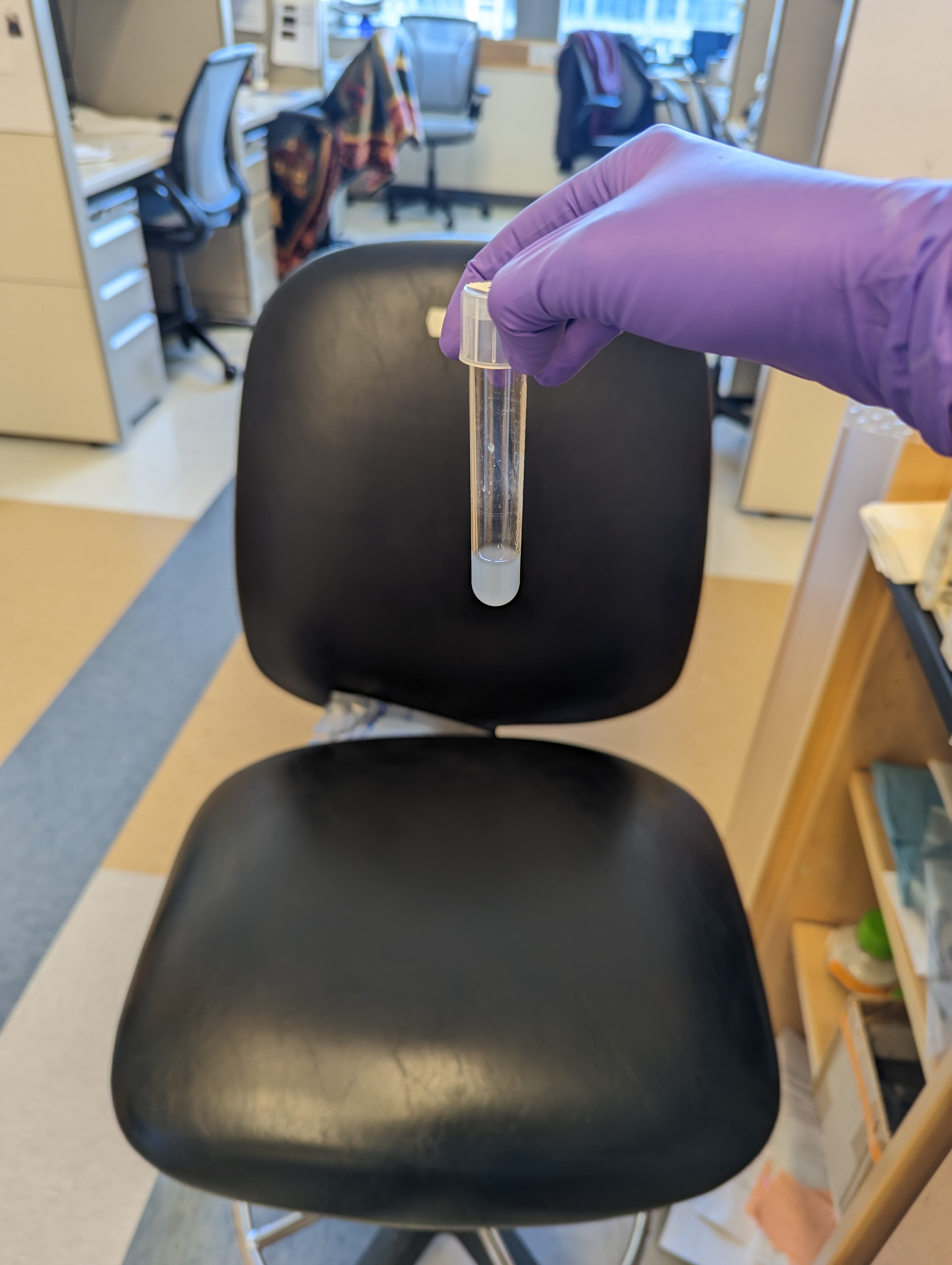

Supplement: S3 File — (ZIP) [file pgen.1011528.s009.zip › Fig 2B/2B frag kan dup- delta csuFABCDE,iou,bfmSR.jpg]

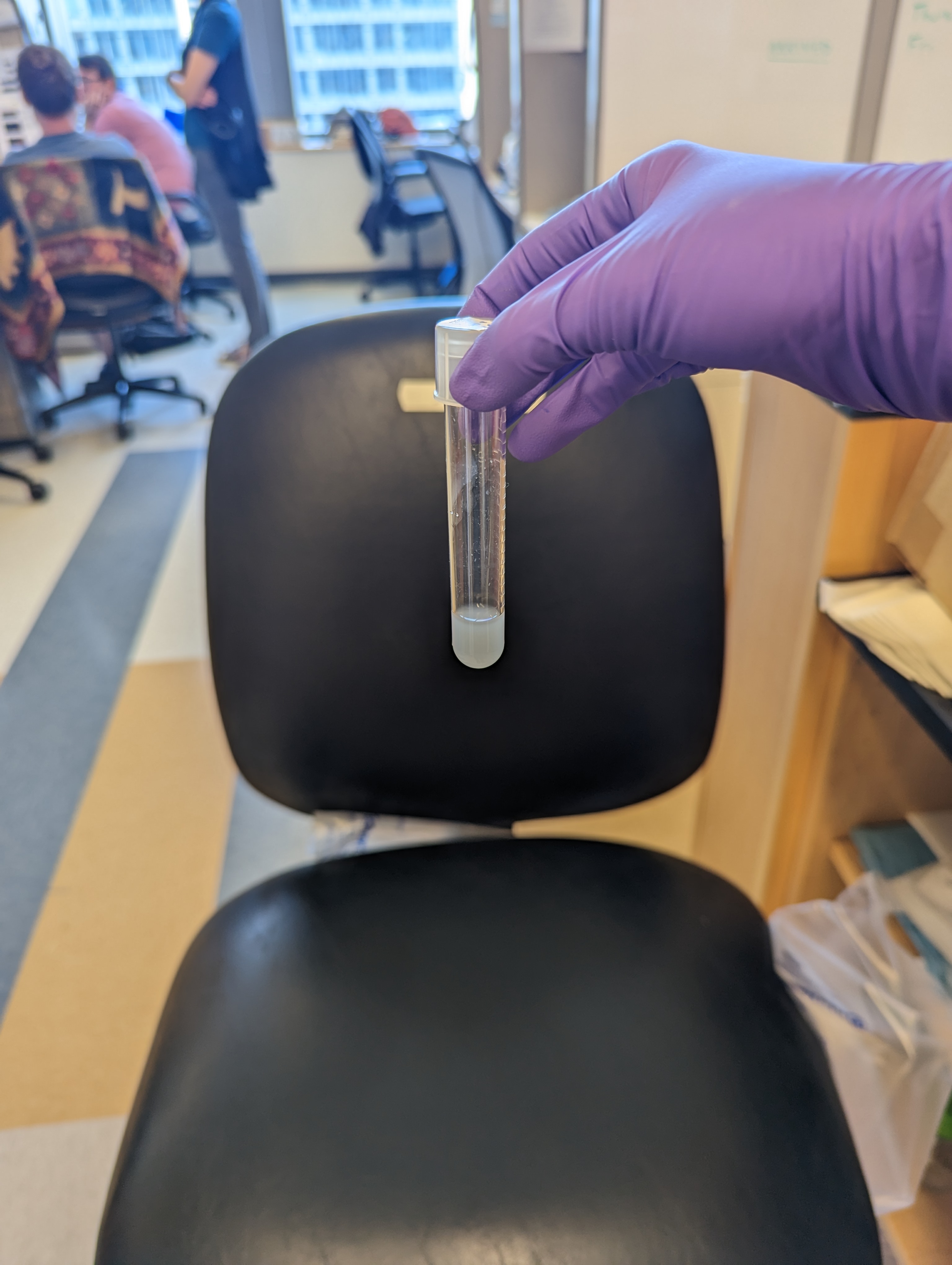

Supplement: S3 File — (ZIP) [file pgen.1011528.s009.zip › Fig 2B/2B frag kan dup- delta csuFABCDE.jpg]

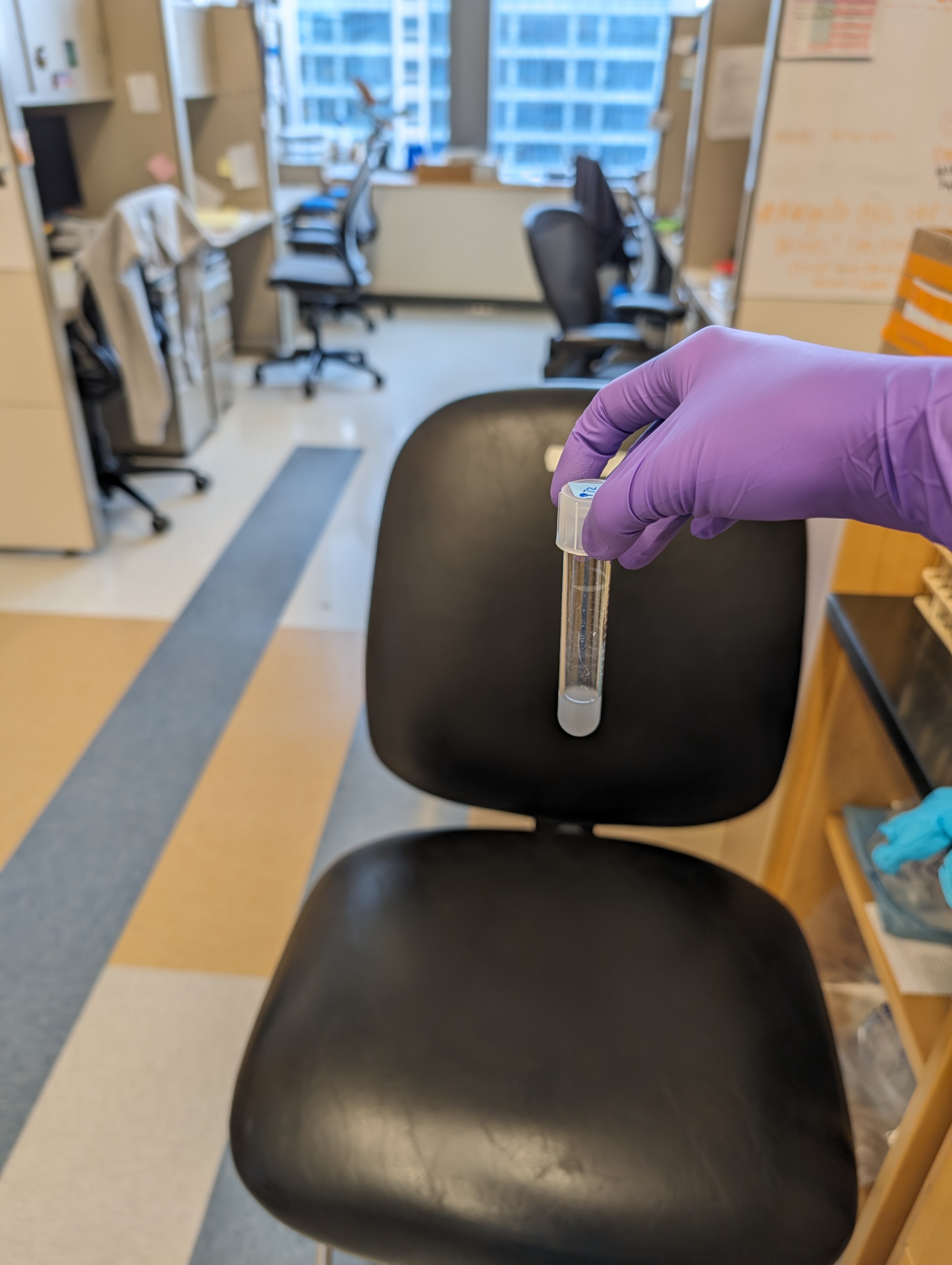

Supplement: S3 File — (ZIP) [file pgen.1011528.s009.zip › Fig 2B/2B frag kan dup- delta iou.jpg]

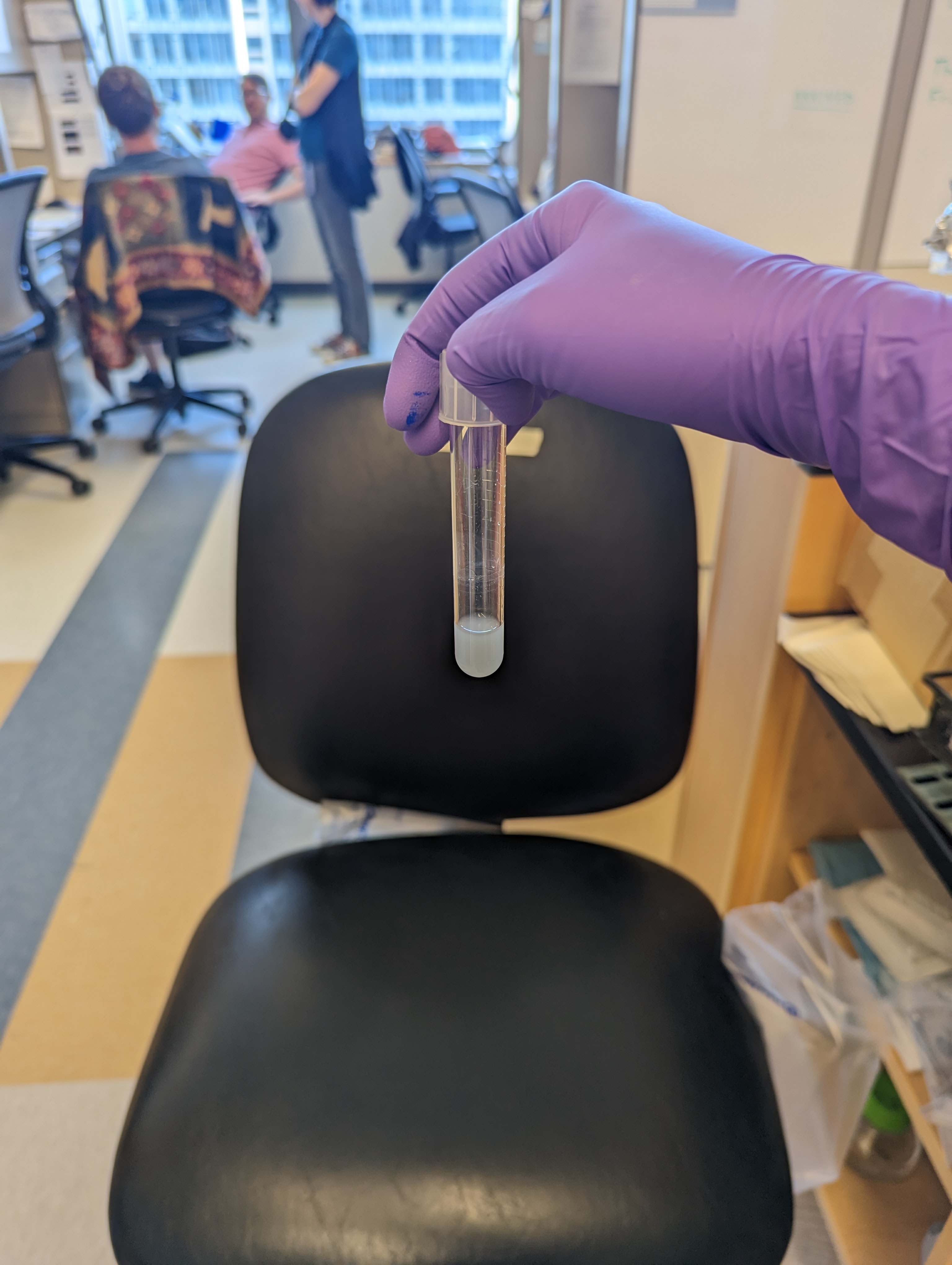

Supplement: S3 File — (ZIP) [file pgen.1011528.s009.zip › Fig 2B/2B frag kan dup- wildtype.jpg]

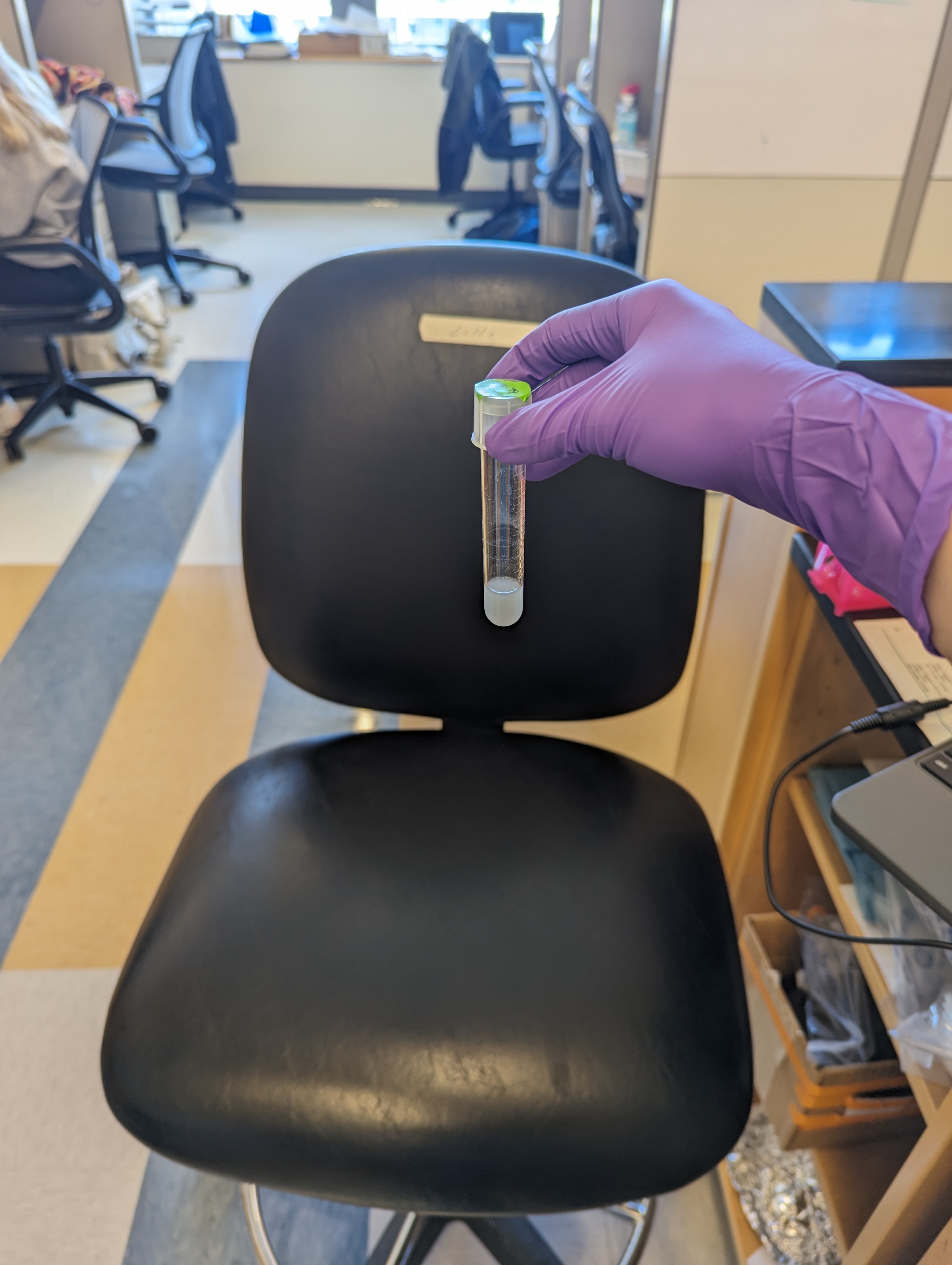

Supplement: S3 File — (ZIP) [file pgen.1011528.s009.zip › Fig 2B/2B frag kan dup+ delta bfmSR.jpg]

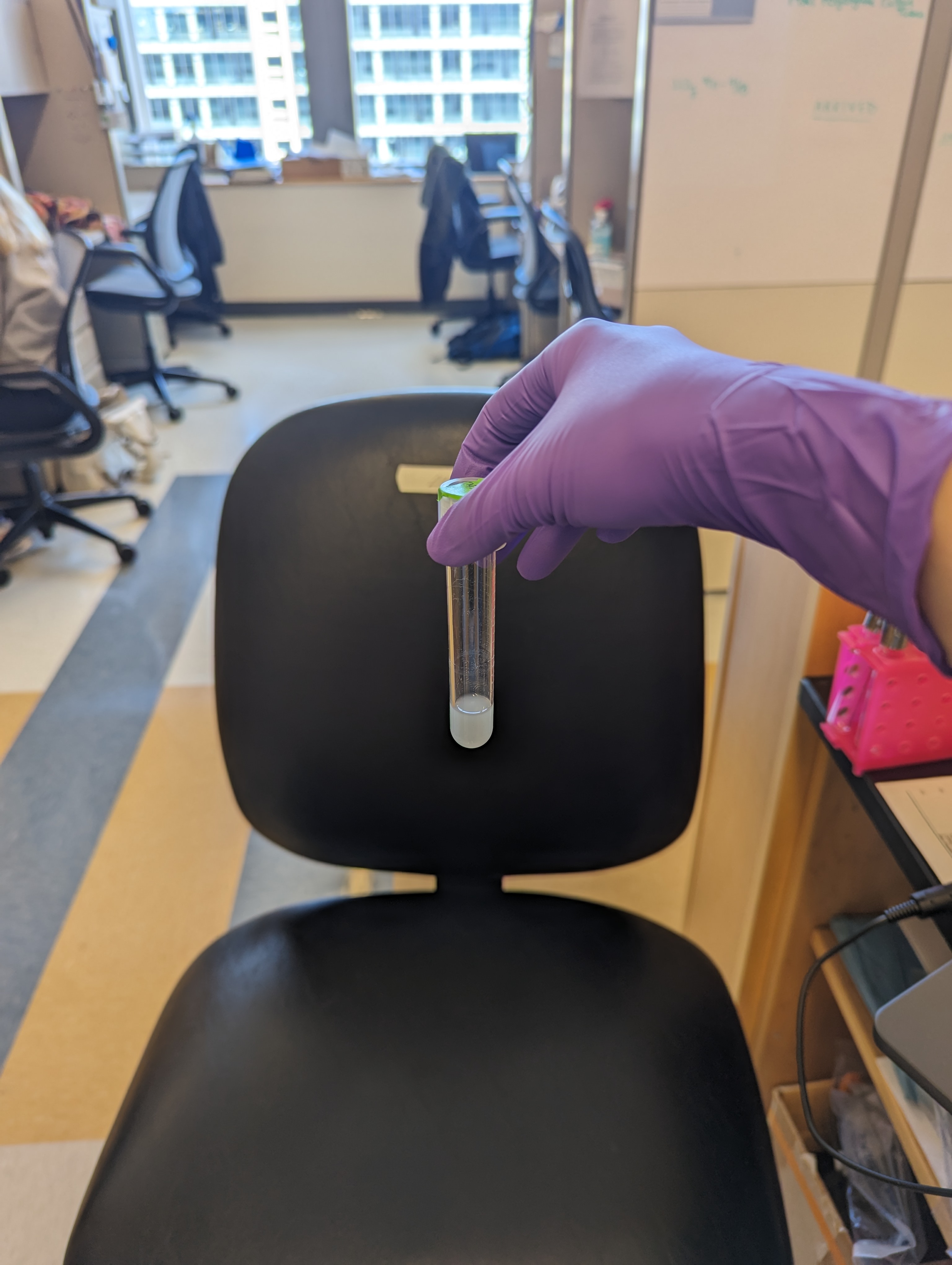

Supplement: S3 File — (ZIP) [file pgen.1011528.s009.zip › Fig 2B/2B frag kan dup+ delta csuFABCDE,iou,bfmSR.jpg]

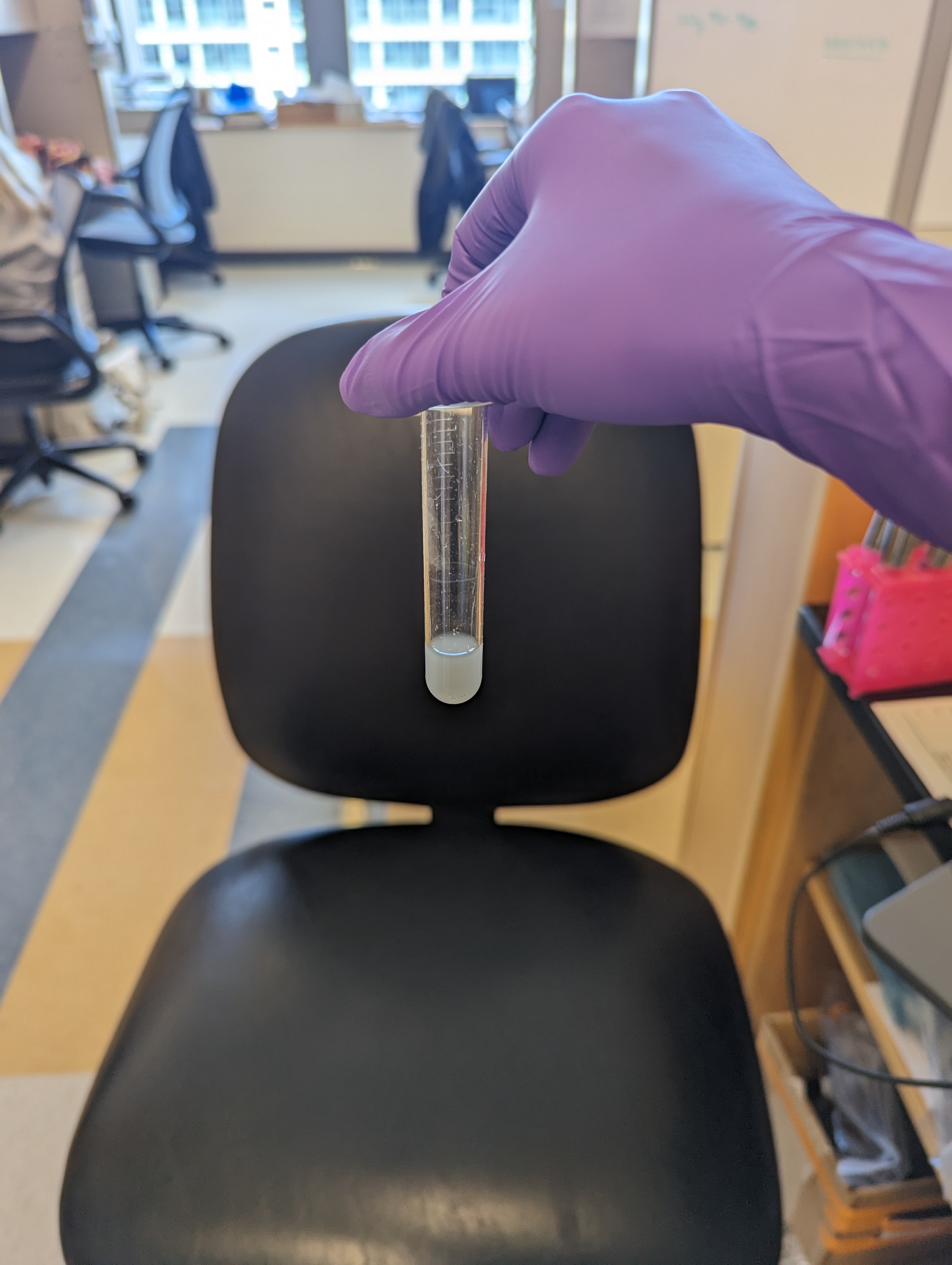

Supplement: S3 File — (ZIP) [file pgen.1011528.s009.zip › Fig 2B/2B frag kan dup+ delta csuFABCDE.jpg]

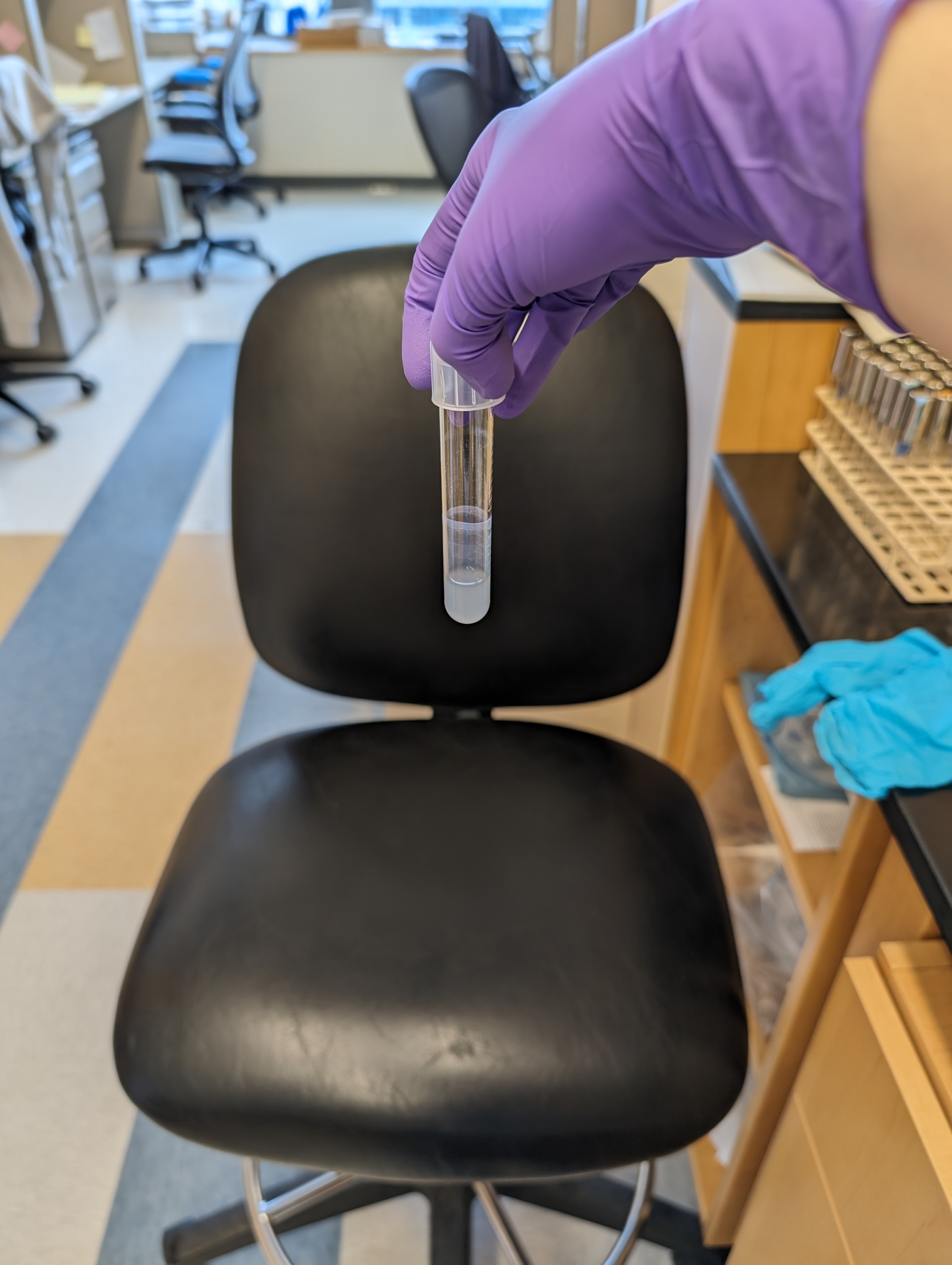

Supplement: S3 File — (ZIP) [file pgen.1011528.s009.zip › Fig 2B/2B frag kan dup+ delta iou.jpg]

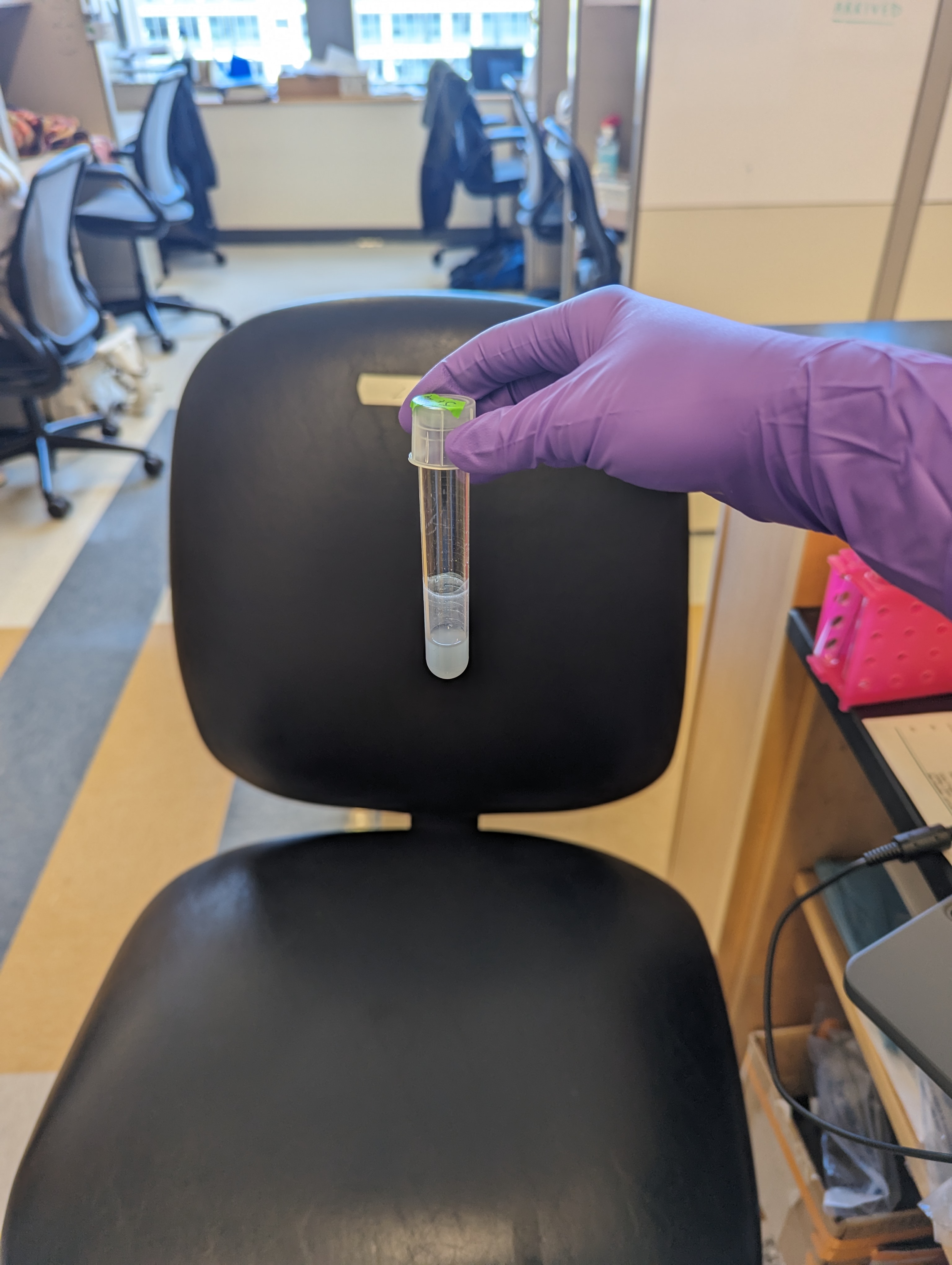

Supplement: S3 File — (ZIP) [file pgen.1011528.s009.zip › Fig 2B/2B frag kan dup+ wildtype.jpg]

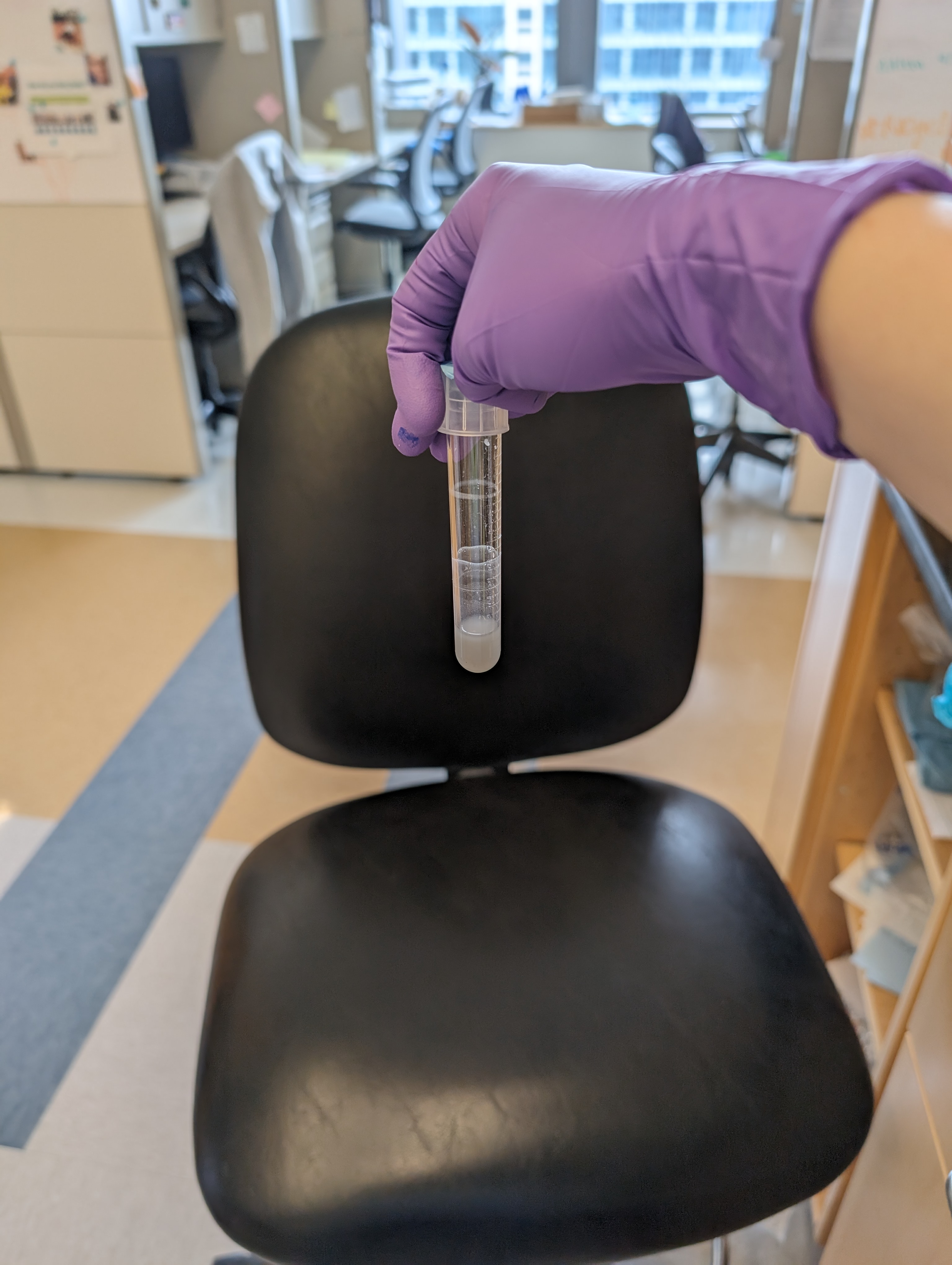

Supplement: S4 File — (ZIP) [file pgen.1011528.s010.zip › Fig 3A/3A reduced frag gus Day 2.jpg]

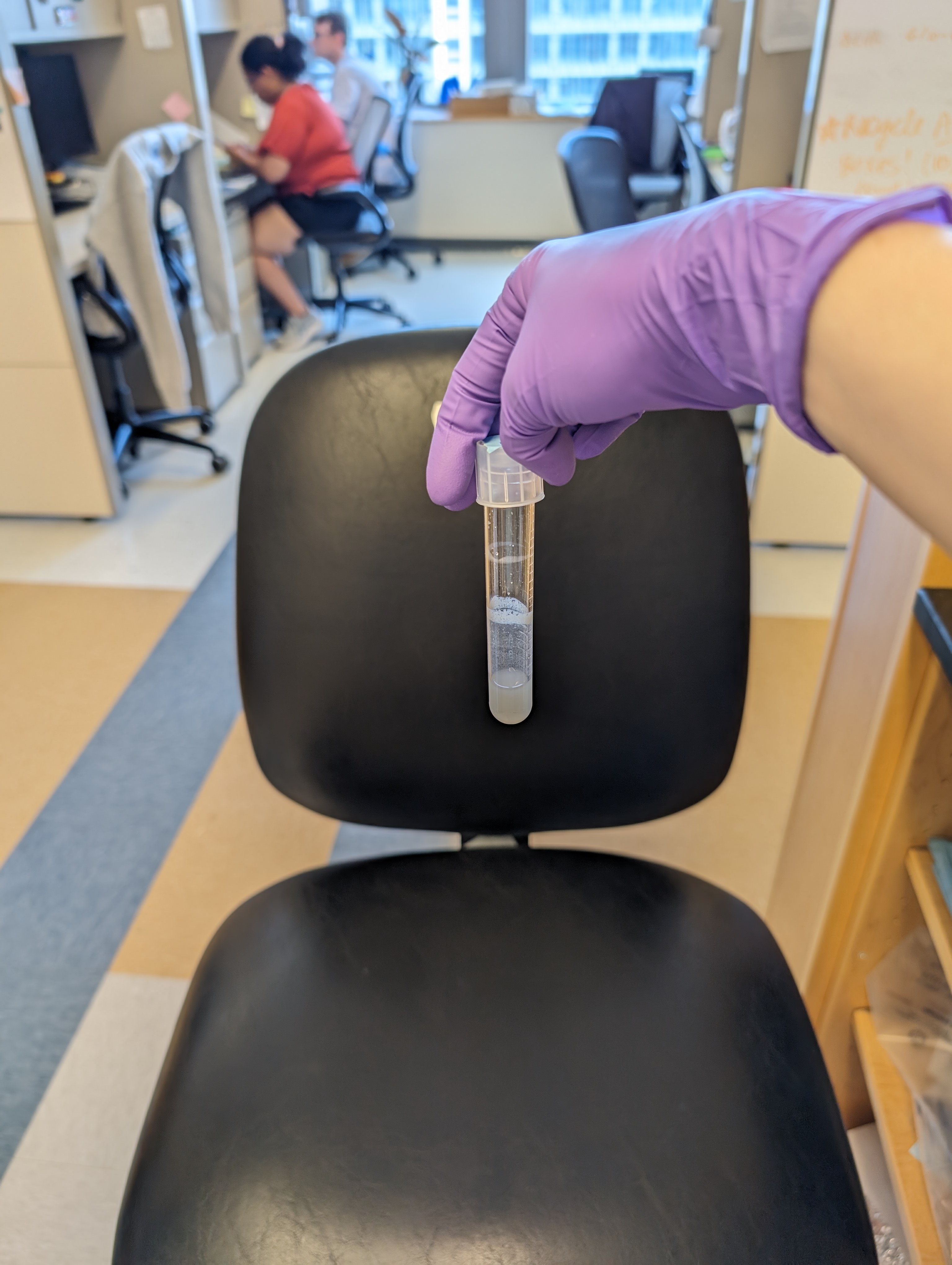

Supplement: S4 File — (ZIP) [file pgen.1011528.s010.zip › Fig 3A/3A reduced frag gus Day 4.jpg]

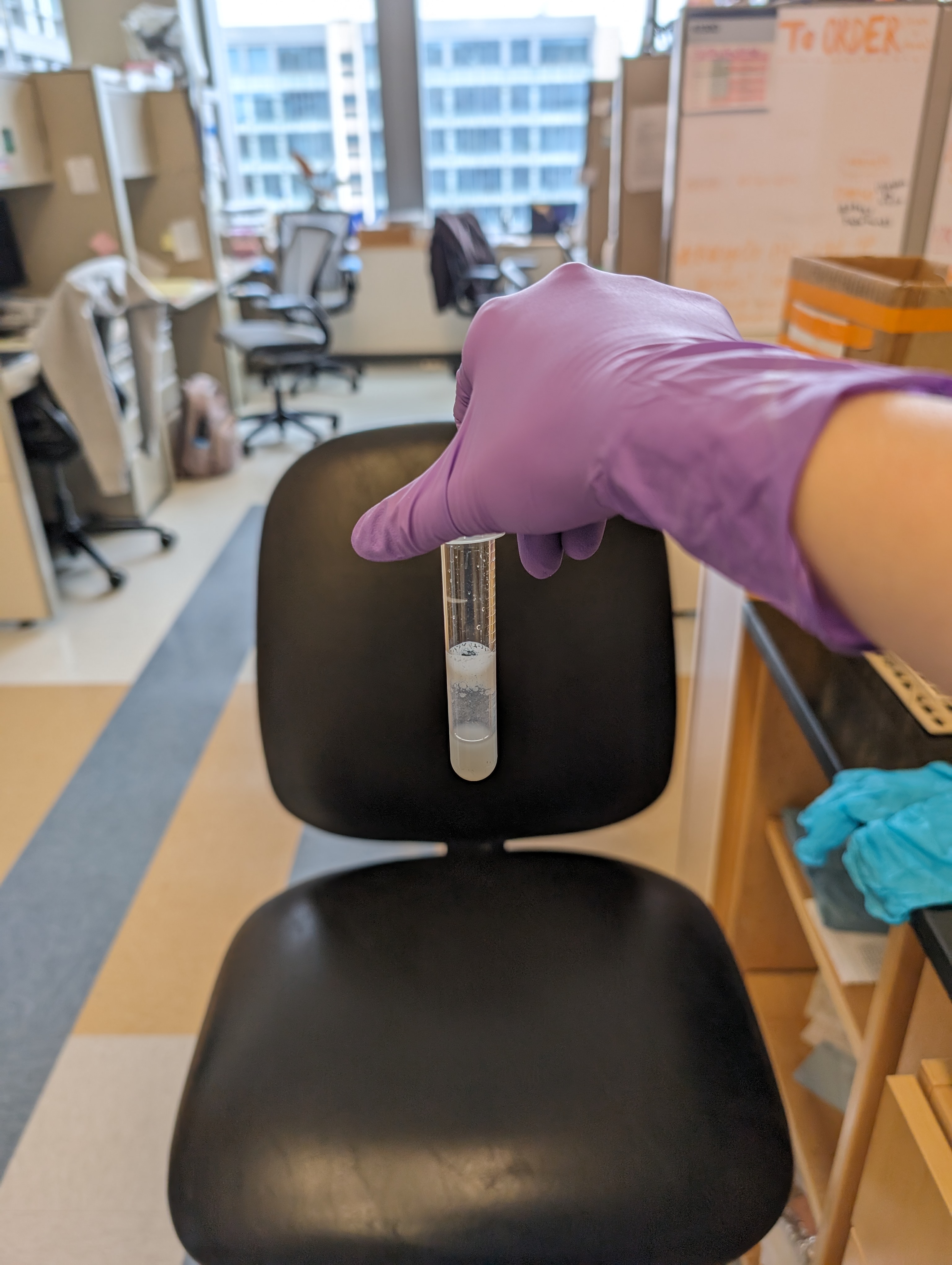

Supplement: S4 File — (ZIP) [file pgen.1011528.s010.zip › Fig 3A/3A reduced frag gus Day 6.jpg]

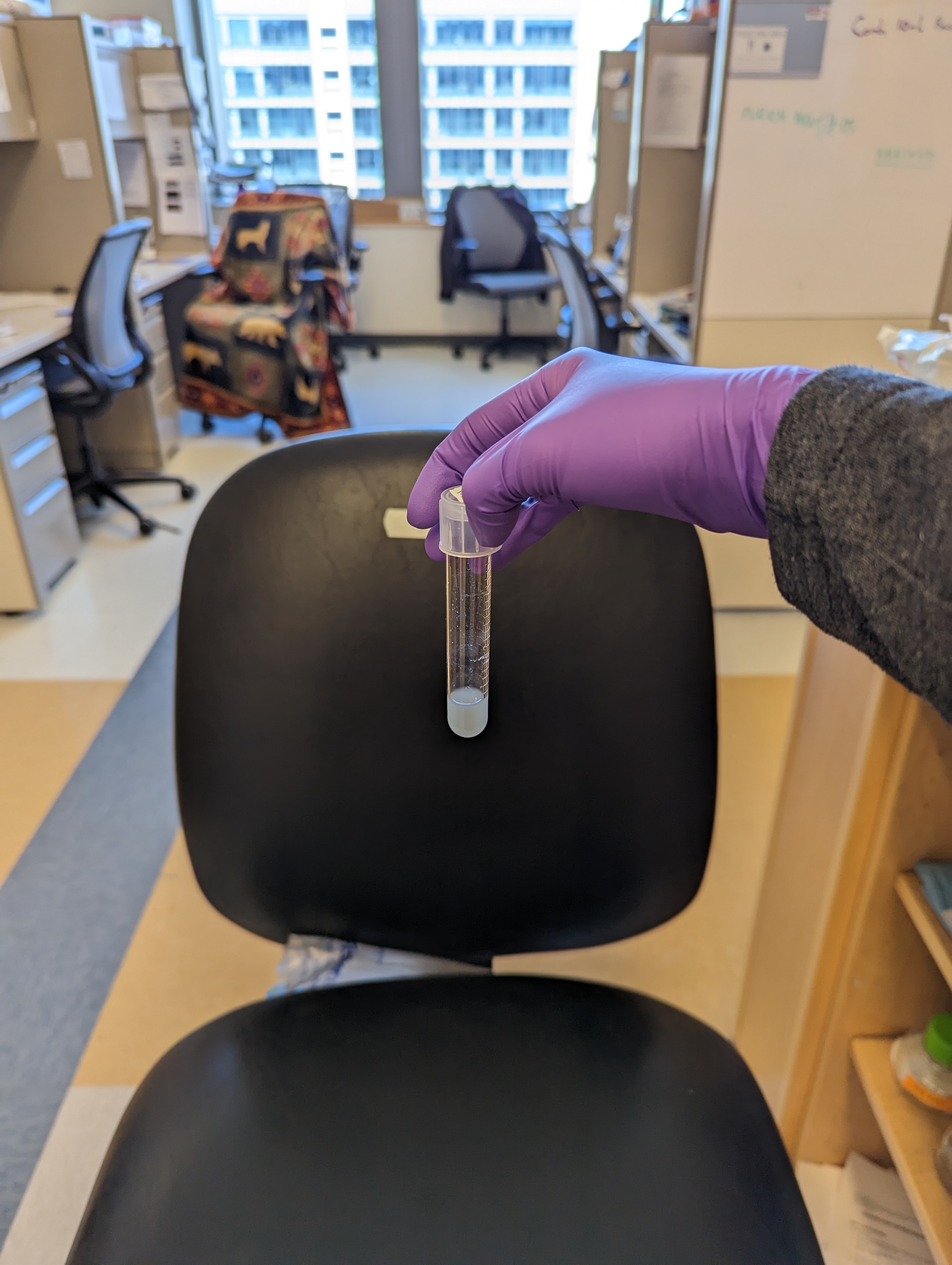

Supplement: S4 File — (ZIP) [file pgen.1011528.s010.zip › Fig 3A/3A standard frag gus delta bfmSR Day 2.jpg]

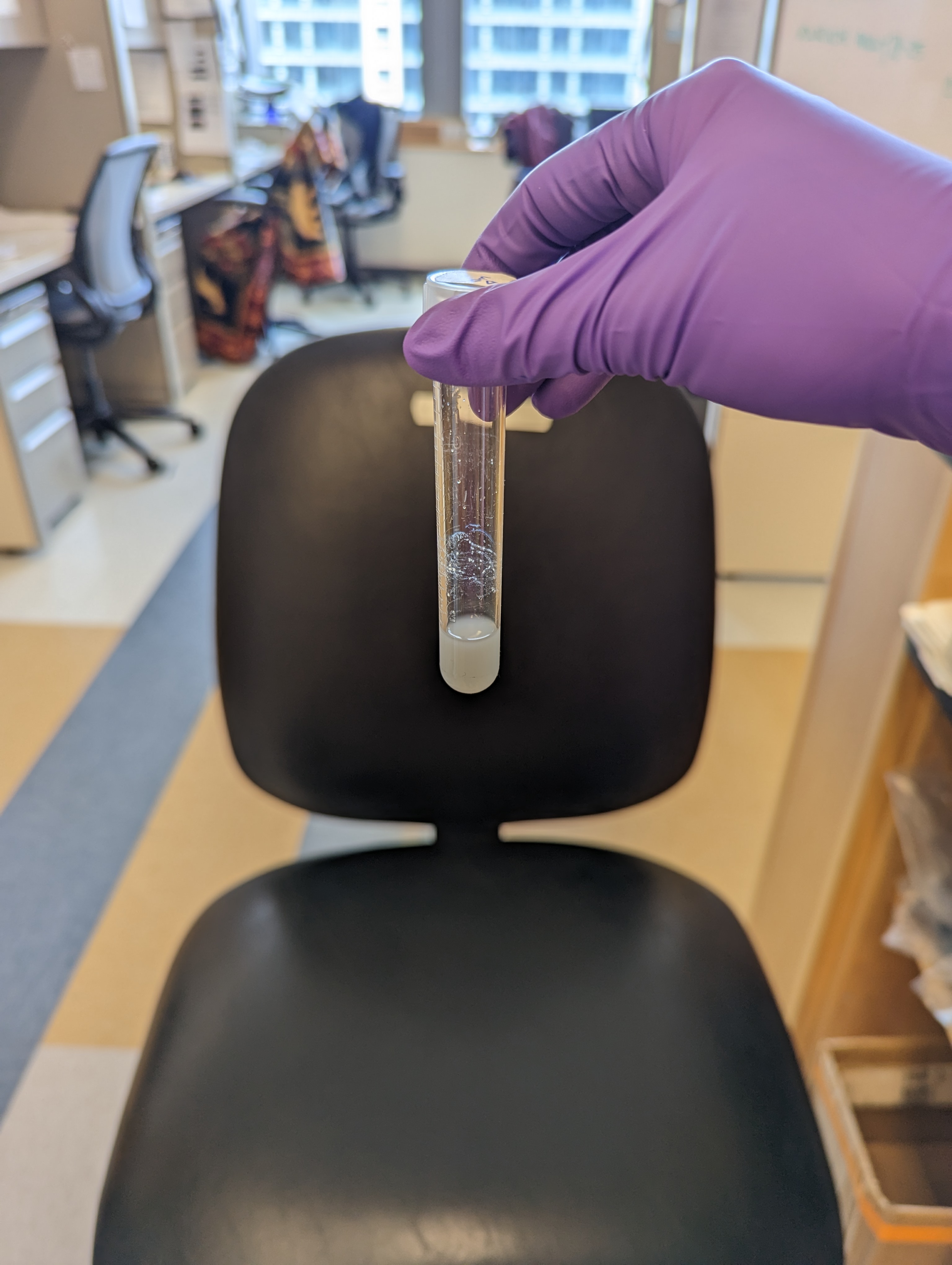

Supplement: S4 File — (ZIP) [file pgen.1011528.s010.zip › Fig 3A/3A standard frag gus delta bfmSR Day 4.jpg]

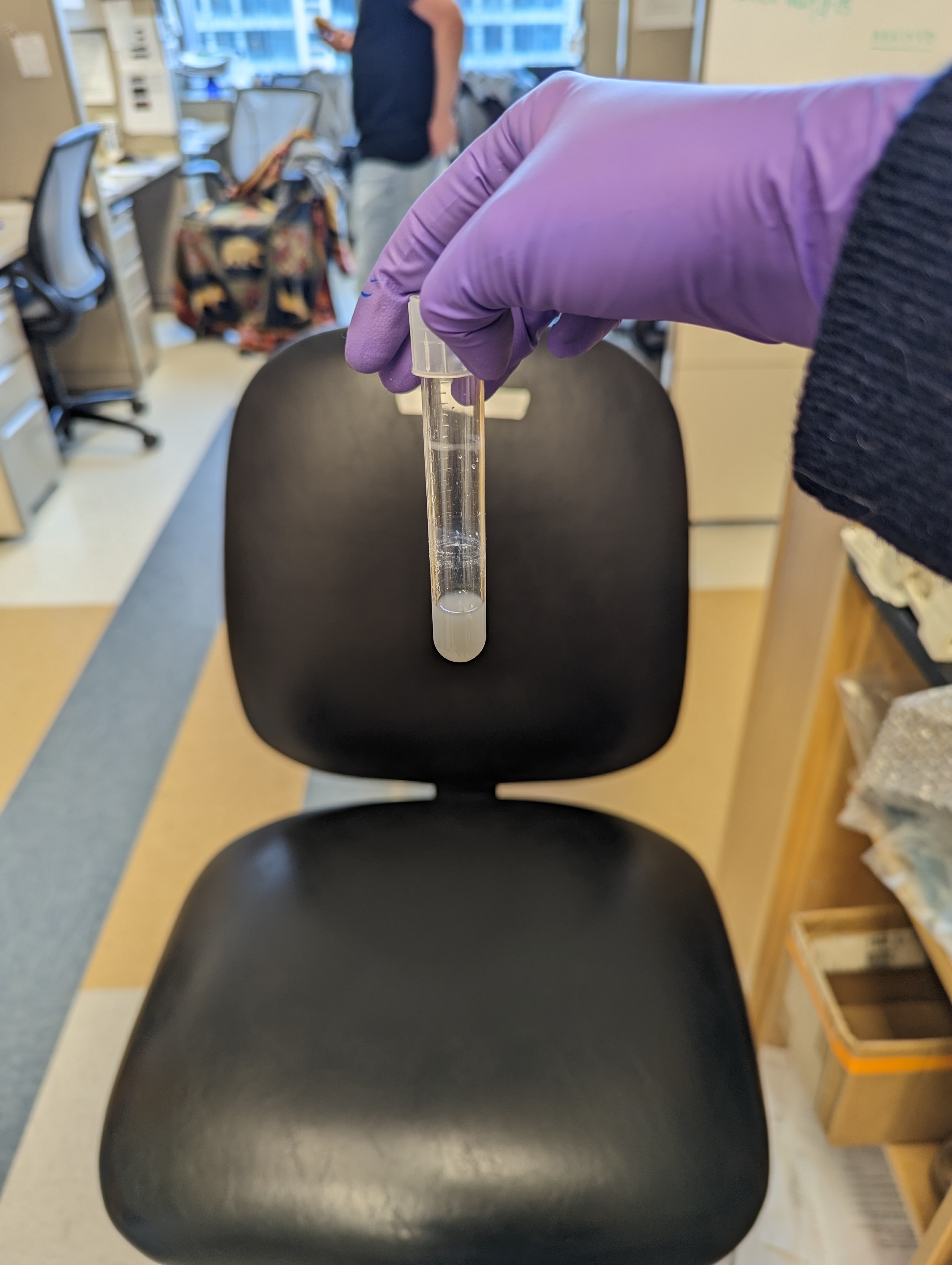

Supplement: S4 File — (ZIP) [file pgen.1011528.s010.zip › Fig 3A/3A standard frag gus delta bfmSR Day 6.jpg]

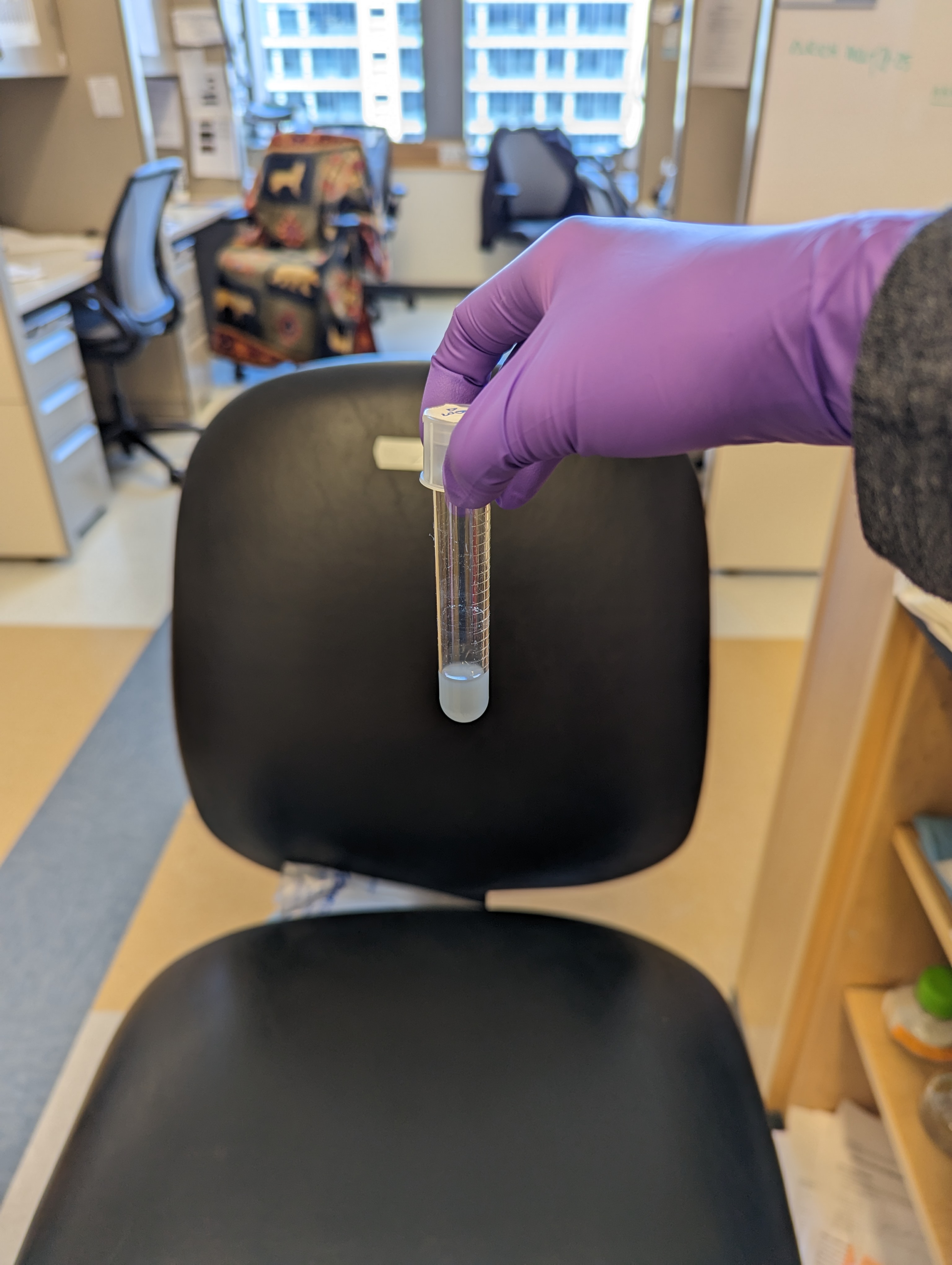

Supplement: S4 File — (ZIP) [file pgen.1011528.s010.zip › Fig 3A/3A standard frag gus delta csuFABCDE Day 2.jpg]

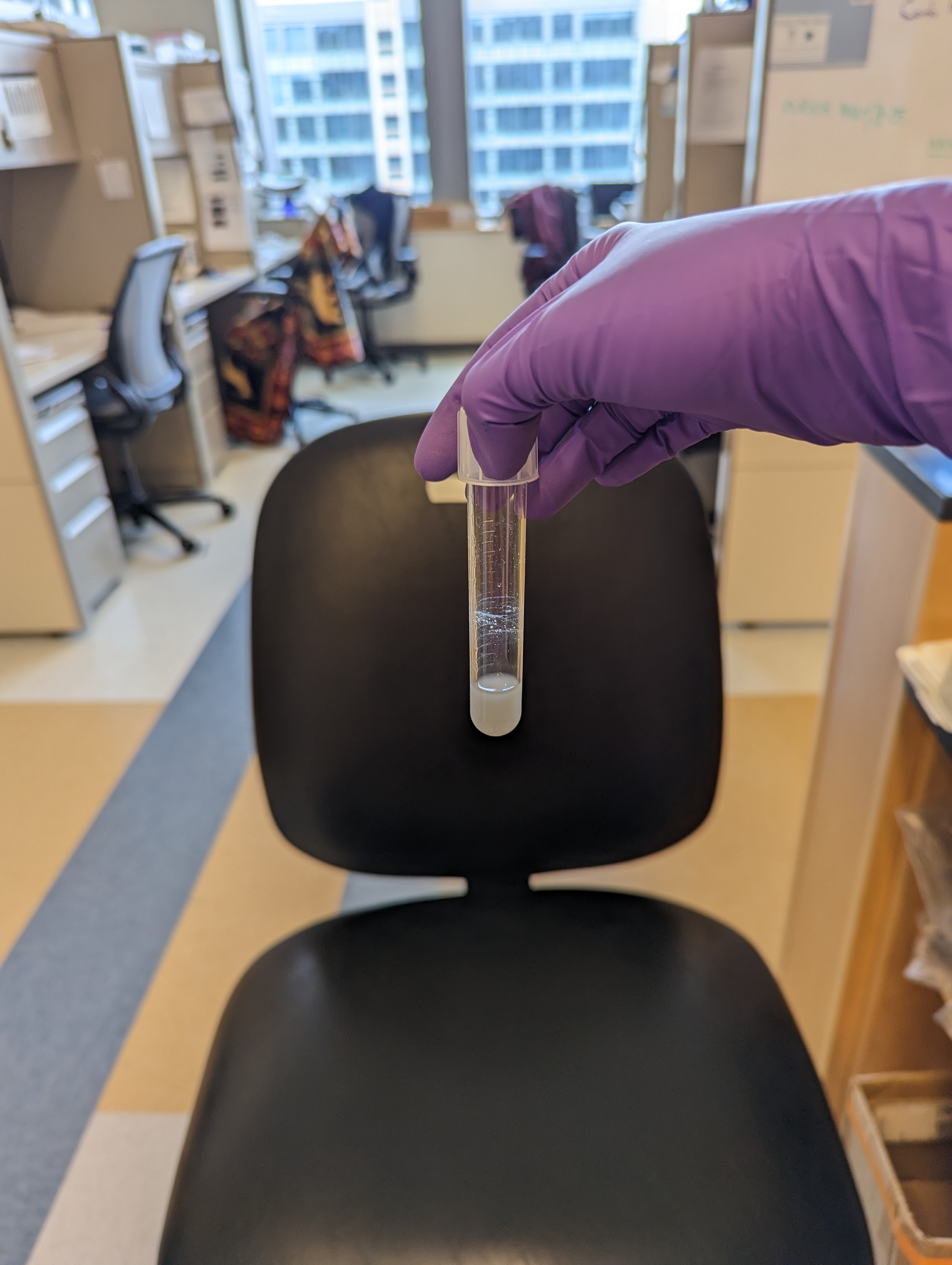

Supplement: S4 File — (ZIP) [file pgen.1011528.s010.zip › Fig 3A/3A standard frag gus delta csuFABCDE Day 4.jpg]

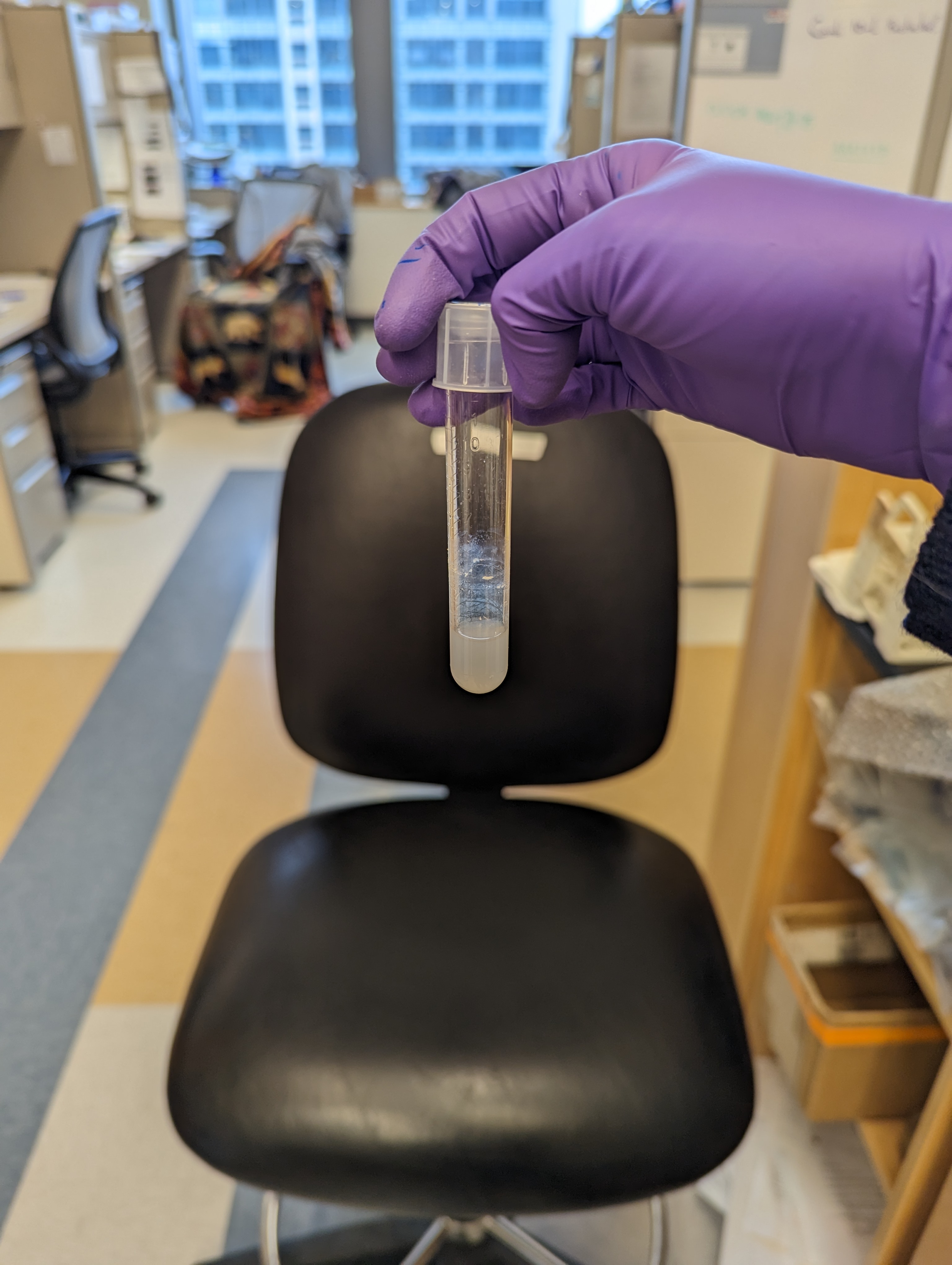

Supplement: S4 File — (ZIP) [file pgen.1011528.s010.zip › Fig 3A/3A standard frag gus delta csuFABCDE Day 6.jpg]

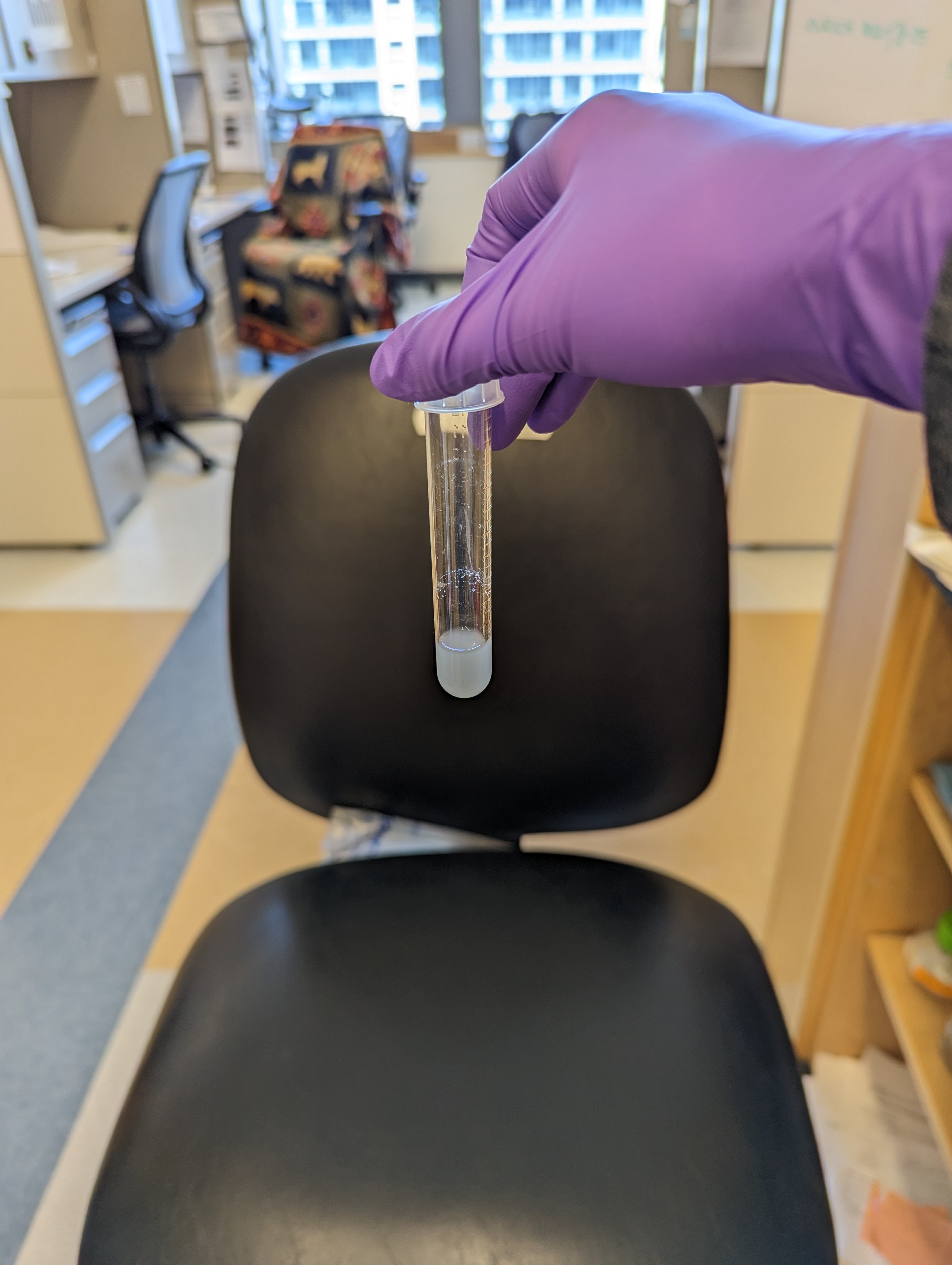

Supplement: S4 File — (ZIP) [file pgen.1011528.s010.zip › Fig 3A/3A standard frag gus delta csuFABCDE,iou,bfmSR Day 2.jpg]

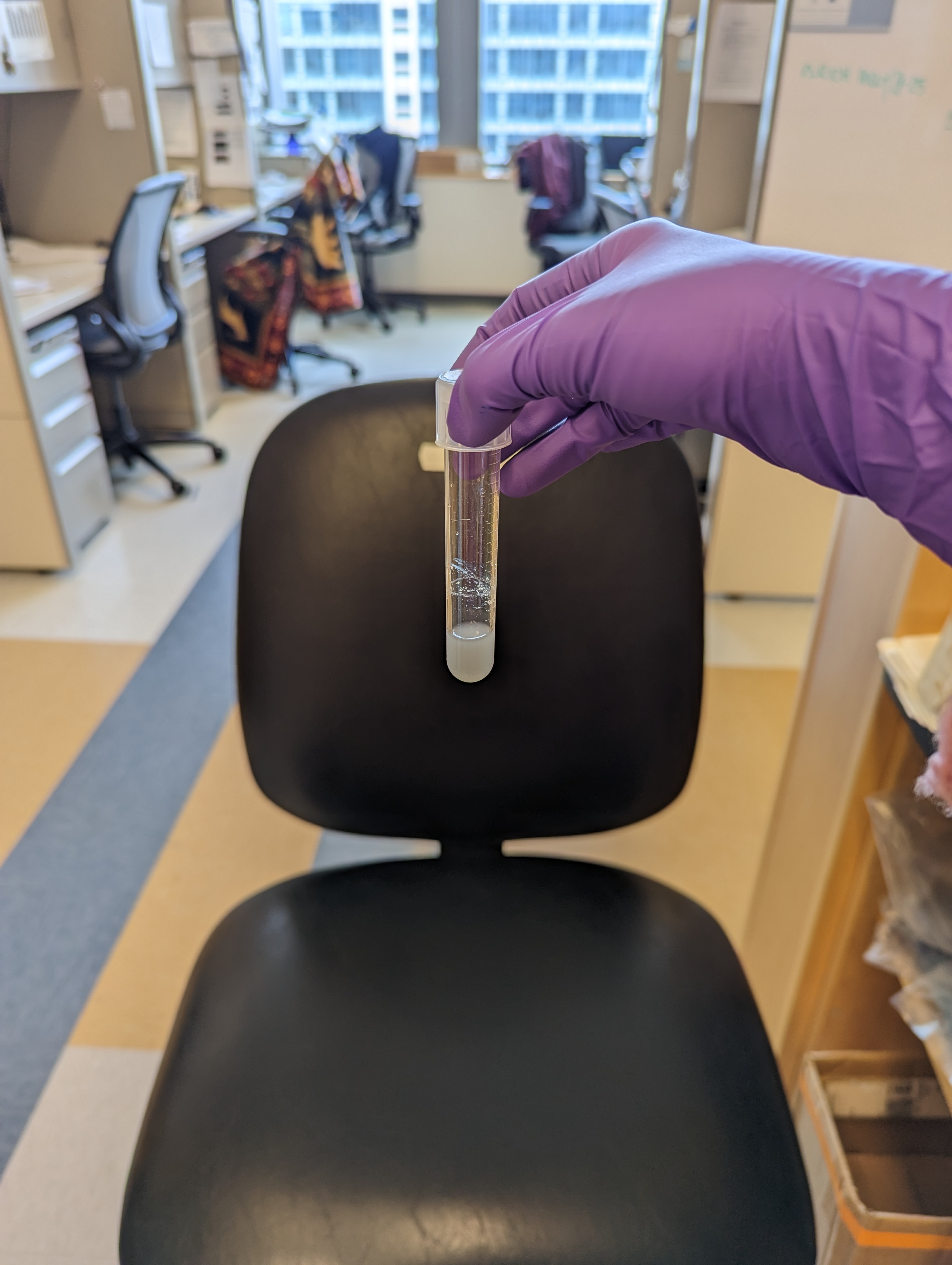

Supplement: S4 File — (ZIP) [file pgen.1011528.s010.zip › Fig 3A/3A standard frag gus delta csuFABCDE,iou,bfmSR Day 4.jpg]

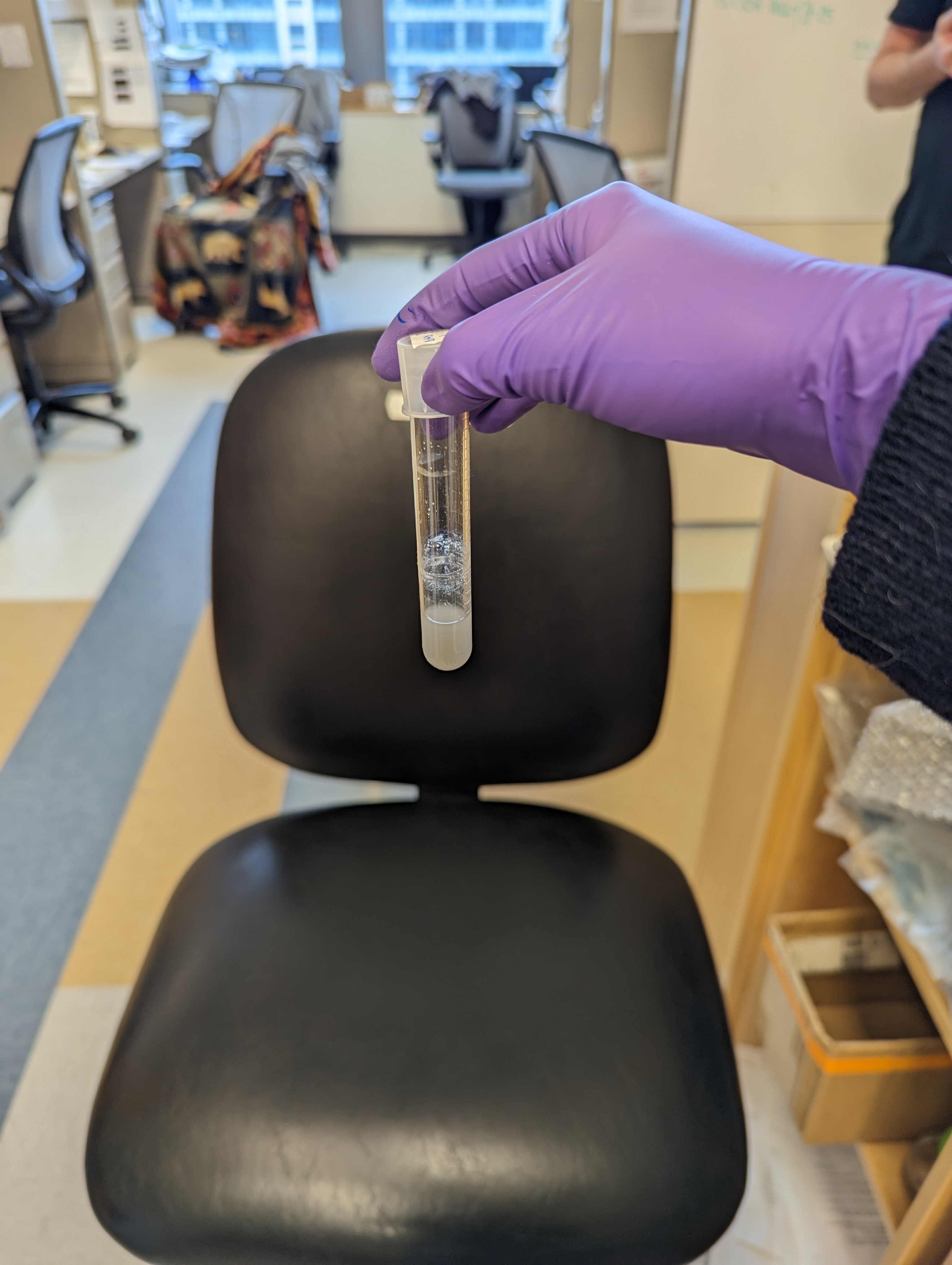

Supplement: S4 File — (ZIP) [file pgen.1011528.s010.zip › Fig 3A/3A standard frag gus delta csuFABCDE,iou,bfmSR Day 6.jpg]

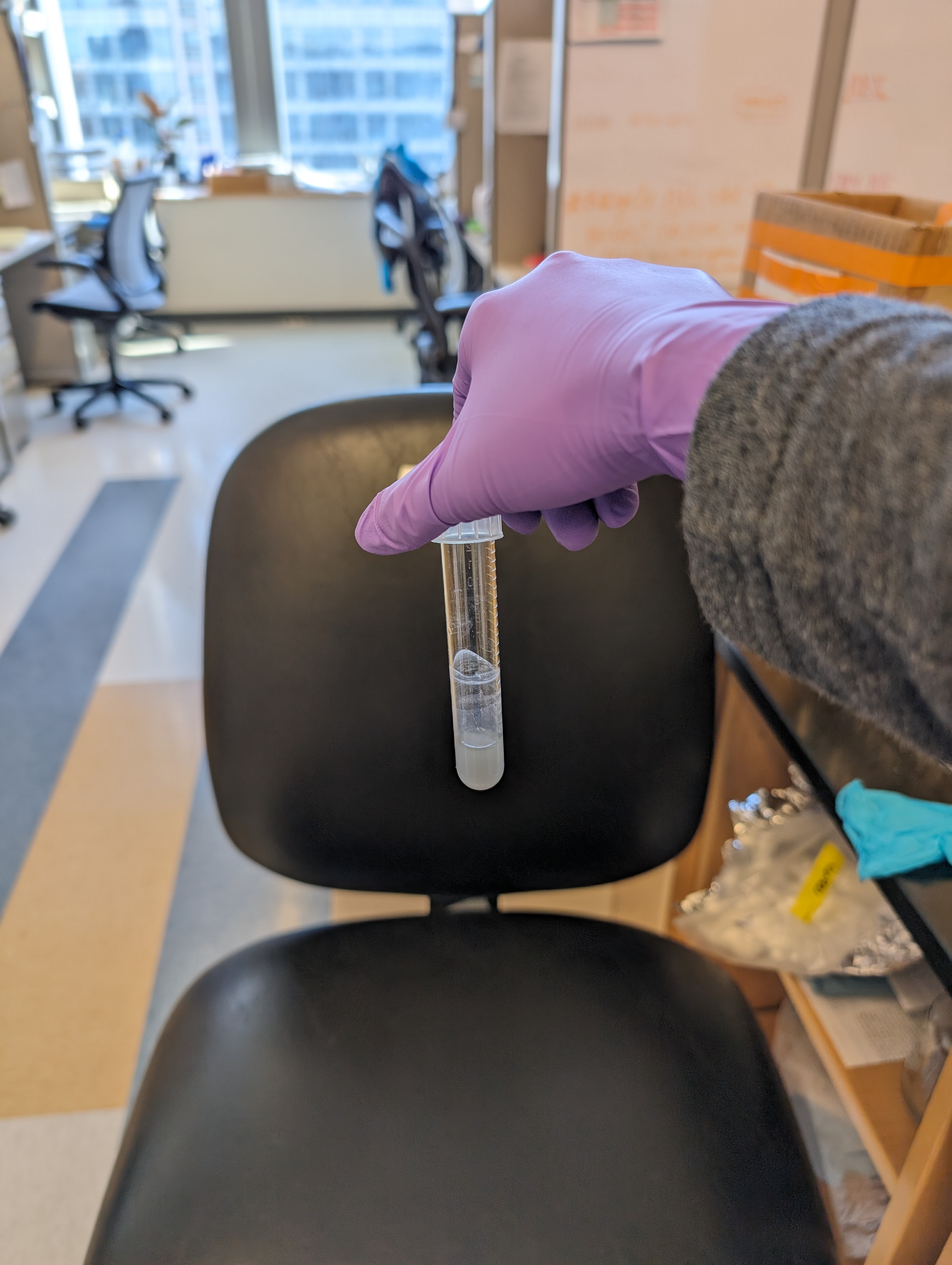

Supplement: S4 File — (ZIP) [file pgen.1011528.s010.zip › Fig 3A/3A standard frag gus delta iou Day 2.jpg]

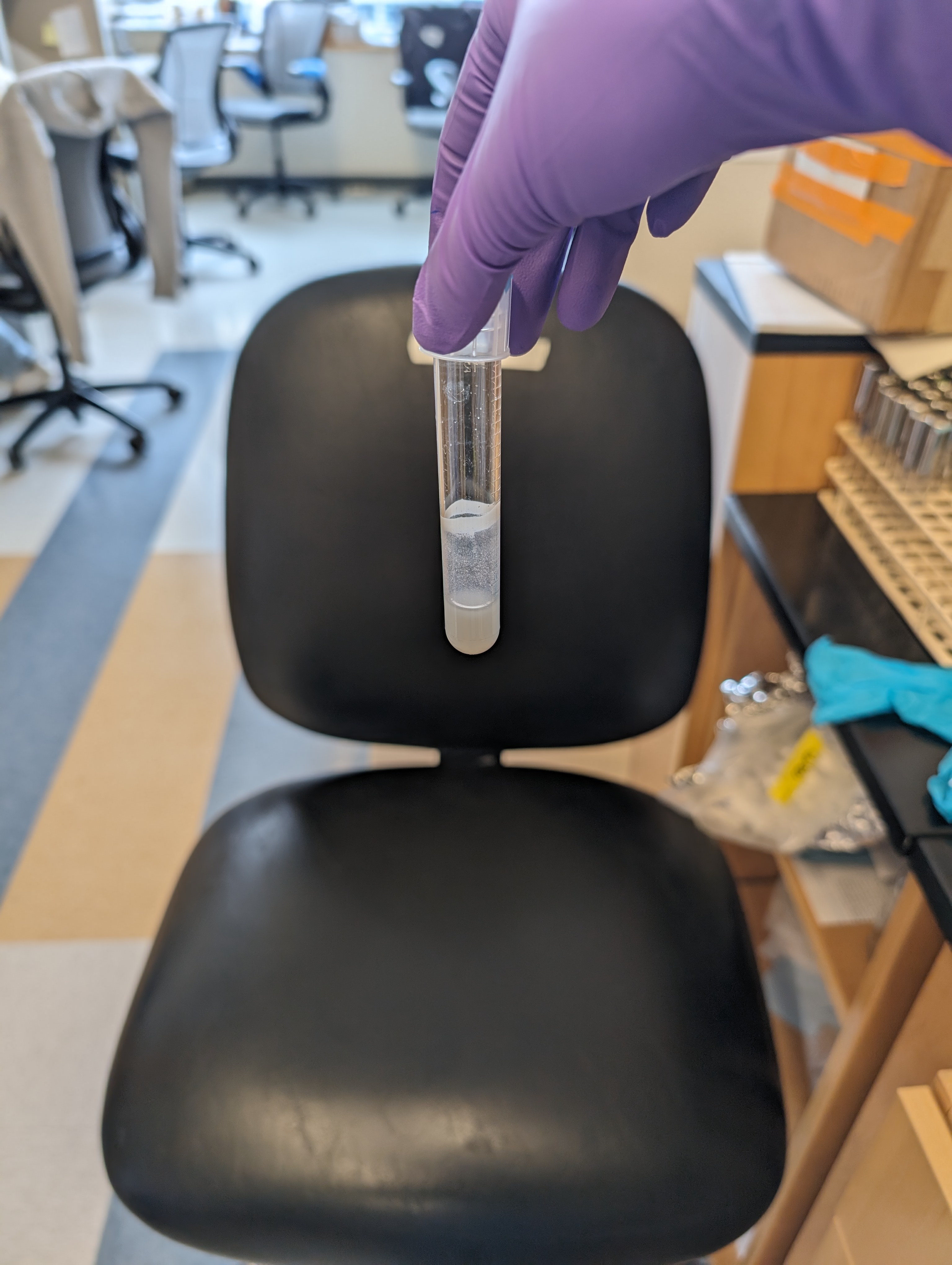

Supplement: S4 File — (ZIP) [file pgen.1011528.s010.zip › Fig 3A/3A standard frag gus delta iou Day 4.jpg]

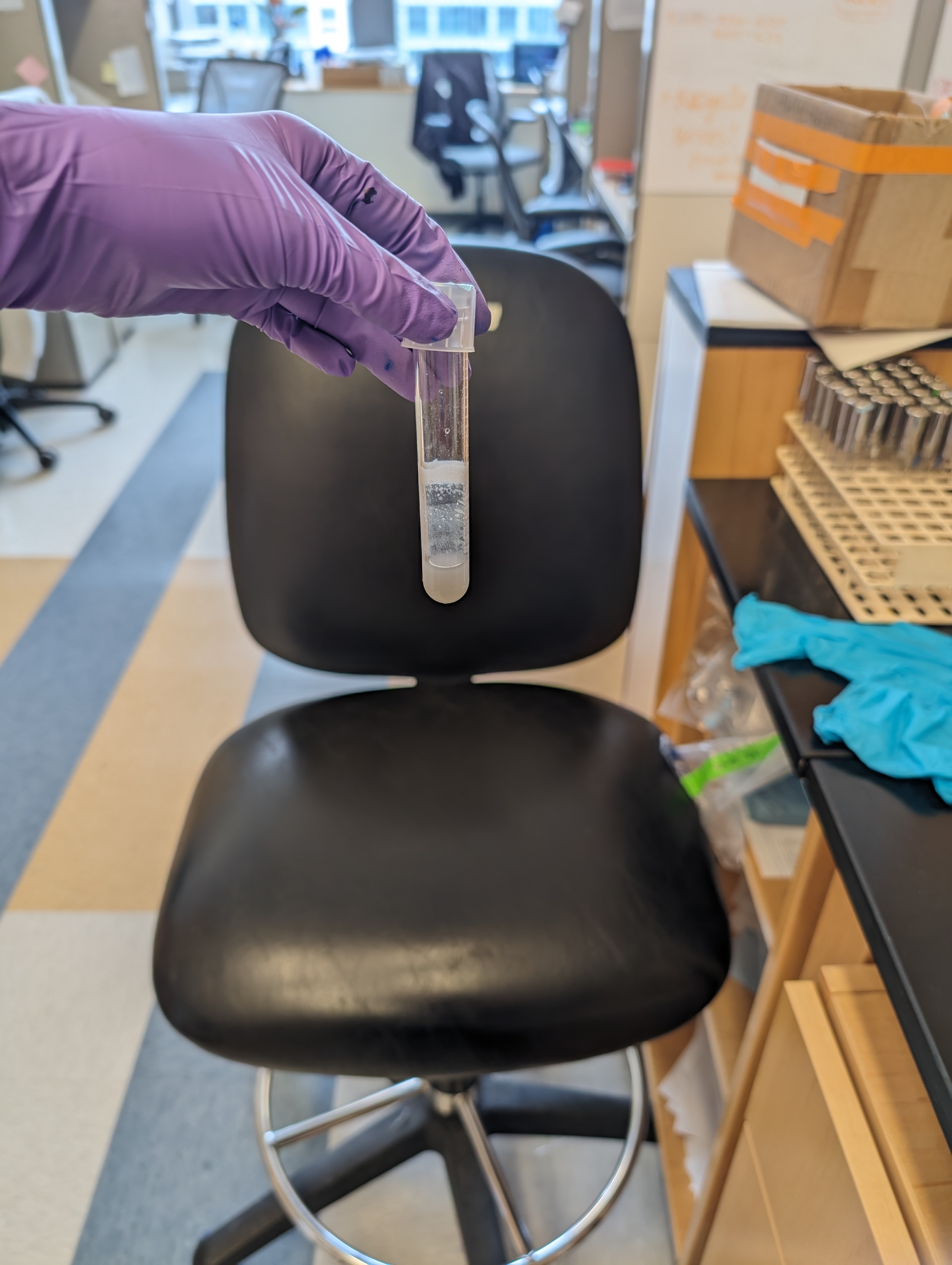

Supplement: S4 File — (ZIP) [file pgen.1011528.s010.zip › Fig 3A/3A standard frag gus delta iou Day 6.jpg]

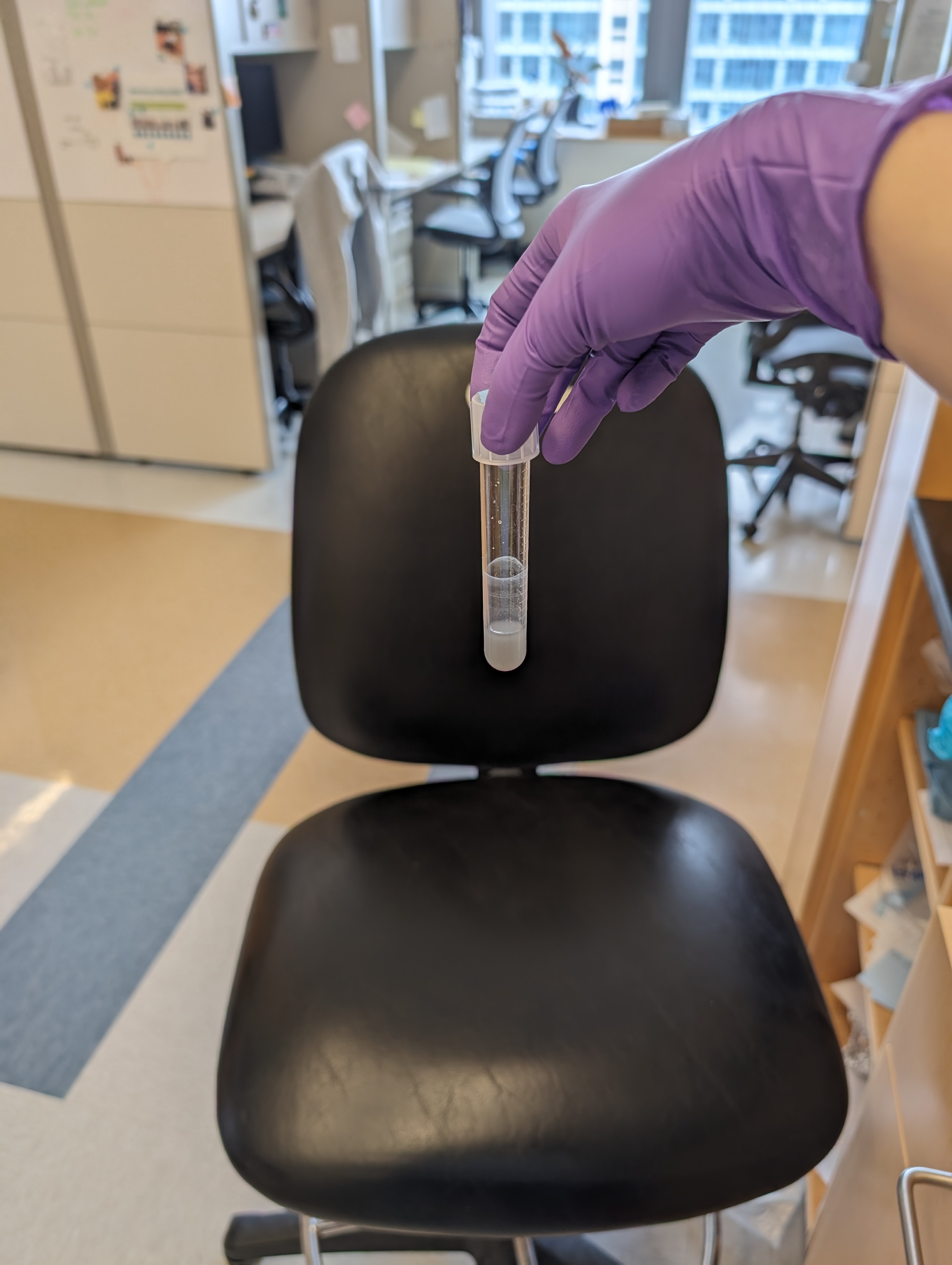

Supplement: S4 File — (ZIP) [file pgen.1011528.s010.zip › Fig 3A/3A standard frag gus wildtype Day 2.jpg]

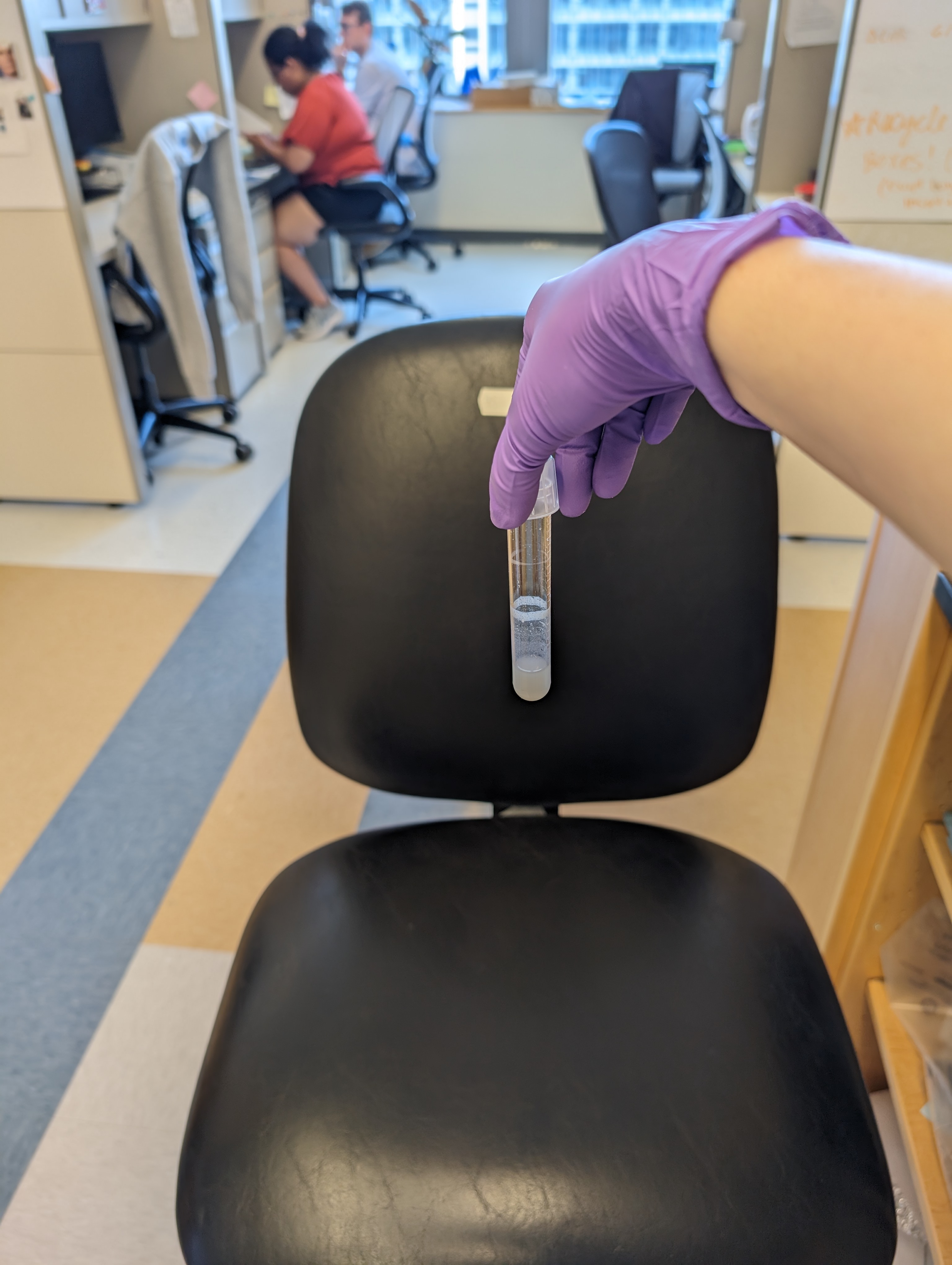

Supplement: S4 File — (ZIP) [file pgen.1011528.s010.zip › Fig 3A/3A standard frag gus wildtype Day 4.jpg]

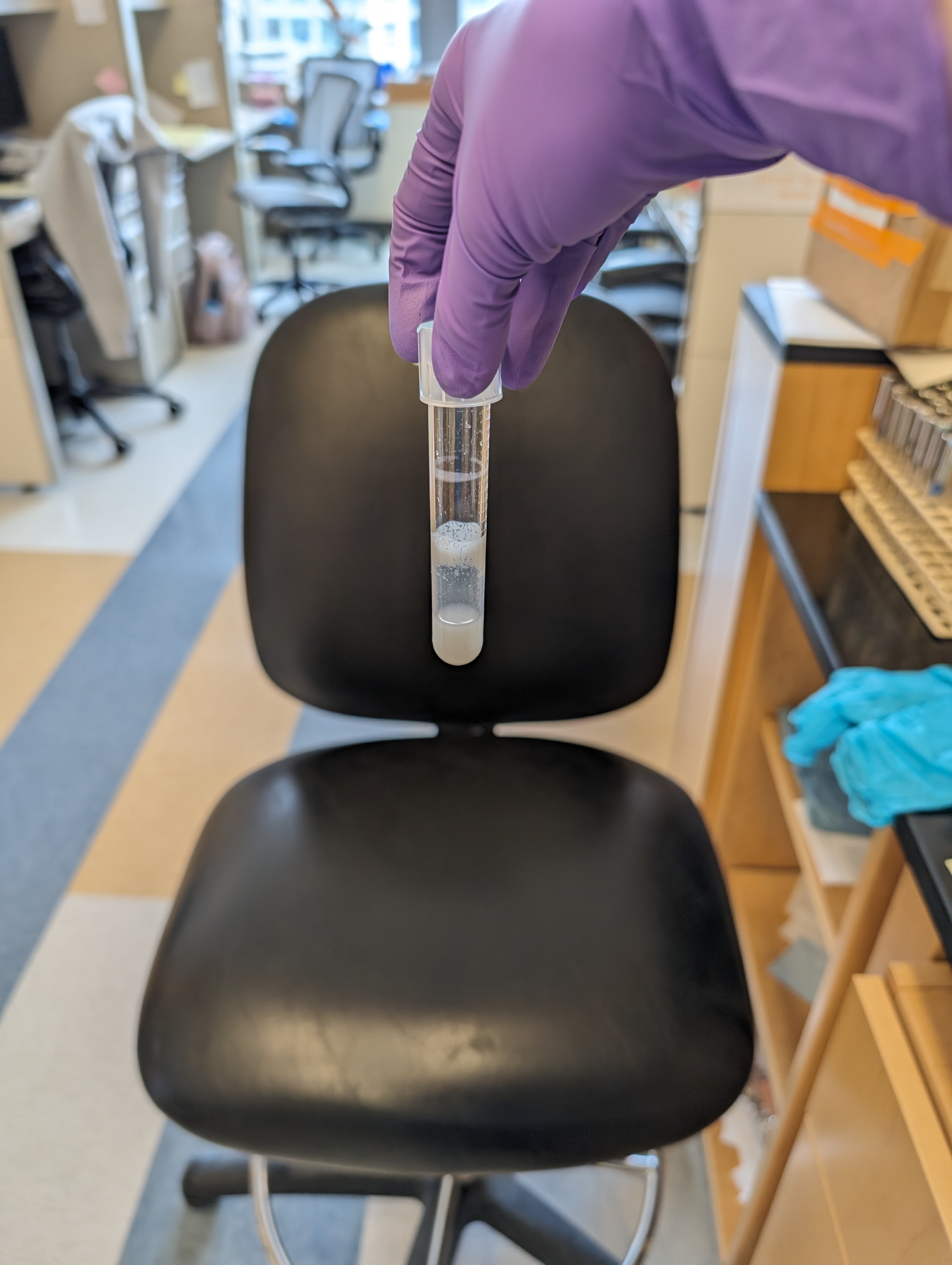

Supplement: S4 File — (ZIP) [file pgen.1011528.s010.zip › Fig 3A/3A standard frag gus wildtype Day 6.jpg]

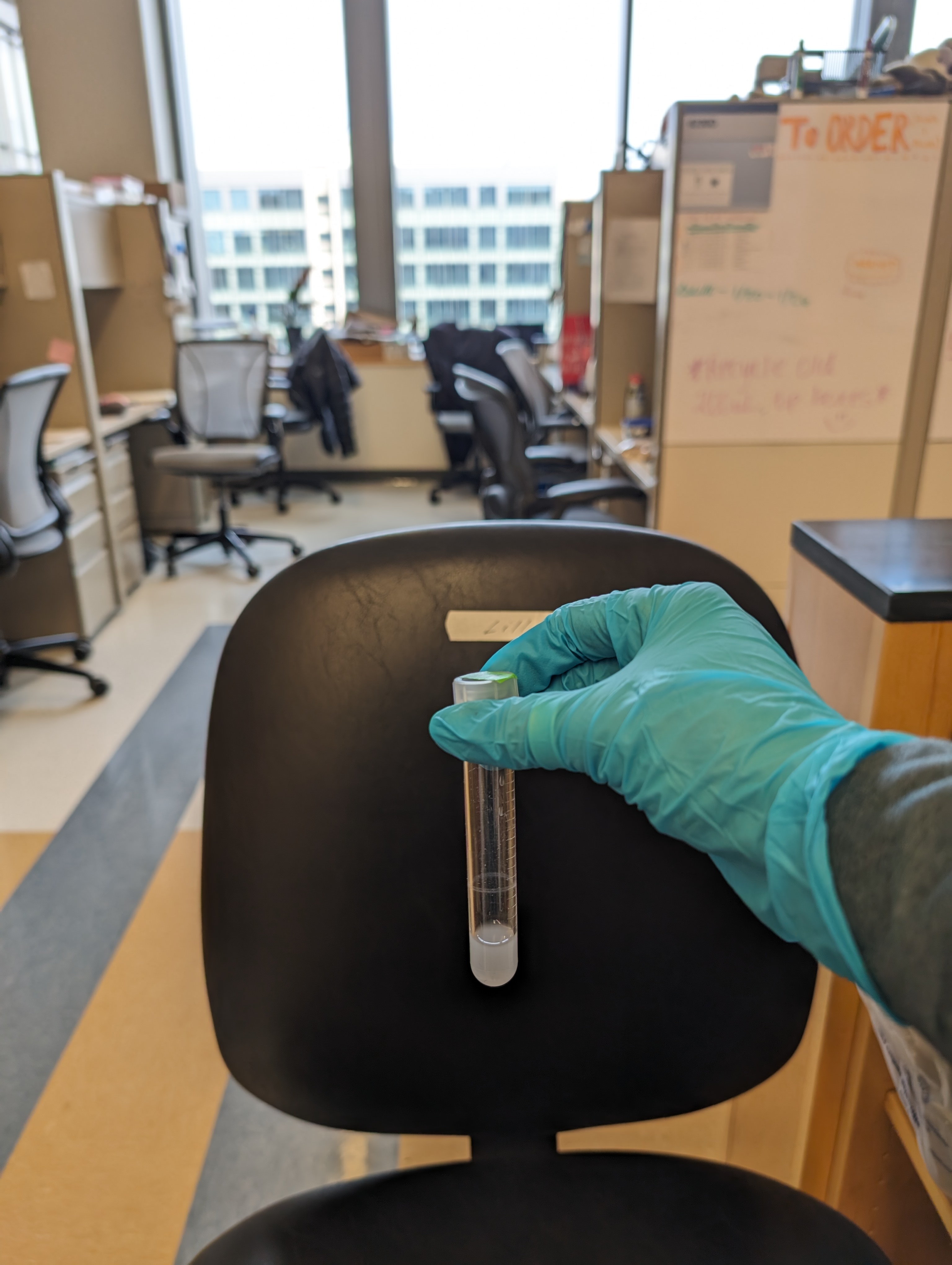

Supplement: S5 File — (ZIP) [file pgen.1011528.s011.zip › Fig 4D/4D frag kan dup- BfmR(D55A).jpg]

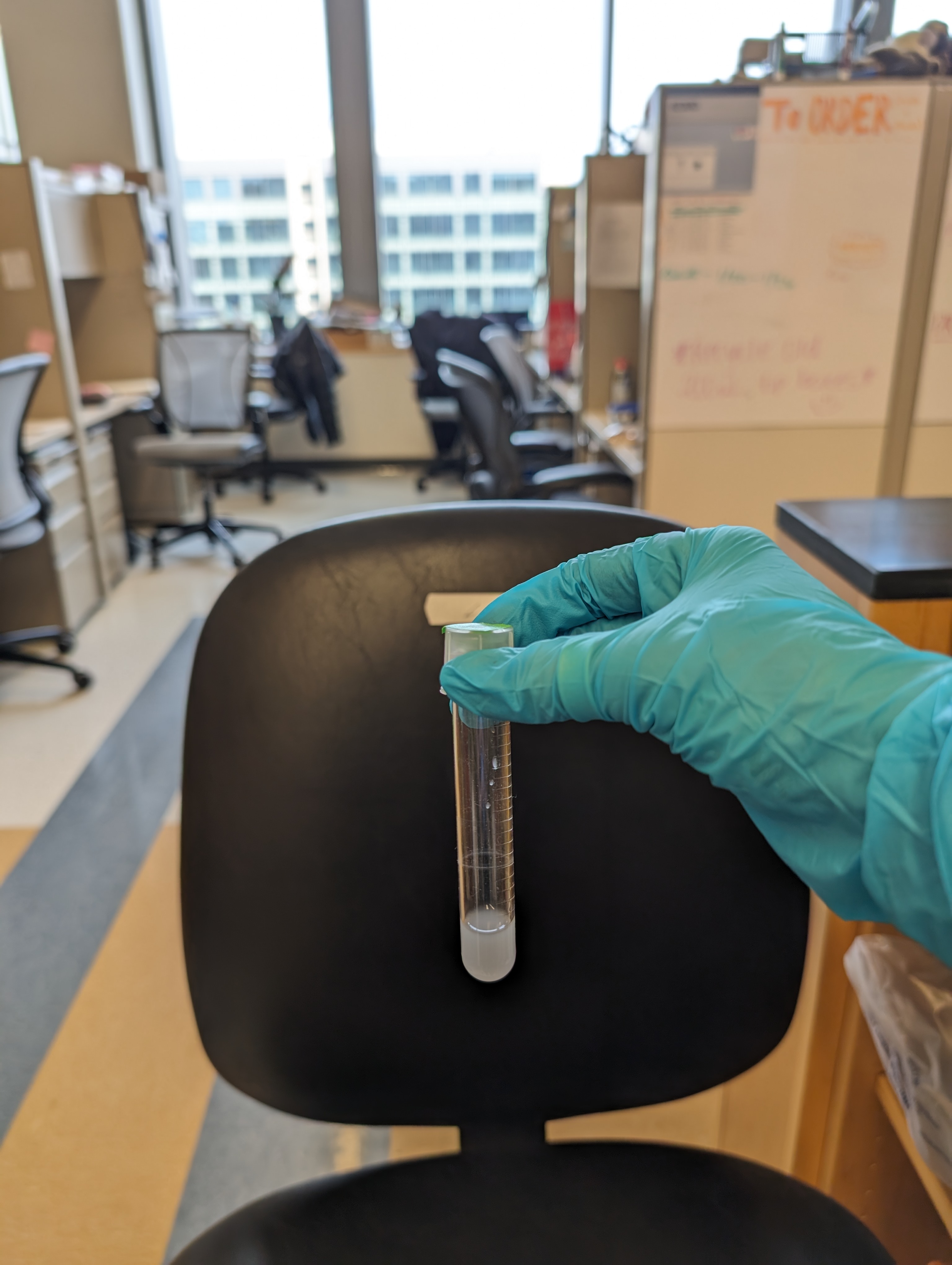

Supplement: S5 File — (ZIP) [file pgen.1011528.s011.zip › Fig 4D/4D frag kan dup- delta bfmR.jpg]

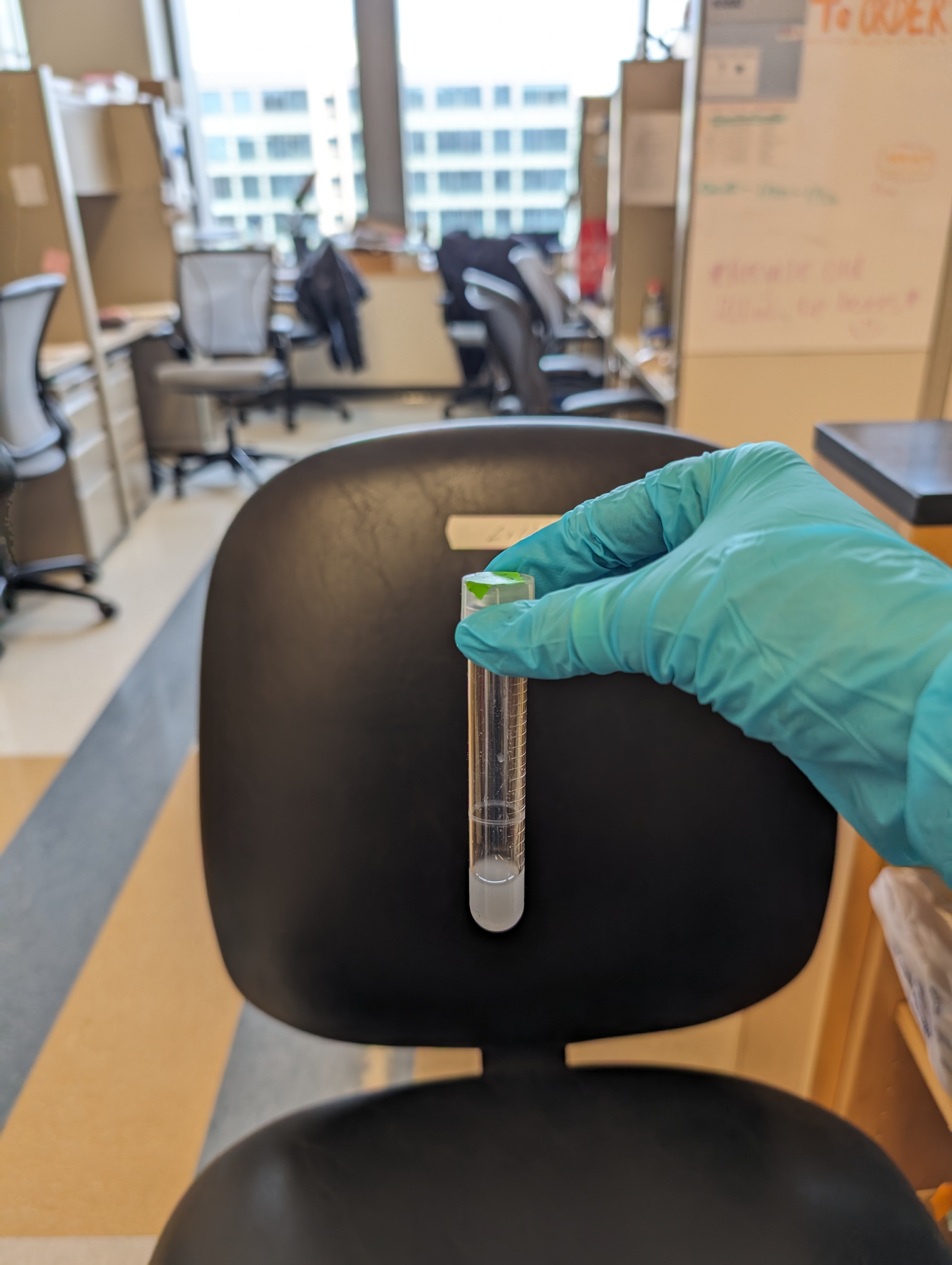

Supplement: S5 File — (ZIP) [file pgen.1011528.s011.zip › Fig 4D/4D frag kan dup- delta bfmS.jpg]

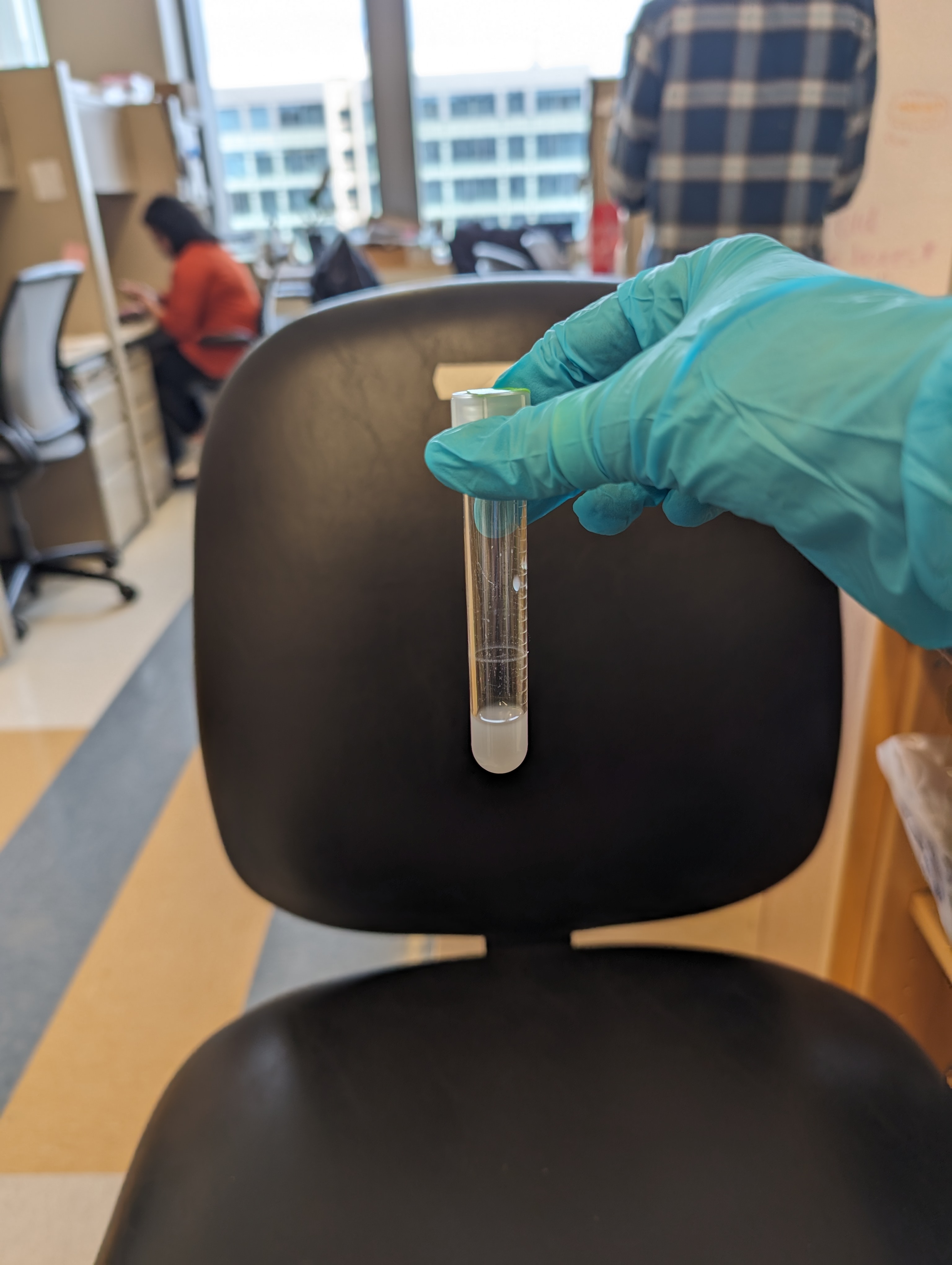

Supplement: S5 File — (ZIP) [file pgen.1011528.s011.zip › Fig 4D/4D frag kan dup- delta bfmSR.jpg]

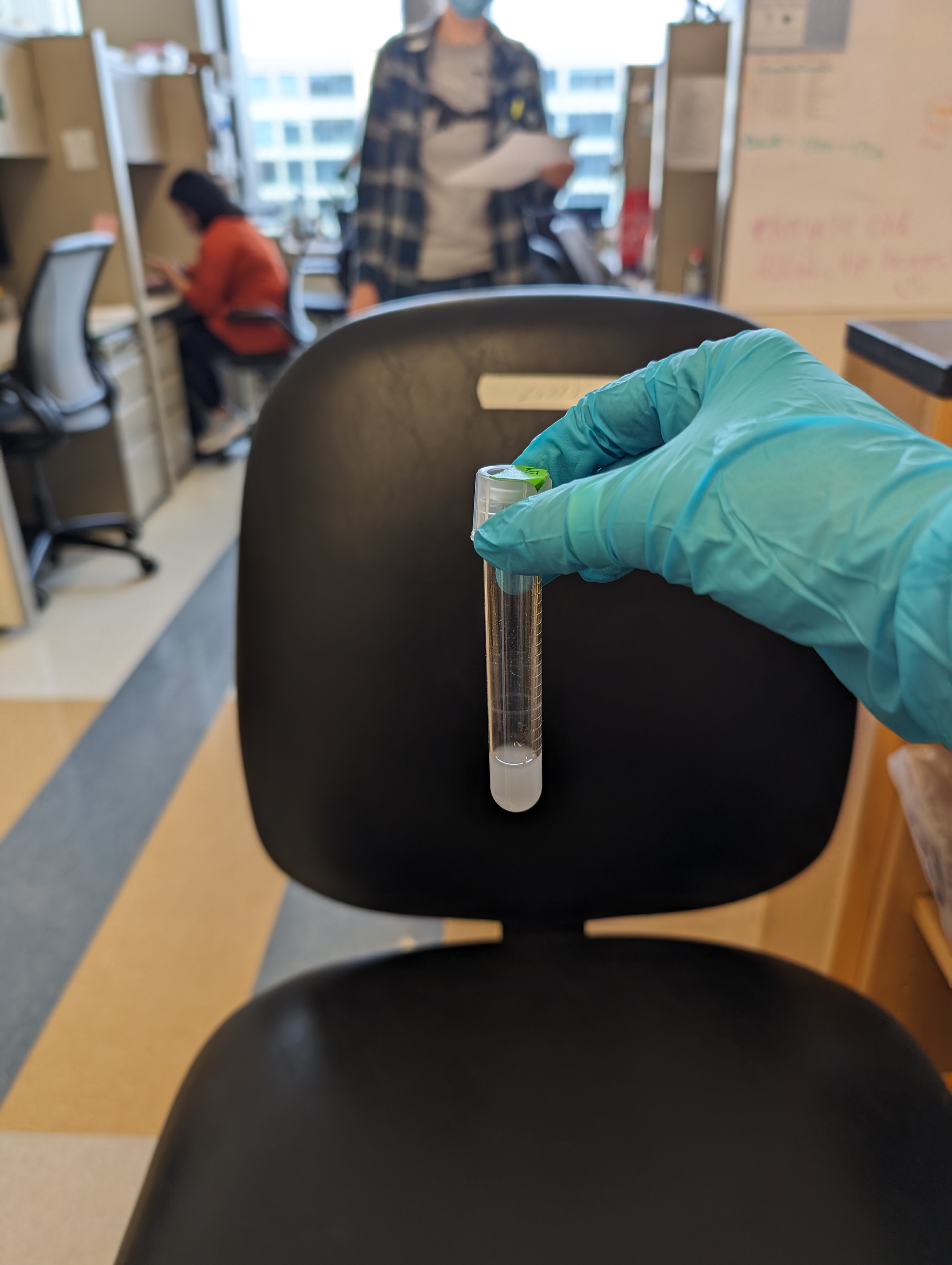

Supplement: S5 File — (ZIP) [file pgen.1011528.s011.zip › Fig 4D/4D frag kan dup- wildtype.jpg]

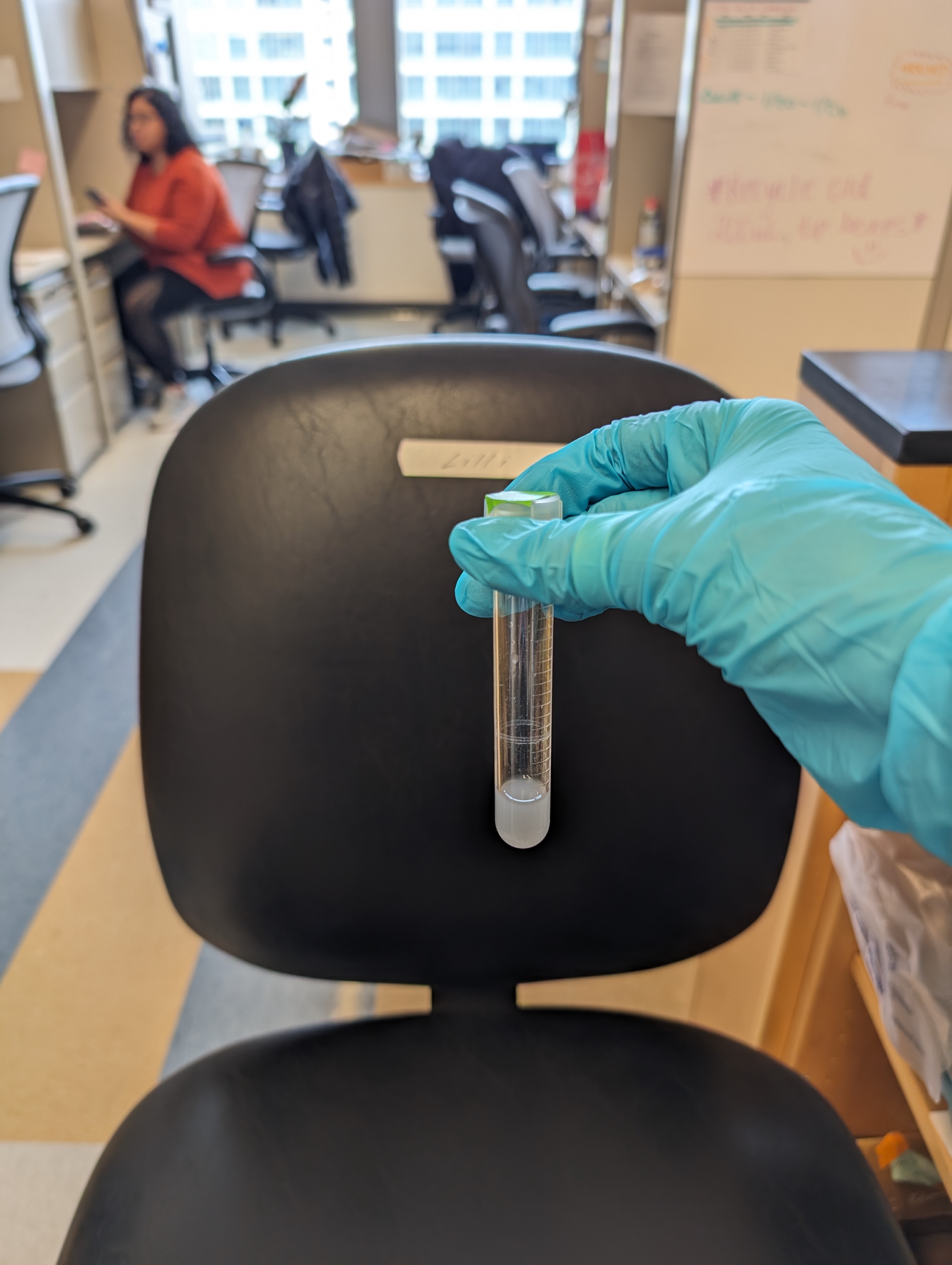

Supplement: S5 File — (ZIP) [file pgen.1011528.s011.zip › Fig 4D/4D frag kan dup+ BfmR(D55A).jpg]

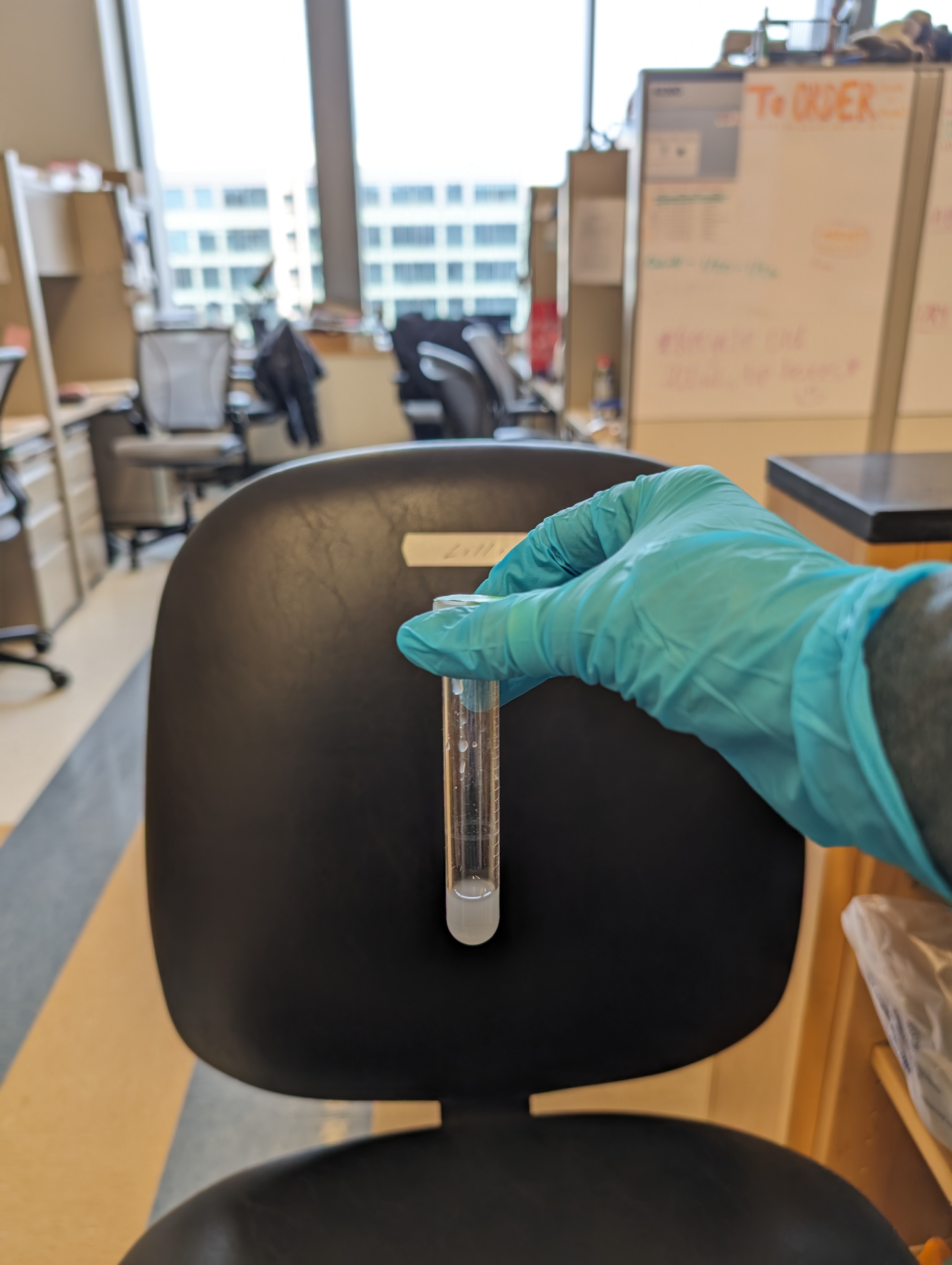

Supplement: S5 File — (ZIP) [file pgen.1011528.s011.zip › Fig 4D/4D frag kan dup+ delta bfmR.jpg]

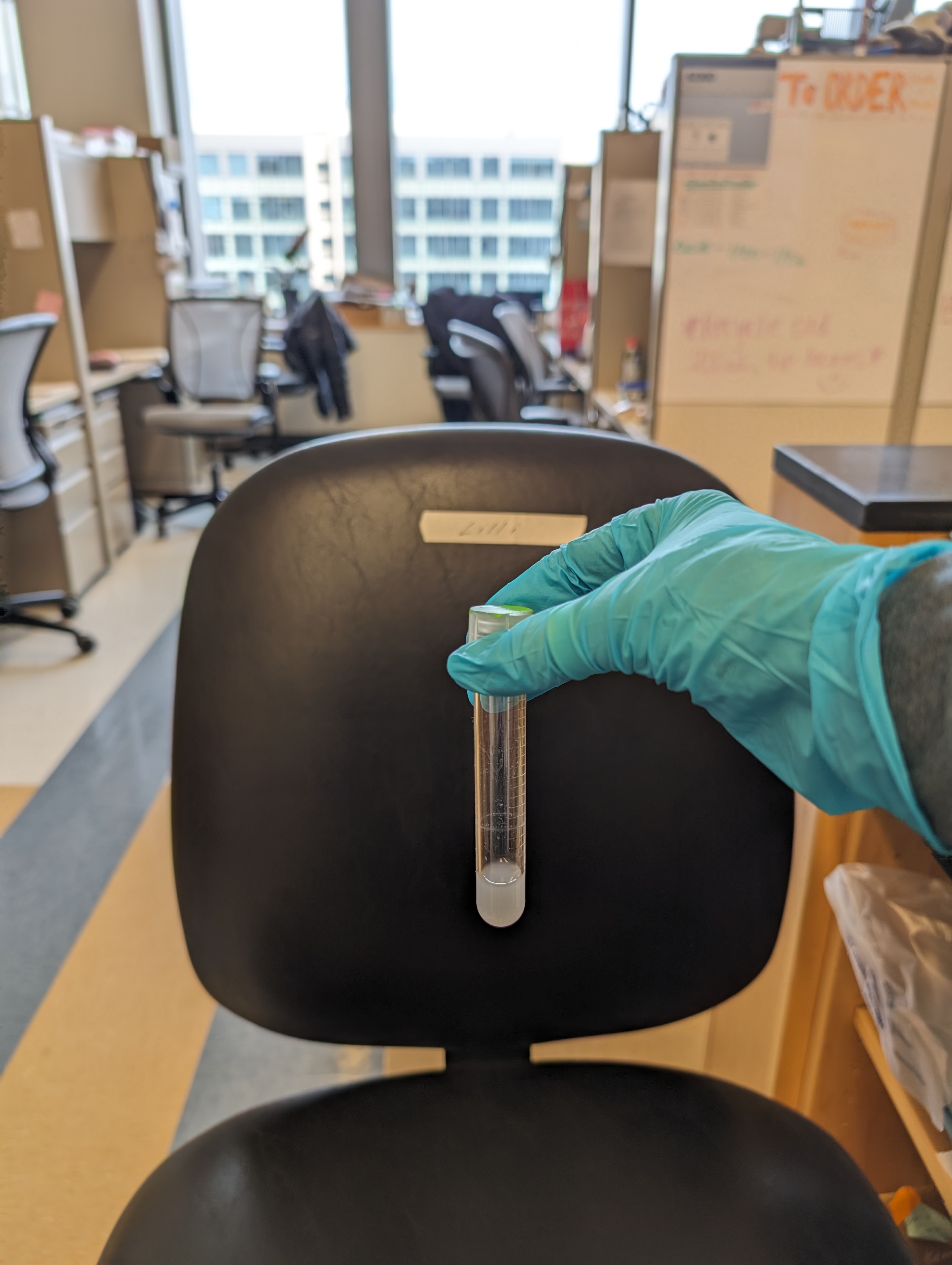

Supplement: S5 File — (ZIP) [file pgen.1011528.s011.zip › Fig 4D/4D frag kan dup+ delta bfmS.jpg]

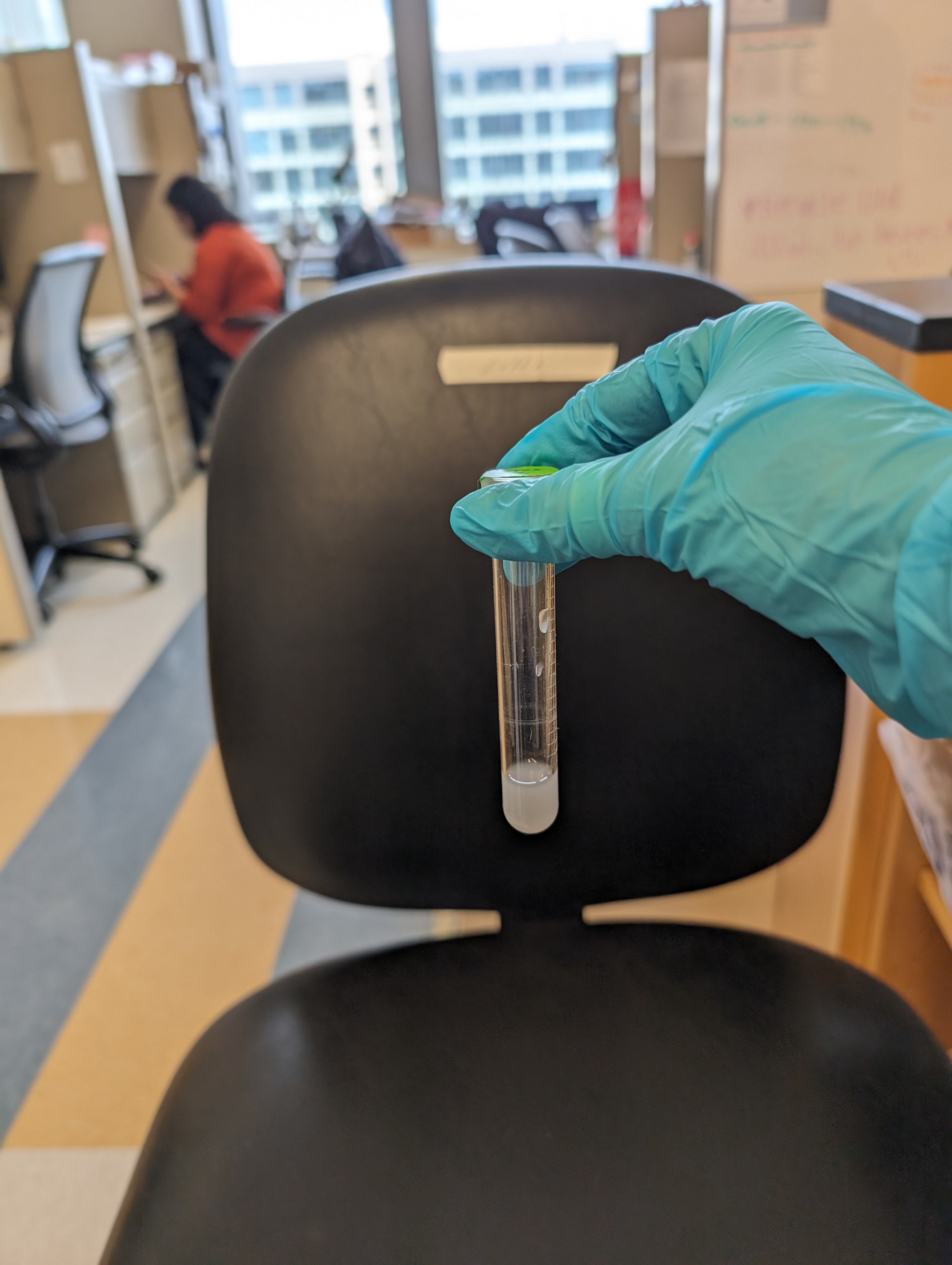

Supplement: S5 File — (ZIP) [file pgen.1011528.s011.zip › Fig 4D/4D frag kan dup+ delta bfmSR.jpg]

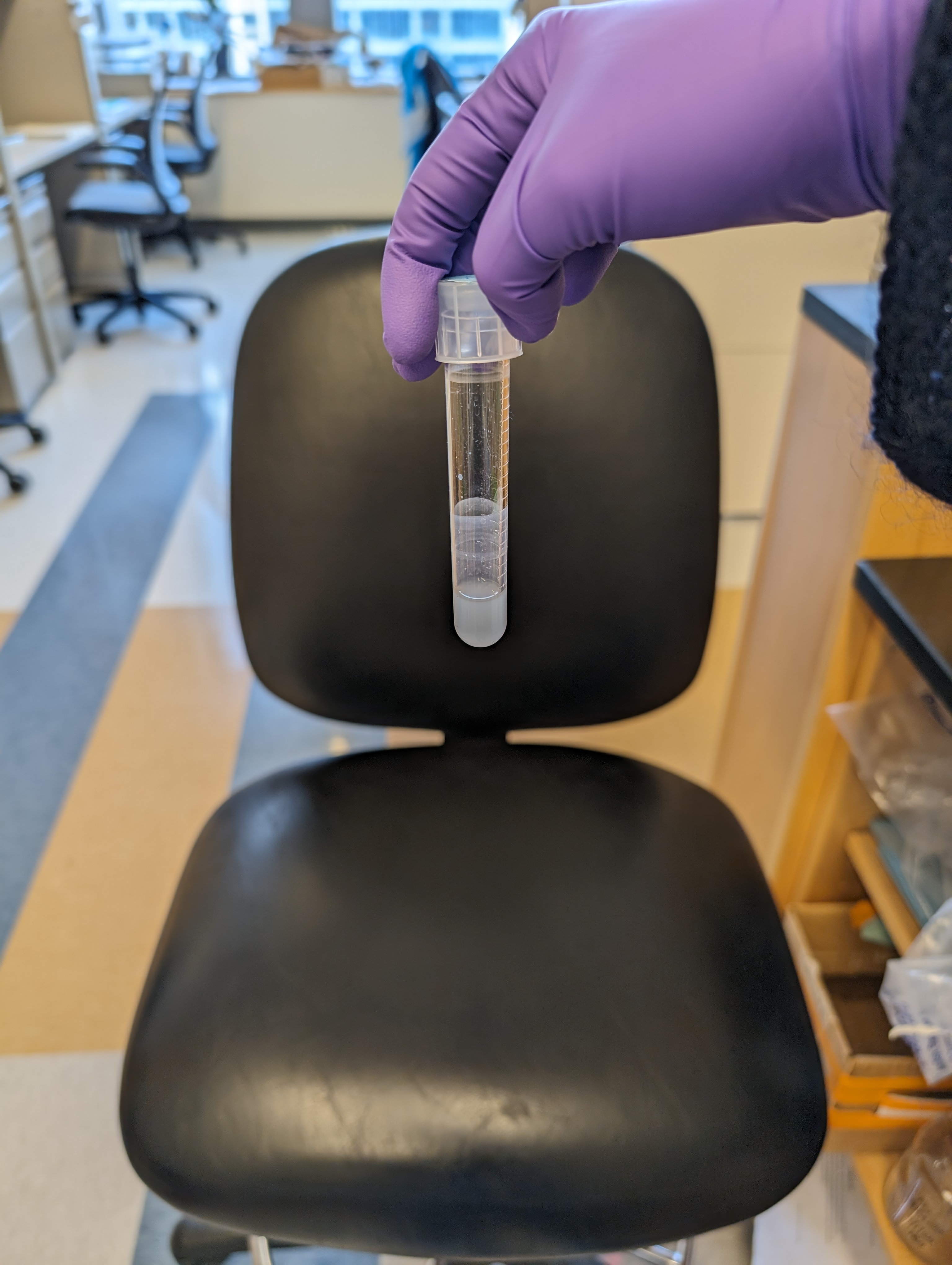

Supplement: S5 File — (ZIP) [file pgen.1011528.s011.zip › Fig 4D/4D frag kan dup+ wildtype.jpg]

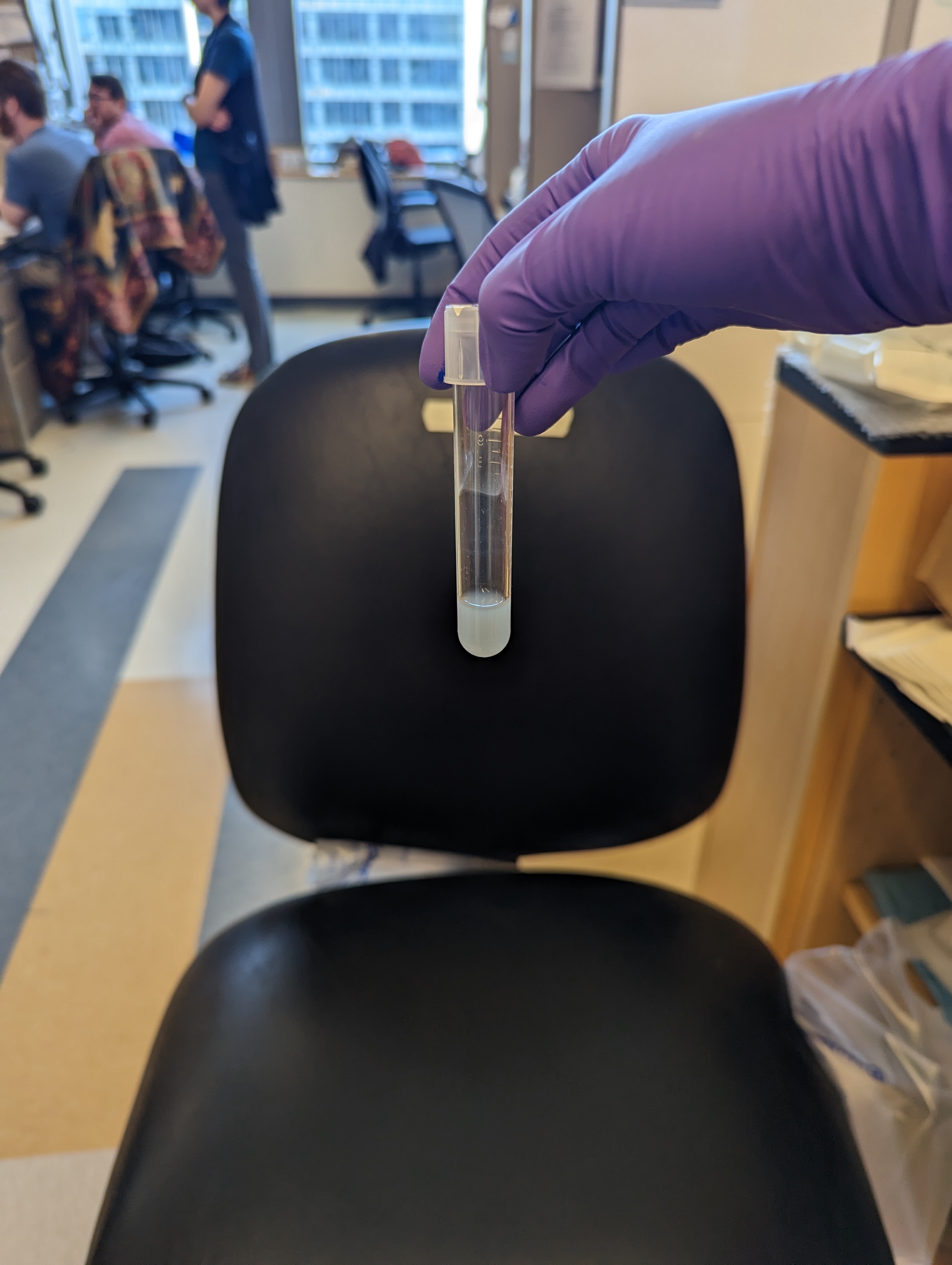

Supplement: S6 File — (ZIP) [file pgen.1011528.s012.zip › Fig 5A/5A delta bfmSR (0 copy).jpg]

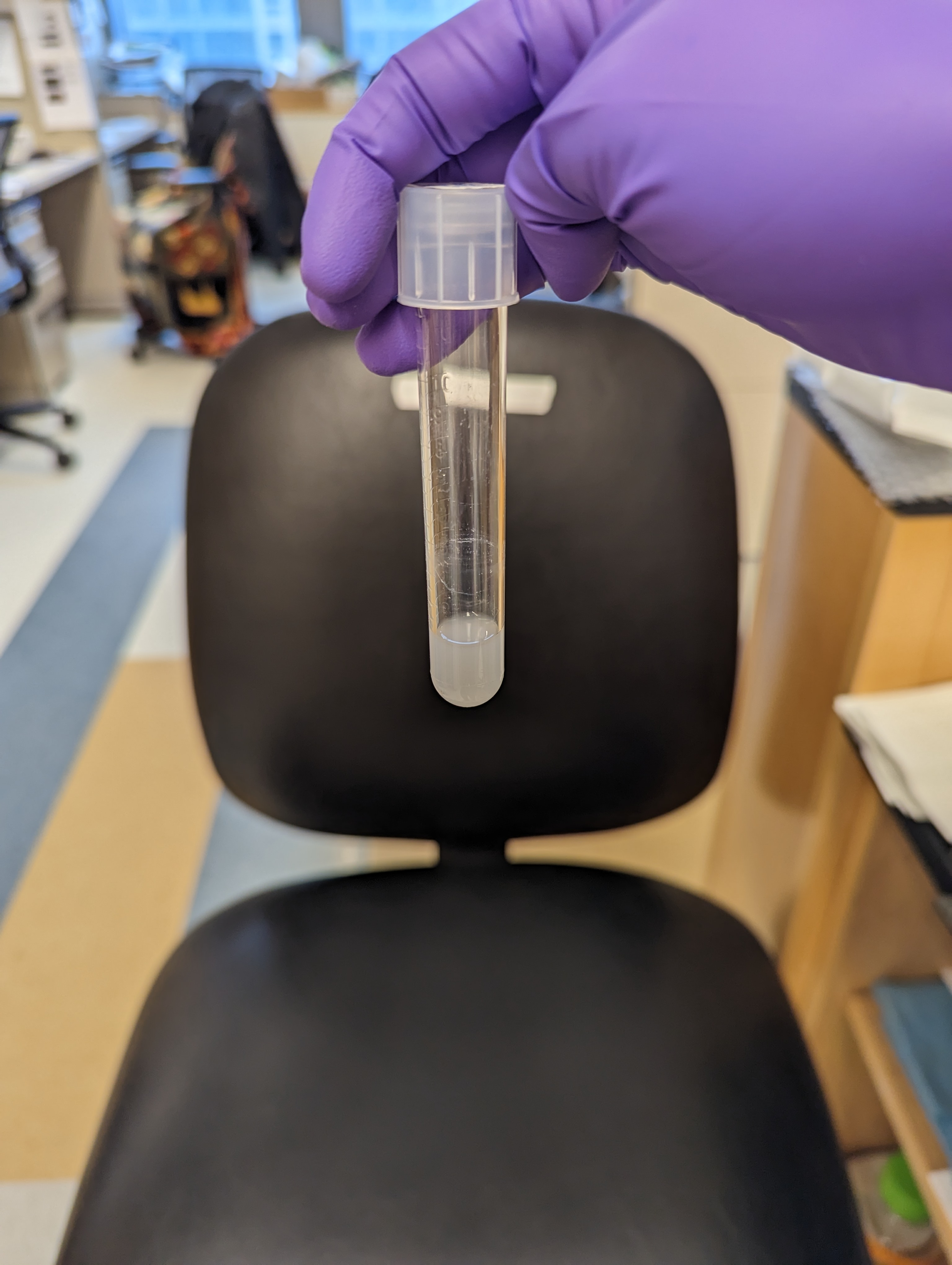

Supplement: S6 File — (ZIP) [file pgen.1011528.s012.zip › Fig 5A/5A delta bfmSR, bfmSRx1 (1 copy).jpg]

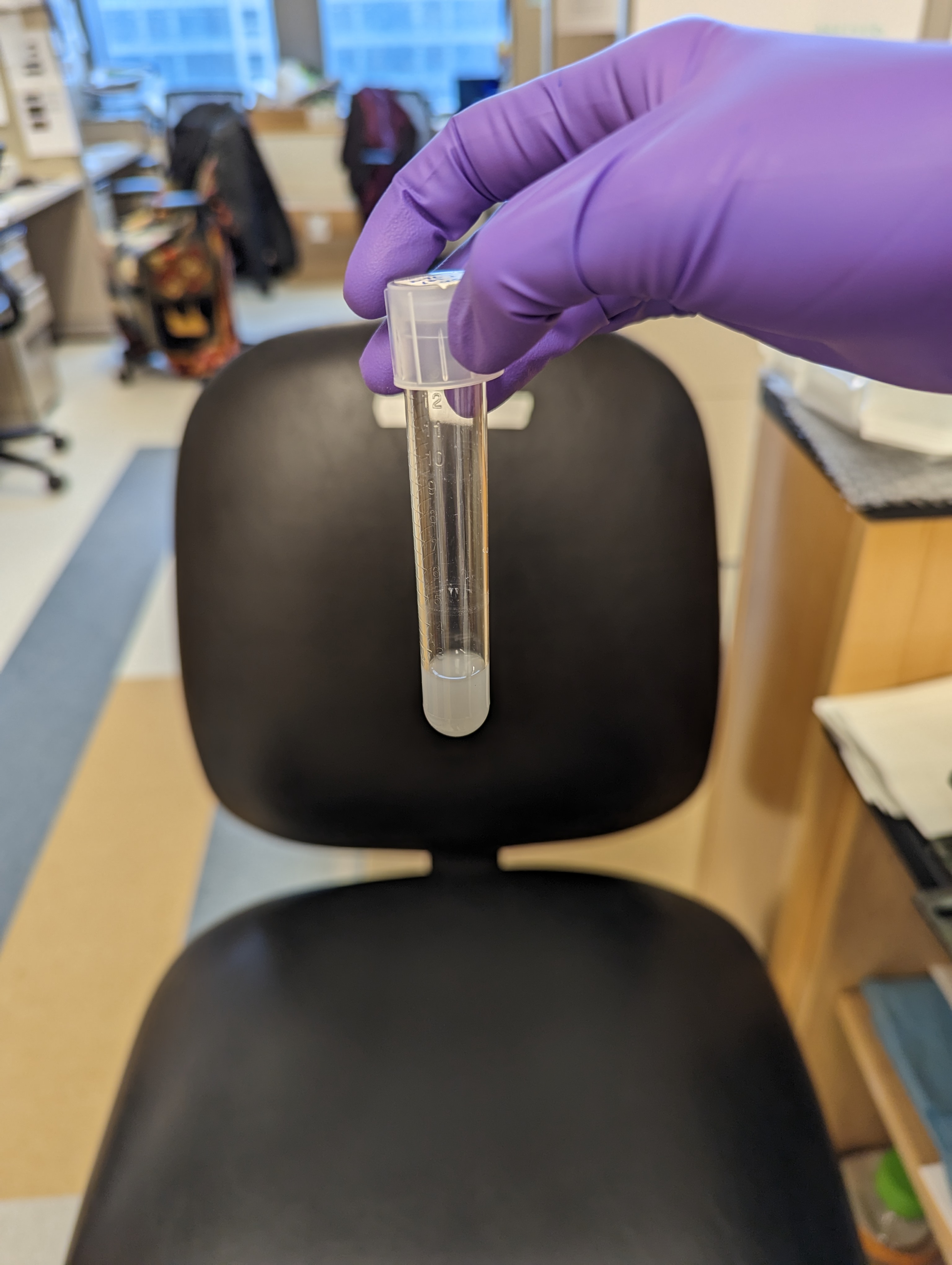

Supplement: S6 File — (ZIP) [file pgen.1011528.s012.zip › Fig 5A/5A delta bfmSR, bfmSRx2 (2 copy).jpg]

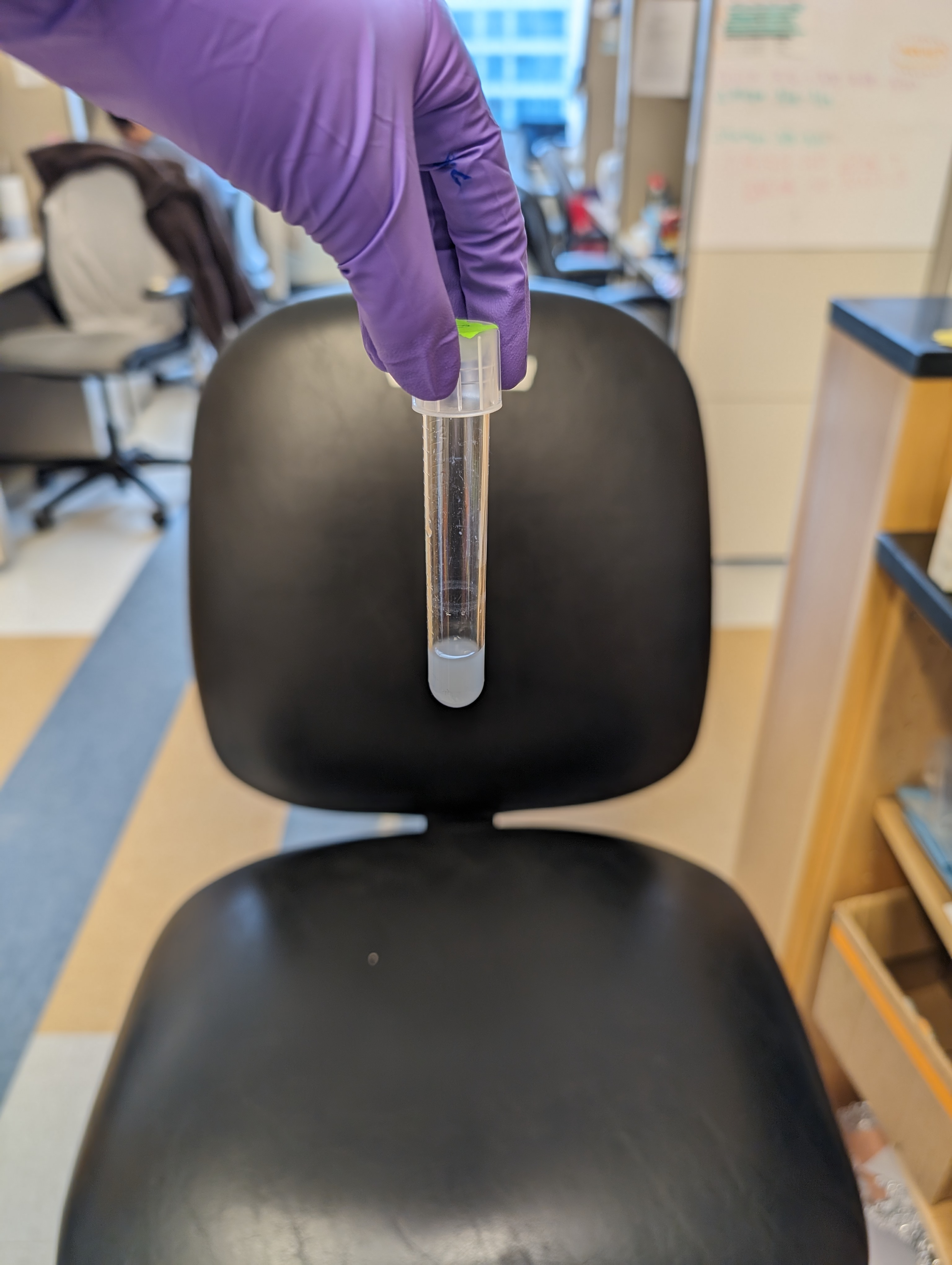

Supplement: S7 File — (ZIP) [file pgen.1011528.s013.zip › Fig 5B/5B delta csuFABCDE,iou,bfmSR (0 copy).jpg]

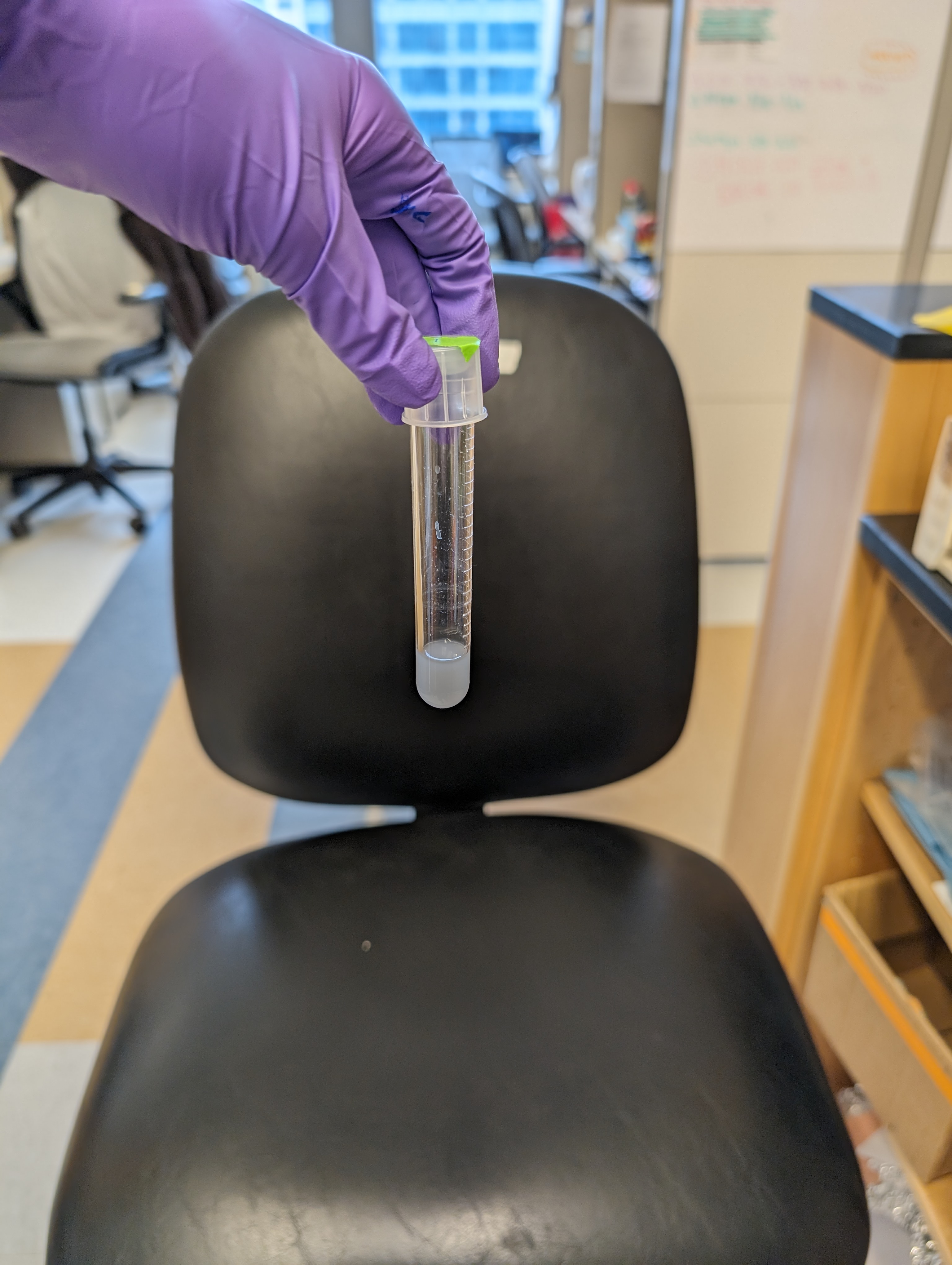

Supplement: S7 File — (ZIP) [file pgen.1011528.s013.zip › Fig 5B/5B delta csuFABCDE,iou,bfmSR, csuFABCDE,iou,bfmSRx1 (1 copy).jpg]

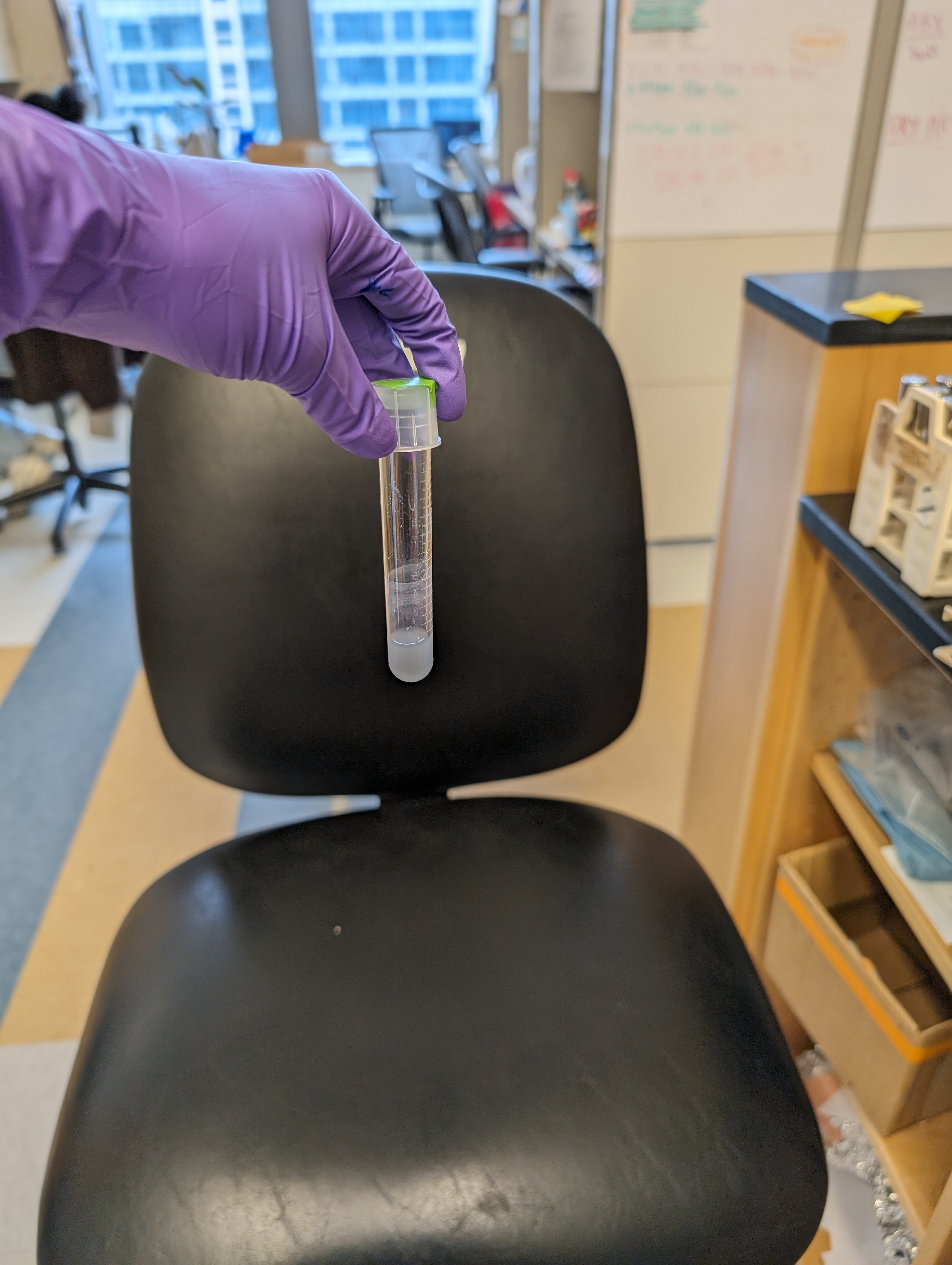

Supplement: S7 File — (ZIP) [file pgen.1011528.s013.zip › Fig 5B/5B delta csuFABCDE,iou,bfmSR, csuFABCDE,iou,bfmSRx2 (2 copy).jpg]

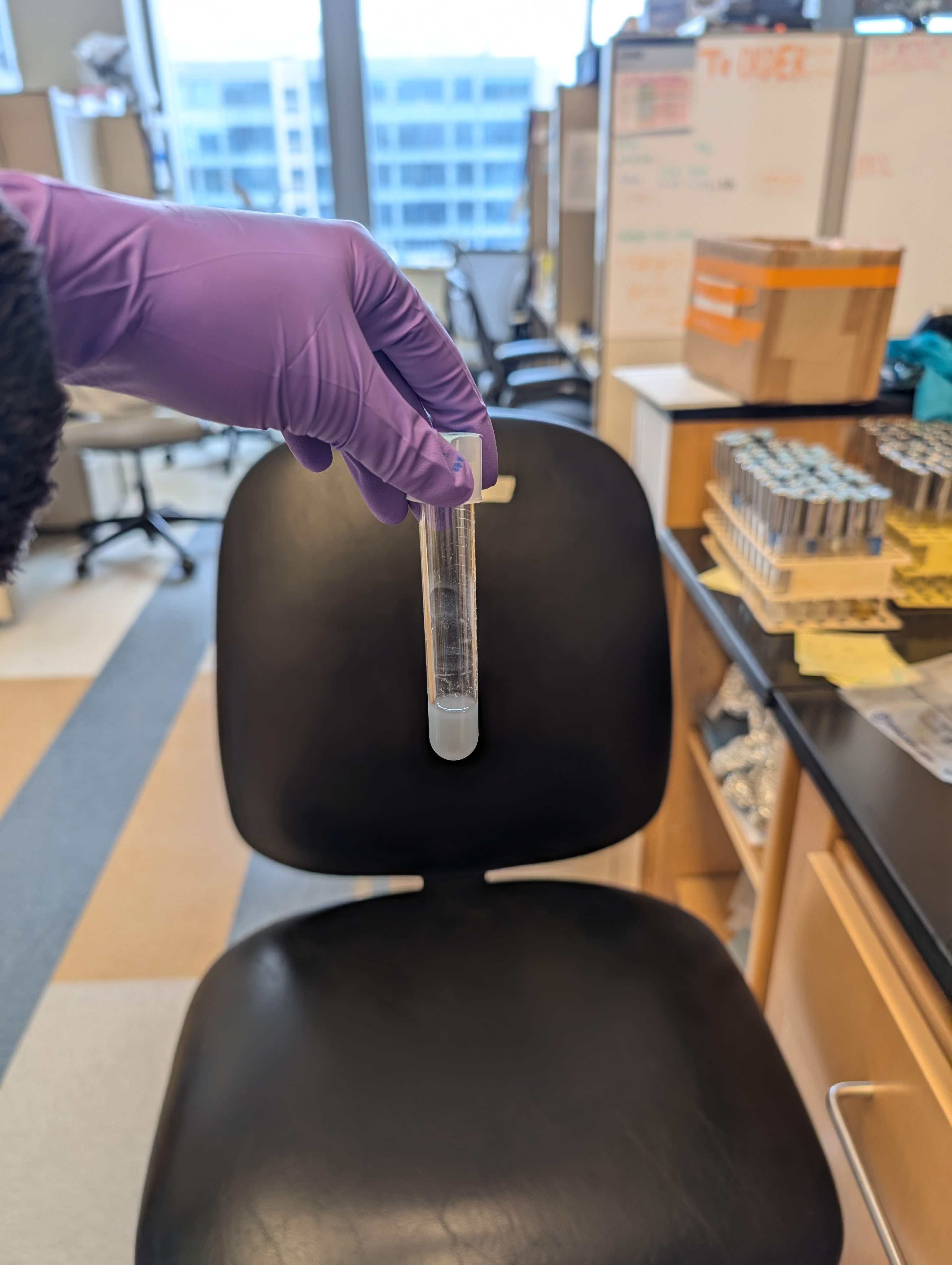

Supplement: S8 File — (ZIP) [file pgen.1011528.s014.zip › Fig 5C/5C delta bfmSR (0 copy).jpg]

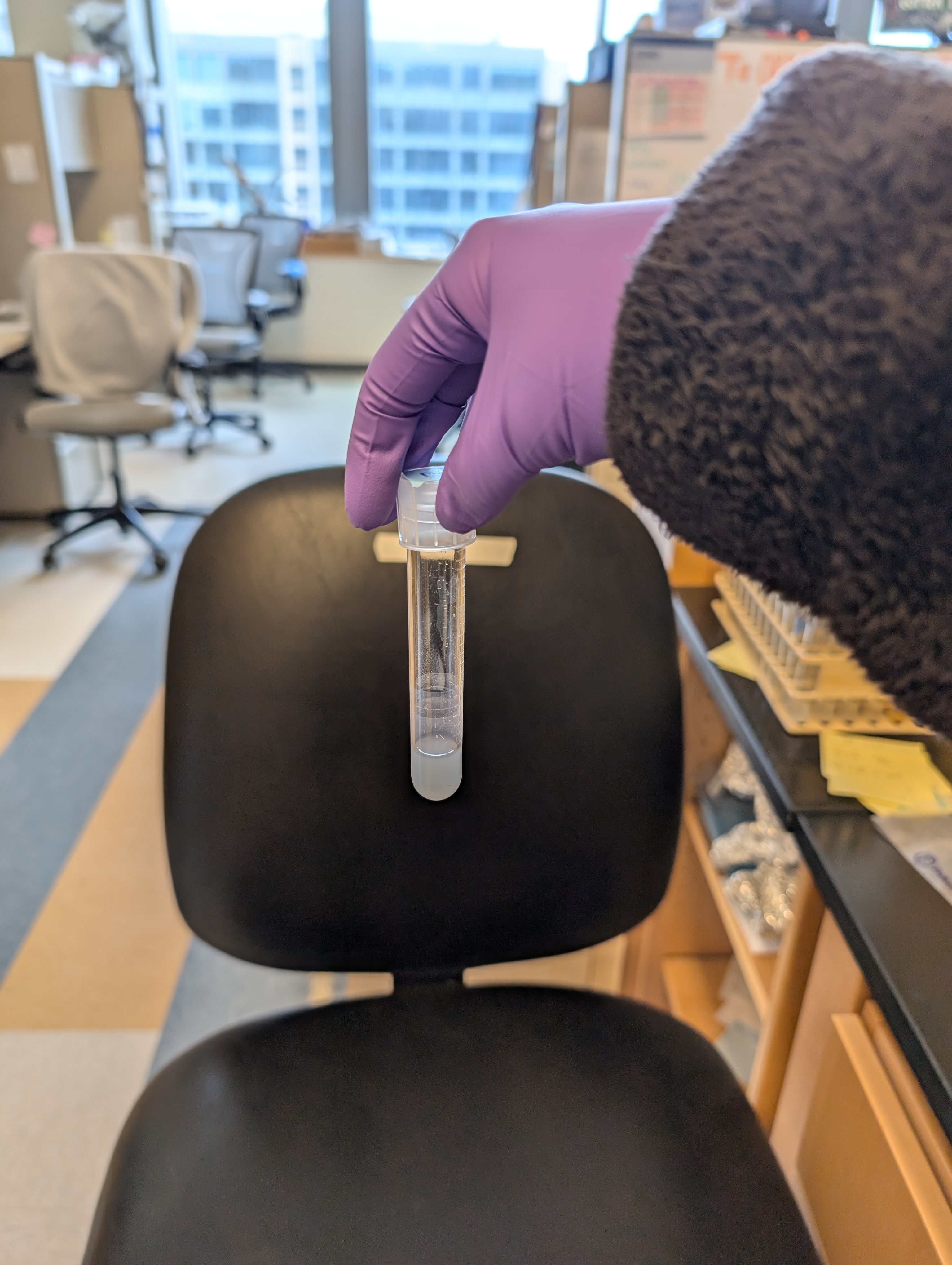

Supplement: S8 File — (ZIP) [file pgen.1011528.s014.zip › Fig 5C/5C delta bfmSR, csuF'-'csuE,iou,bfmSRx1 (1 copy).jpg]

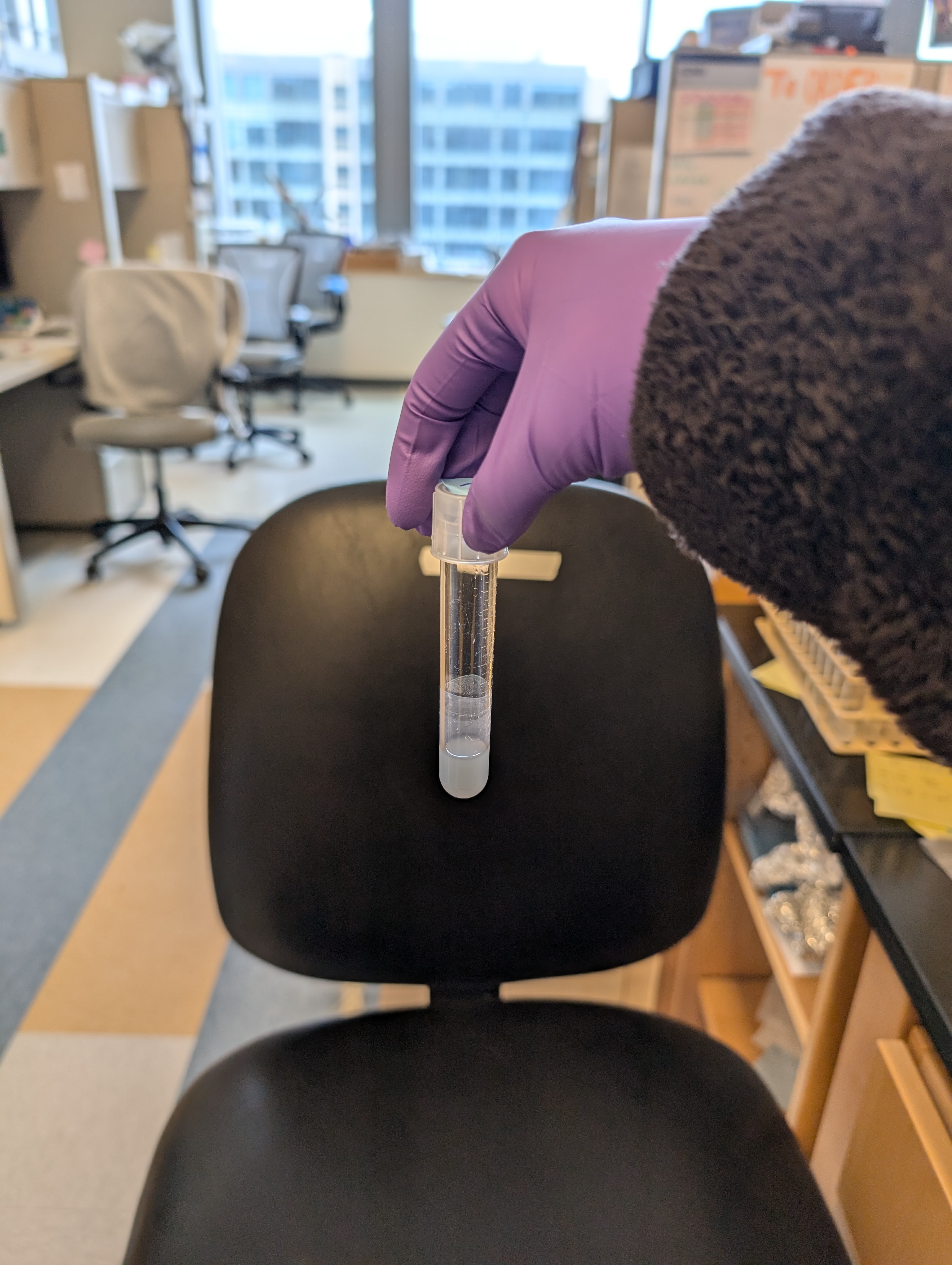

Supplement: S8 File — (ZIP) [file pgen.1011528.s014.zip › Fig 5C/5C delta bfmSR, csuF'-'csuE,iou,bfmSRx2 (2 copy).jpg]
